# Design, molecular modelling and synthesis of novel benzothiazole derivatives as BCL-2 inhibitors

**DOI:** 10.1038/s41598-023-41783-1

**Published:** 2023-09-20

**Authors:** Hoda S. Ismail, Amira Khalil, Rabah A. Taha, Deena S. Lasheen, Dalal A. Abou El Ella

**Affiliations:** 1 Pharmaceutical Chemistry Department, Faculty of Pharmacy, Ain Shams University, Cairo, Egypt; 2 Pharmaceutical Chemistry Department, Faculty of Pharmacy, The British University in Egypt (BUE), El-Sherouk City, Cairo, 11837 Egypt

**Keywords:** Drug discovery, Chemistry

## Abstract

Apoptosis plays a crucial role in cancer pathogenesis and drug resistance. BCL-2 family of enzymes is considered as one of the key enzymes which is involved in apoptosis. When there is disruption in the balance between anti-apoptotic and pro-apoptotic members of the BCL-2 family apoptosis is dysregulated in the affected cells. Herein, 33 novel benzothiazole-based molecules **7a-i**, **8a-f**, **9a-b**, **12a-e**, **13a-d**, **14a,b**, and **17a-j** were designed, synthesized and tested for their BCL-2 inhibitory activity. Scaffold hopping strategy was applied in designing of the target compounds. Compounds **13c** and **13d** showed the highest activity with IC_50_ values equal to 0.471 and 0.363 µM, respectively. Molecular docking studies of the synthesized compounds showed comparable binding interactions with the lead compound. Structure activity relationship study was performed to show the effects of structural modifications on the inhibitory activities on BCL-2.

## Introduction

Cancer is considered a leading cause of death worldwide accounting for about 14.5% of world deaths, and it is expected for that ratio to double by 2030^1^. The concept of "Hallmarks of Cancer" is a powerful guide for research aimed to improving and developing rational approaches for more targeted cancer therapeutics^2^. The ability of cancer cells to resist cell death and evade apoptosis, which leads to continuous proliferation, is one of the fundamental hallmarks of cancer. Therefore, it has become a major area of interest in the development of targeted cancer therapy.

Apoptosis is a physiological process of programmed cell death that is essential for normal tissue development and hemostasis^3^. Aberrations in this pathway can lead to a variety of diseases including degenerative and autoimmune disorders and cancer^4,5^.

Caspases is a family of enzymes that plays a critical role in apoptosis regulation^6^. Caspases are synthesized as inactive zymogens, which undergo activation through cascade of events known collectively as either intrinsic or extrinsic pathways^7,8^.

Cancer cells may evade apoptosis through a variety of mechanisms. In human cancer cells, the downregulation of pro-apoptotic proteins (e.g. Bax, Bak, Bad, Bim) and the upregulation of anti-apoptotic proteins (e.g. BCL-2, BCL-xl, MCL-1), inhibits the release of cytochrome *c* from mitochondria, leads to the immortal character of the cancer cells^9^. In cancer cell lines, all the apoptotic inhibitors and activators have been found to be expressed abnormally. For instance, in almost half of all human cancers, BCL-2 overexpression has been found^10^.

BCL-2 family proteins are the key regulators of the mitochondrial apoptotic pathway, also known as the BCL-2-regulated pathway. There are 25 members that belong to the BCL-2 family of proteins. These proteins are found in mitochondria, smooth endoplasmic reticulum, and perinuclear membranes in hematopoietic cells^11,12^. The structure of BCL-2 proteins are known to exhibit up to four relatively short sequence motifs, which are less than 20 amino acid residues in length, termed BCL-2 homology (BH) domains^13,14^. All BCL-2 family proteins present BCL-2 homology (BH) domains, with the most conserved BH3 death domain being found in all members of the family. Members of BCL-2 family can be divided into three subfamilies based on structural and functional features^15–17^ which include anti-apoptotic multi-domain proteins, pro-apoptotic BH3-only proteins and pro-apoptotic multi-domain effector proteins. The functions of BCL-2 family members including the anti-apoptotic and pro-apoptotic are regulated through their BH domains. Furthermore, the BH1- BH3 domains of anti-apoptotic proteins form a hydrophobic binding pocket that binds the α-helix of the BH3-only pro-apoptotic proteins^18,19^. If there is imbalance between the anti-apoptotic and pro-apoptotic members of the BCL-2 family, it will result in dysregulated apoptosis in the affected cells. This could attributed to the expression of one or more anti-apoptotic proteins or the low expression of one or more pro-apoptotic proteins or a combination of both^20^.

The selective modulation of both apoptotic pathways has been reported to be a challenge in cancer drug development. BCL-2 functions by preventing programmed cell death which differs from other oncogenes that work mainly by enhancing proliferation^21^. Therefore, inhibiting these proteins would lead to apoptosis induction which could provide potential therapeutic target^22^. However, the critical challenge is that many of these targets are protein–protein interactions and it is hard to modulate. Despite this, there are various promising molecules which target the apoptotic pathway components that have now entered the clinic, and are under investigation as single agents or in combination with other anticancer therapies^23^. Anti-sense oligonucleotides (ASOs), peptides and peptidomimetics as well as small molecules (BH3 mimetics) are among the promising BCL-2 Inhibitors. Despite the emergence of various BCl-2 inhibitors, there are many challenges that are facing these inhibitors, including the development of resistance and limitation of their use in certain types of tumors, e.g., solid tumors. These challenges encourage many researchers to search, design and synthesize novel BCl-2 inhibitors with better activity, less resistance and safer profile^24^.

**Figure 1:**
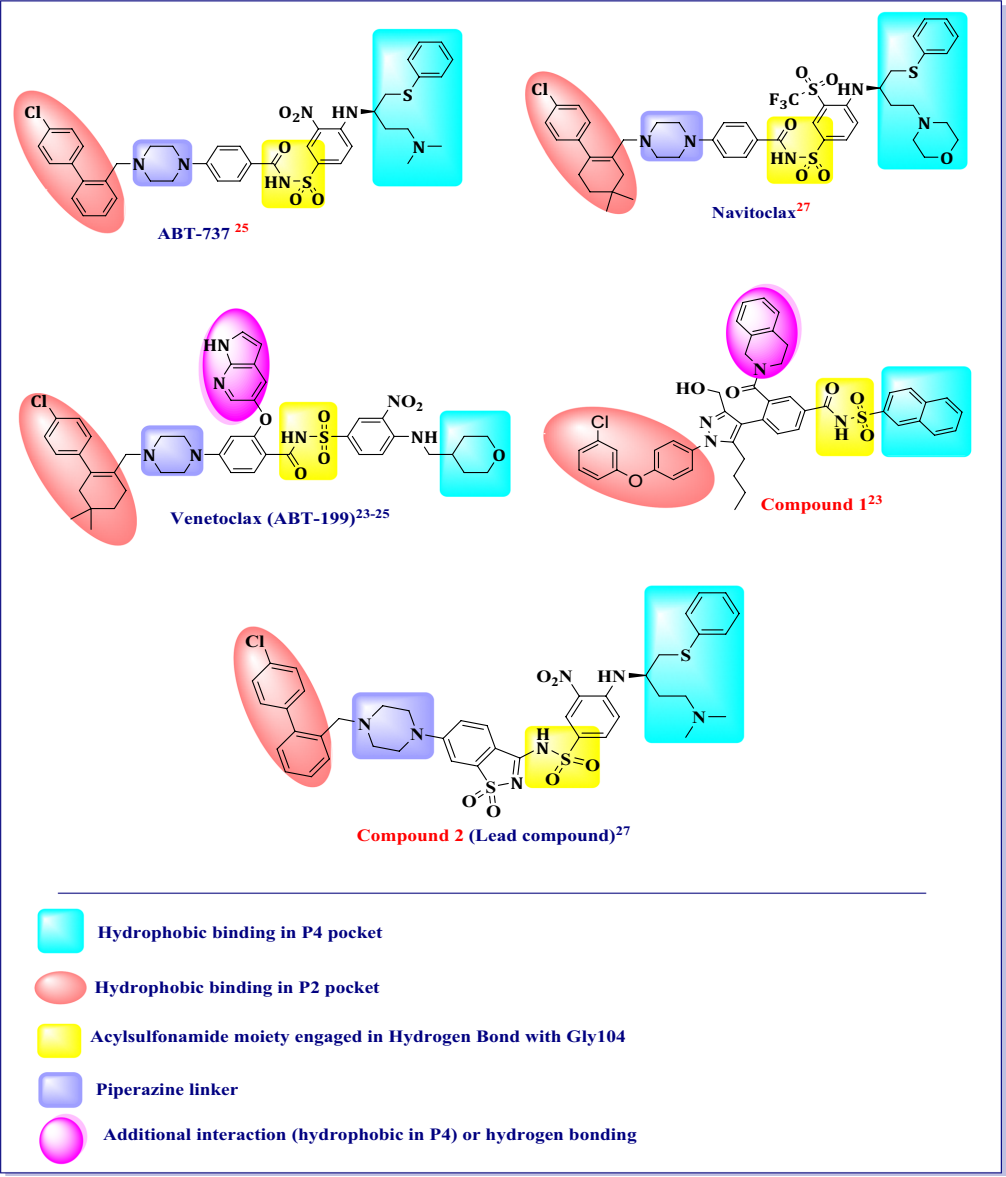
The common structural features and the possible key interactions of prominent BCL-2 inhibitors with BCL-2 protein. Figure 1 was generated using Chemdraw Professional 16.0.

Figure 1 shows some of BCl-2 inhibitors in which the structural features and the important interactions are highlighted^25–29^.

## Rationale and design

**Figure 2:**
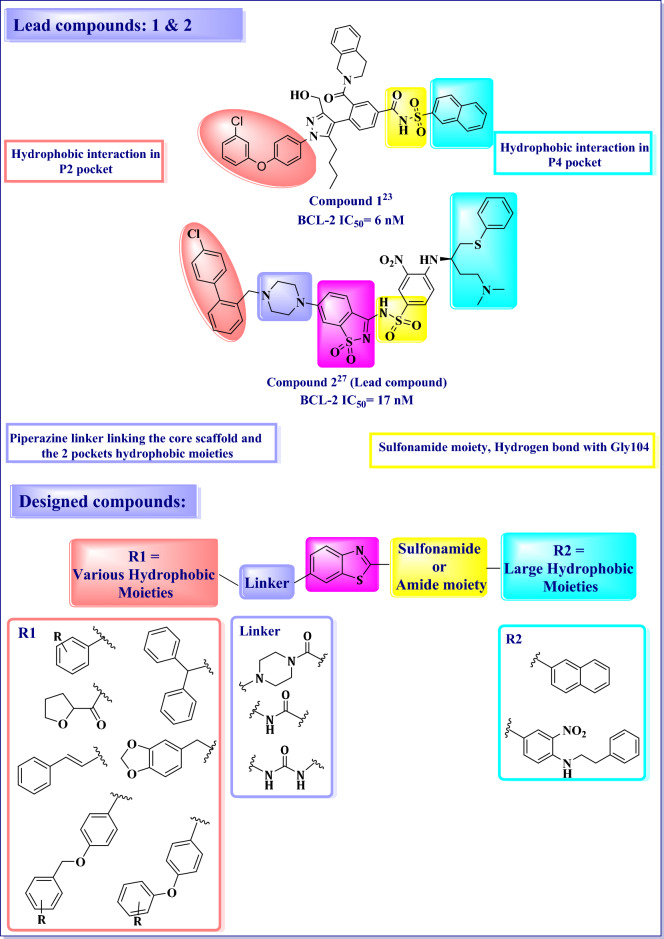
Proposed design of novel benzothiazole based small molecule BCL-2 inhibitors. Figure 2 was generated using Chemdraw Professional 16.0.

Our proposed design of novel benzothiazole based inhibitors was according to the strategies shown in (Fig. 2). Scaffold hopping strategy has been used for designing structurally novel compounds by modifying the central core of an active molecule. Thus, the saccharine scaffold in the lead compound **2** has been replaced by benzothiazole scaffold. The introduction of heterocyclic core will lead to increased acidity of the sulfonamide NH which is proposed to influence potency, solubility, and clearance of these inhibitors. Additionally, the benzothiazole scaffold will maintain the proper orientation of the two essential hydrophobic pocket binding moieties of the compound. The sulfonamide moiety has been retained in most of proposed compounds. In other compounds, it has been replaced by its amide bioisostere to maintain crucial hydrogen bond interactions. Furthermore, a number of diverse small and large hydrophobic moieties with different substituents have been utilized, which maintain the reported hydrophobic interaction with P2 pocket, to explore the hydrophobic moiety with optimum BCL-2 inhibitory activity. Similarly, based on the reported hydrophobic groups used as the P4 binding moieties, the naphthyl (as in compound **1**) and 3-nitro-4-(phenethylamino)benzene (to mimic the P4 hydrophobic moiety in compound **2**) have been used to maintain desirable hydrophobic interaction with P4 pocket and to explore the hydrophobic moiety with optimum BCL-2 inhibitory activity. Finally, the piperazine linker between the core scaffold and the P2 hydrophobic moiety has been retained in some proposed compounds or replaced by other linkers such as urea and amide linkers in other compounds to explore their impact on BCL-2 inhibitory activity.

The aim of the current study is to rationally design, synthesize and biologically evaluate novel small molecule BCL-2 inhibitors (BH3 mimetics) via targeting the BH3 binding groove of anti-apoptotic BCL-2 protein as targeted anti-cancer therapy.

## Materials and methods

### Chemistry

Starting materials and reagents were purchased from Sigma-Aldrich or Alfa-Aesar Organics and used without further purification. Reactions were monitored by analytical TLC, performed on silica gel 60 F_254_ packed on Aluminum sheets, purchased from Merck, with visualization under UV light (254 nm). Melting points were recorded on Stuart Scientific apparatus and are uncorrected. Electron-impact ionization mass (EI-MS) spectra were recorded on Thermo Scientific ISQLT mass spectrometer at the Regional Center for Mycology and Biotechnology, Al-Azhar University.

^1^H-NMR spectra were recorded using Bruker 400 MHz spectrophotometer (using DMSO as solvent) at Center for Drug Discovery Research and Development, Ain Shams University. ^13^C -NMR spectra were recorded in δ scale given in ppm on a Bruker 100 MHz spectrophotometer (at 101 MHz, using DMSO as solvent) at Center for Drug Discovery Research and Development, Ain Shams University. Elemental analyses were performed on a Thermo Scientific Flash 2000 elemental analyzer at the Regional Center for Mycology and Biotechnology, Al-Azhar University.

For the detailed synthetic procedures and structural characterization, refer to the supporting information.

### Biological evaluation

#### In vitro BCL-2 inhibitory activity

The in vitro enzyme inhibition determination for the synthesized compounds was carried out in BPS Bioscience Corporation, San Diego, CA, USA (www.bpsbioscience.com). BCL-2: Catalog # 50272, BCL-2 binding peptide, BCL-2 Assay Kit: Catalog #50222, and Tb-Donor and Dye labeled acceptor were used.

##### Assay protocol

The assay was performed by TR-FRET technology using a recombinant BCL-2 and a peptide- ligand substrate. The TR-FRET signal from the assay is correlated with the amount of Ligand binding to BCL-2. Compounds were diluted in 100% DMSO then tenfold dilution in 10% DMSO in 1X Reaction Buffer. 2 µl of the dilution was added to a 20 µl reaction so that the final concentration of DMSO is 1% in all of reactions. All of the binding reactions were conducted at room temperature. The 20 µl reaction mixture in Assay Buffer contains bcl-2, the indicated amount of the inhibitor, ligand, and the reaction dyes. The reaction mixture incubated for 180 min prior to reading the TR-FRET signal. For the background, ligand was replaced with assay buffer. Fluorescence signals for both the donor and acceptor dyes were measured using a Tecan Infinite M1000 plate reader. TR-FRET was recorded as the ratio of the fluorescence of the acceptor and the donor dyes (acceptor/donor).

Binding experiments were performed in duplicate at each concentration. The TR-FRET data were analyzed using the computer software, Graphpad Prism. In the absence of the compound in wells containing BCL-2 ligand, the TR-FRET signal (Ft) in each data set was defined as 100% activity. In wells without peptide ligand, the TR-FRET signal (Fb) in each data set was defined as 0% activity. The percent activity in the presence of each compound was calculated according to the following equation: % activity = [(F − Fb)/ (Ft − Fb)] × 100, where F = the TR-FRET signal in the presence of the compound. The percent inhibition was calculated according to the following equation: % inhibition = 100—% activity. IC_*50*_ determination for target compounds against BCL-2 was calculated. The values of % activity versus a series of compound concentrations (1 nM—10 nM—100 nM—1 µM—10 µM) were then plotted using non-linear regression analysis of Sigmoidal dose–response curve generated with the equation:

$$ {\text{Y}} = {\text{ B }} + \, \left( {{\text{T}} - {\text{B}}} \right)/{1} + {1}0^{{\left( {\left( {{\text{LogEC5}}0 - {\text{X}}} \right) \, \times {\text{ Hill}}\,{\text{ Slope}}} \right)}} , $$

where, Y = percent activity, B = minimum percent activity, T = maximum percent activity, X = logarithm of compound and Hill Slope = slope factor or Hill coefficient. The IC_50_ value was determined by the concentration causing a half-maximal percent activity^30^.

#### In vitro anti-proliferative activity against NCI leukemia cell line

The national cancer institute (NCI) in vitro anticancer screening is a two-stage process, beginning with the evaluation of the selected compounds **13c** and **13d** against NCI leukemia cell line at a single dose of 10 µM. The human leukemia cell line was obtained and maintained at the NCI. The output from the single dose screen is reported as a mean graph. The detailed assay protocol is discussed in the supporting material.

### Molecular modelling study

Molecular docking was conducted using C-Docker 2.5 software in the interface of Accelry’s discovery studio 2.5 (Accelrys Inc., San Diego, CA, USA) at the drug design laboratory of Faculty of pharmacy, Ain Shams university. The X-ray crystal structure of BCL-2 co-crystallized with saccharine-based lead compound **2** was obtained from the Protein Data Bank^31^ with PDB code: **4IEH**^29^. This PDB code was chosen to compare the docked poses of our designed compounds to the lead compound **2**. Protein was prepared in Discovery Studio 2.5 by deleting water molecules and adding hydrogen atoms. A final step of energy minimization for hydrogen atoms was done to relieve the constraints resulting from randomly adding hydrogen atoms with constraining protein heavy atoms. Docking search space was identified by assigning a sphere centred around the co-crystallized ligand including the surrounding amino acids of the binding site. The docking sphere had a centre at coordinates 12.2, 25.8, 11.8, and a radius of 60 Å.

Ligands were sketched in ChemDraw and minimized in Discovery Studio 2.5 using CHARMm force field adjusting the ionization pH at 7.4, with no isomers or tautomers generated for the ligands. Figures were generated using UCSF Chimera^32^ and Discovery Studio 2.5. Physicochemical and drug likeness properties are predicted using SwissADME webserver (https://www.nature.com/articles/srep42717).

## Results and discussion

### Chemistry

The titled compound (**5**) was prepared in good yield and purity via nucleophilic substitution of the 2-aminobenzothiazole derivatives (**4**) on 2-naphthylsulfonyl chloride in dry pyridine.

Compound (**6**) was obtained through ester hydrolysis using LiOH.H_2_O in ethanol/water (1:1)^33^.

**Figure 3:**
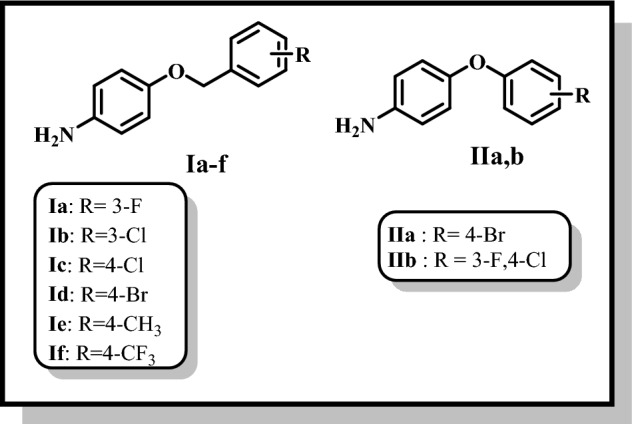
Structures of reported intermediates **Ia-f** and **IIa,b** used in the synthesis of the final compounds.

**Figure 4:**
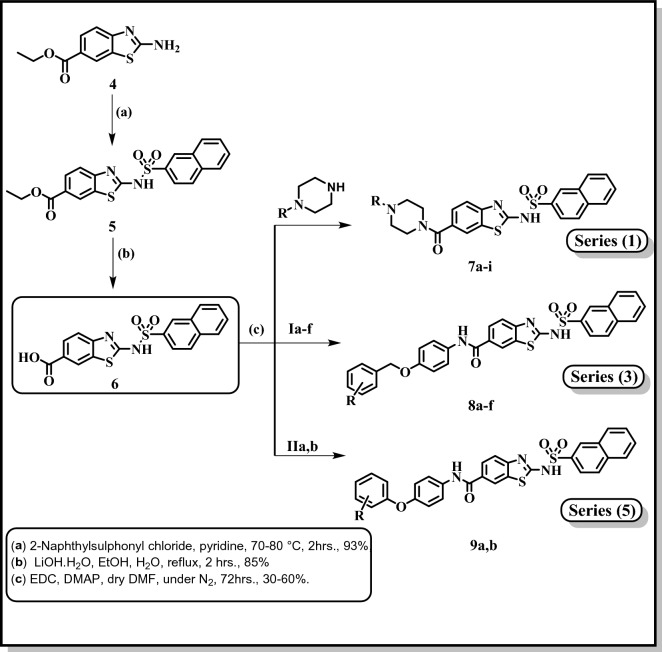
Synthesis of final compounds **7a-i** (Series 1), **8a-f** (Series 3) and **9a,b** (Series 5) **7a**: R = Phenyl, **7b**: R = 2-Fluorophenyl, **7c**: R = 2-Methoxyphenyl, **7d**: R = 3,4 Dichlorophenyl, **7e**: R = 4-Chlorophenyl, **7f**: R = Benzhydryl, **7g**: R = Trans-Cinammyl, **7h**: R = 1-Piperonyl, **7i**: R = 2-Tetrahydrofuroyl, **8a**: R = 3-F, **8b**: R = 3-Cl, **8c**: R = 4-Cl, **8d**: R = 4-Br, **8e**: R = 4- CH_3_, **8f**: R = 4-CF_3_, **9a**: R = 3-F,4-Cl, **9b**: R = 4-Br.

Compound **6** served as a crucial starting point for the synthesis of series 1, 3 and 5 (scheme 2). the titled compounds, **series 1**, (**7a-i**) were prepared by direct coupling of equimolar amounts of the carboxylic acid (**6**) and the respective substituted piperazine in presence of EDC.HCl as coupling agent/ DMAP as a base in dry DMF under N_2_ atmosphere^34^. **Series 3**, compounds (**8a-f**), was prepared though a similar pattern as compounds **7a-i**, by the direct coupling of equimolar amounts of the carboxylic acid (**10**) and the respective amino compounds (**Ia-f**, Fig. 3) using EDC.HCl as coupling agent/ DMAP as a base in dry DMF under N_2_ atmosphere^34^. Using the same coupling procedure^34^, compound (**6**) and the respective amino compounds (**IIa,b**, Fig. 3) were reacted yielding **9a,b, series 5**, Fig. 4.

**Figure 5:**
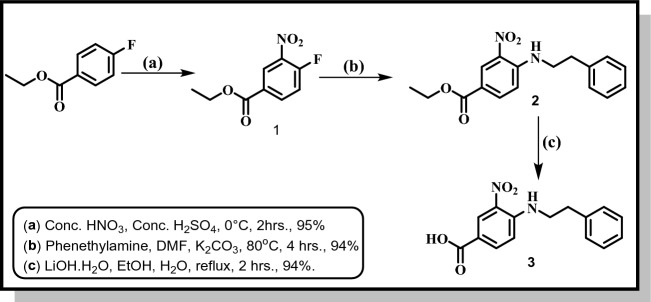
Synthesis of 3-Nitro-4-(phenethylamino) benzoic acid **(3)**.

Furthermore, we applied the same above-mentioned coupling procedure to obtain the titled compound (**10**). Compound (**10)** was prepared upon reaction of 3-nitro-4-(phenethylamino)benzoic acid (**3**), which was obtained in good yields (94%) and purity according to Fig. 5, and ethyl 2-amino benzo[*d*]thiazole-6-carboxylate (**4**)^34^. The titled compound (**10**) was subjected to ester hydrolysis to yield compound (**11**).

**Figure 6:**
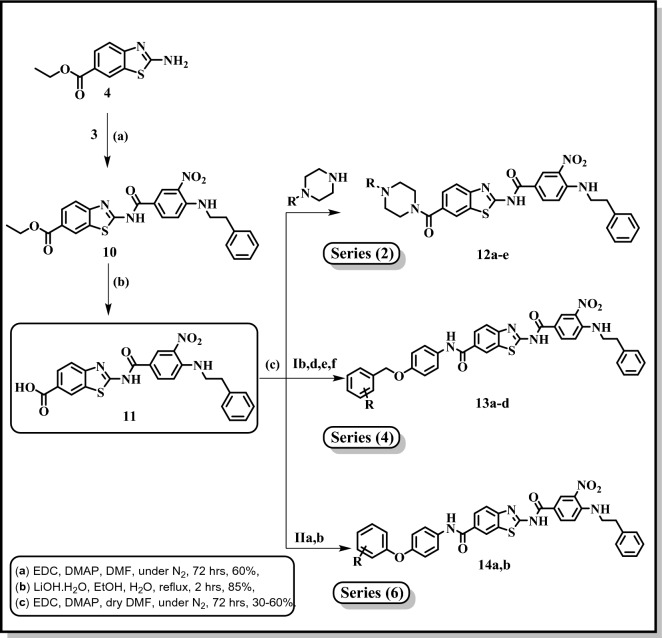
Synthesis of final compounds: **12a-e** (Series 2), **13a-d** (Series 4) and **14a,b** (Series 6); **12a**: R = 2-Methoxyphenyl, **12b**: R = 3,4-Dichlorophenyl, **12c**: R = Benzhydryl, **12d**: R = Trans-Cinammyl, **12e**: R = 2-Tetrahydrofuroyl; **13a**: R = 3-Cl, **13b**: R = 4-Br, **13c**: R = 4-CH_3_, **13d**: R = 4-CF_3_; **14a**: R = 3-F,4-Cl, **14b**: R = 4-Br.

According to Fig. 6, compound **11** is considered very important starting building block for the synthesis of **series 2, 4** and **6**. Compound lists **12a-e, 13a-d** and **14a,b** were obtained using the same coupling procedure using compound **11** and respective substituted piperazines, amino compounds (**Ia-f**) and (**IIa,b**) respectively.

**Figure 7:**
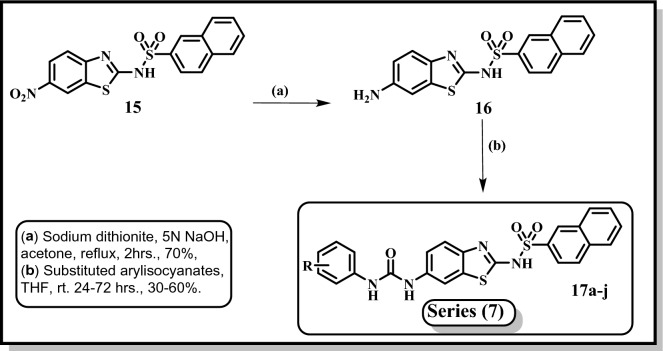
Synthesis of final compounds **17a-j** (Series 7); **17a**: R = H, **17b**: R = 3- Cl, **17c**: R = 3- Br, **17d**: R = 3- O-CH_3_, **17e**: R = 3-CH_3_, **17f**: R = 4- Br, **17g**: R = 4- Cl, 3-CF3, **17h**: R = 3,4- di Cl, **17i**: R = 2-CH_3_,4-Cl, **17j**: R = 3-Cl,4-CH_3_.

Finally, Fig. 7 illustrates the synthesis of the final compounds **17a-j**, **series 7**. Compound **15** was synthesized according to the reported method where it was produced in good yield and purity^35^. Furthermore, nitro group in compound **15** was reduced following the reported procedure^35^. Compound **16** served as crucial starting material to obtain compounds **17a-j**, where it reacted with the respective isocyanate in THF^36^. The urea derivatives **17a-j** were obtained in good yield and purity. The structures of all the newly synthesized compounds were confirmed by their spectral data.

### Biological evaluation

#### Initial in vitro BCL-2 inhibitory screening at single dose of 10 µM concentration

**Figure 8:**
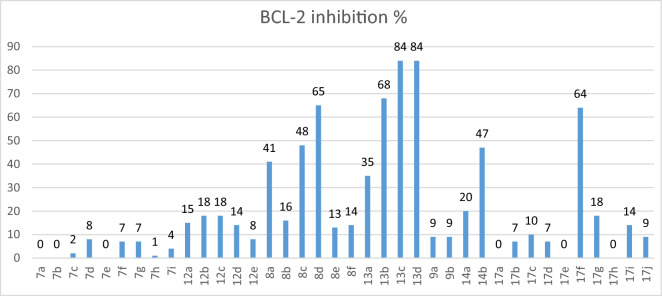
Percent inhibition of BCL-2 inhibitory activity achieved by the newly synthesized compounds.

Our novel synthesized compounds were screened for their potential BCL-2 anti-apoptotic effects. The in vitro BCL-2 inhibition assay was executed at the BPS Bioscience Corporation, San Diego, CA, USA. Table 1 and Fig. 8 illustrate the inhibitory effects of the compounds against BCL-2 protein, which happened to be either very poor or good inhibitory activity.

**Table 1:** Percent inhibition of BCL-2 inhibitory activity achieved by the piperazine derivatives, **7a-i (Series 1), 12a-e (Series 2),** benzyloxy derivatives **series 3 (8a-f), series 4 (13a-d), 13a,b (Series 5), 14a,b (Series 6),** and **17a-j (Series 7)** at 10 µM.

ID	BCL-2 inhibition %	ID	BCL-2 inhibition %
**7a**	0	**12c**	18
**7b**	0	**12d**	14
**7c**	2	**12e**	8
**7d**	8	**13a**	35
**7e**	0	**13b**	68
**7f.**	7	**13c**	84
**7 g**	7	**13d**	84
**7 h**	1	**14a**	20
**7i**	4	**14b**	47
**8a**	41	**17a**	0
**8b**	16	**17b**	7
**8c**	48	**17c**	10
**8d**	65	**17d**	7
**8e**	13	**17e**	0
**8f**	14	**17f**	64
**9a**	9	**17g**	18
**9b**	9	**17h**	0
**12a**	15	**17i**	14
**12b**	18	**17j**	9
**ABT-199 (20)**	92 (at 100 nM)

**Figure 9:**
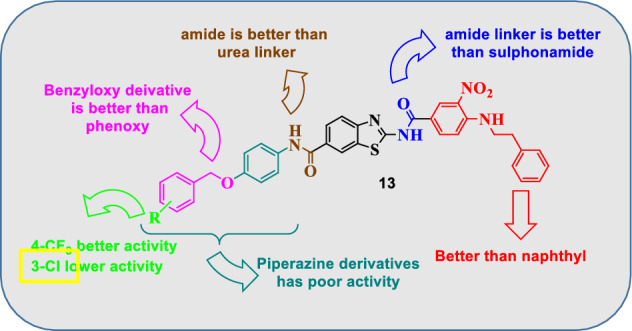
Structure activity relationship diagram showing the most potent compound **13**.

Regarding the P4 hydrophobic binding moiety, where incorporation of 3-nitro-4-(phenethylamino)benzamide moiety at position 2 of benzothiazole scaffold generally resulted in marked improvement of BCL-2 inhibitory activity when compared to their naphthyl sulfonamide analogues (Series 2 ,4 & 6 compared to series 1, 3 & 5). Regarding the P2 hydrophobic binding moiety, para substituted benzyloxyphenyl derivatives (series 3 & 4), compounds (**8c,d** and **13b-d**), exhibited better BCL-2 inhibitory activity than the corresponding phenoxyphenyl derivatives (**13** and **14**). These compounds showed good inhibitory activity with BCL-2 percent inhibition of 48, 65, 68, 84 and 84%, respectively. Moreover, the 3-fluorobenzyloxy derivative (**8a**) and 3-chlorobenzyloxy derivative (**13a**) also exhibited moderate inhibitory activity with BCL-2 percent inhibition of 41 and 35%, respectively. As mentioned above, compounds bearing 3-nitro-4-phenylaminobenzamide tail were superior to their naphthyl sulfonamide analogues (compounds **(13a**-**d)** versus compounds (**8c-f**). Regarding the phenoxyphenyl derivatives (**series 5 & 6**), only the 4-bromo phenoxyphenyl derivative (**14b**) showed moderate BCL-2 inhibitory activity with 47% inhibition. Investigating the urea derived compounds (**series 7**), also revealed that the 4-bromo phenyl urea derivative (**17f**) exhibited good BCL-2 inhibitory activity with 64% inhibition. On the contrary, the piperazine derivatives **(series 1 & 2)** showed no or poor inhibitory activity on BCL-2 with **18%** as the most achieved inhibition by compounds (**12b** and **12c**), Fig. 9.

#### Measurement of potential enzyme inhibitory activity (IC_50_)

Promising candidates, which exhibited BCL-2 inhibition percent above 60% at 10 µM concentration (**8d**, **13b-d** & **17f**) were further investigated for their dose-related BCL-2 inhibitory activity at 5 different concentrations (1 nM—10 nM—100 nM—1 µM—10 µM) to subsequently calculate their IC_50_ values, Table 2.

**Table 2:** The IC_50_ values for compounds **8d**, **13b-d** and **17f**.

ID	BCL-2% inhibition	BCL-2 IC_50_
**8d**	65	1.620 µM
**13b**	68	1.087 µM
**13c**	84	**0.471 µM**
**13d**	84	**0.363 µM**
**17f**	64	2.290 µM
**ABT-199 (20)**	92 (at 100 nM)	0.007 µM

Significant values are in bold.

#### In vitro anti-proliferative activity against leukemia NCI cell lines

This assay was performed for compounds **13c** and **13d** by the Developmental Therapeutics Program (DTP) of the National Cancer Institute (NCI), division of cancer treatment and diagnosis, NIH, Bethesda, Maryland, USA (www.dtp.nci.nih.gov). The operation of this screen utilizes leukemia human tumor cell line^37^. The human tumor cell lines of the cancer screening panel were grown in RPMI 1640 medium containing 5% fetal bovine serum and 2 mM L-glutamine.

Results for the two compounds were reported as a mean graph of the percent growth of the treated cells when compared to the untreated control cells. The results are expressed as cell growth percent for the tested compounds on each of the used NCI cell line panels. Table 3 shows the results for the compounds **13c** and **13d**.

**Table 3:** Cell growth percentage of NCI leukemia cancer cell line exhibited by the final compounds **13c** and **13d**.

Cell growth percent for the tested compounds		
Leukemia		
Panel/cell line	**13c**	**13d**
**CCRF-CEM**	93.91	**68.2**
**HL-60(TB)**	75.74	96.87
**K-562**	87.23	**67.92**
**MOLT-4**	94.74	74.37
**RPMI-8226**	87.5	**69.86**
**SR**	82.5	**37.13**

Significant values are in bold.

Compound **13d**, which demonstrated the highest inhibitory activity against BCL-2 with IC_50_ value of **0.363 µM**, exhibited moderate inhibition against leukemic SR cell lines with growth inhibition **62.87**. While compound **13c** exhibited poor inhibition against the used leukemia cell line.

### Molecular modelling study

**Figure 10:**
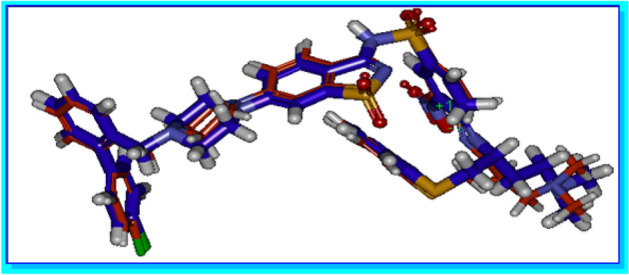
The alignment between the co-crystallized bioactive conformer of compound **(blue**) and the pose of the same compound retrieved from docking using CDOCKER (**orange**). Figure 10 was generated using Discovery studio 2.5.

The validity of the proposed design was evaluated by molecular docking study through predicting the binding modes and binding affinities of the designed compounds. These predicted properties should meet the minimum desirable requirements to validate the hypothesis that the designed compounds have proposed biological activities against BCL-2. The docking study was performed in order to test for a comparable binding mode to the saccharine lead compound **2** and investigate the binding affinities of the designed compounds to BCL-2 binding groove. The major goal of a good docking protocol is to discriminate between the groups of true solutions (proposed poses), usually defined as poses within 2.0 Å root mean square deviations (RMSD) from the X-ray geometry, and false solutions or misdocked structures^38^. In order to validate the C-DOCKER protocol's predictability of the correct poses, we re-docked the co-crystallized ligand using C-DOCKER, and aligned the pose retrieved from docking to the X-ray geometry (The co-crystallized bioactive conformation) to calculate the RMSD. The docking of this compound in BCL-2 binding groove generated RMSD of 0.6656 Å (Fig. 10), therefore, the C-DOCKER protocol is reliable for predicting the poses of the tested compounds in the X-ray crystal structure of BCL-2 protein.

The docking study of the synthesized compounds into BCL-2 binding groove revealed comparable binding modes of the docked molecules to the lead compound. Docking of the target compounds showed that the core scaffold adopted volumes and orientation as the lead compound. The docking results showed the designed compounds to have C-Docker interaction energies ranging from − 67.57 to − 40.31 kcal/mole which came comparable to the docking score of the co-crystallized ligand with docking score of − 71.89 kcal/mol. The docking score of the 5 most active compounds come in a good agreement with their IC_50_, where they are ranked in the same order according to the IC_50_ order. They are arranged from the most active to the least active as follows: 13d > 13c > 13b > 8d > 17f, as shown in Table 4.

**Table 4:** Docking scores, 2D interaction diagrams, and interacting amino acids with compounds **8d**, **13b-d** and **17f**.

ID	2D interaction diagram	C-docker interaction energy & binding interactions
**8d**		** − 53.08**
		**HBA** Arg 66
		**HBA** Arg 105
		**Pi-Pi** Tyr 67
		**Pi-Pi** Tyr 161
**13b**		** − 55.83**
		**HBA** Arg 66
		**HBA** Arg 105
		**Pi-Pi** Tyr 161
**13c**		** − 58.05**
		**HBA** Gly 104
		**Pi-Alkyl** Ala 59
		**Pi-Alkyl** Arg 98
		**Pi-Alkyl** Val 107
		**Pi-Alkyl** Ala 108
		**Pi-Alkyl** Pro 163
**13d**		** − 59.15**
		**HBA** Gly 104
		**Pi-Pi** Tyr 67
		**Pi-Pi** Tyr 161
		**Pi- Pi-Alkyl** Ala 59
		**Pi-Alkyl** Val 92
		**Pi-Alkyl** Pro 163
**17f.**		** − 41.69**
		**HBA** Arg 66
		**HBA** Arg 105
		**Pi-Pi** Tyr 161
		**Pi- Sulfur** Tyr 67
		**Pi- Alkyl** Arg 98
		**Pi- Alkyl** Ala 108

**Figure 11:**
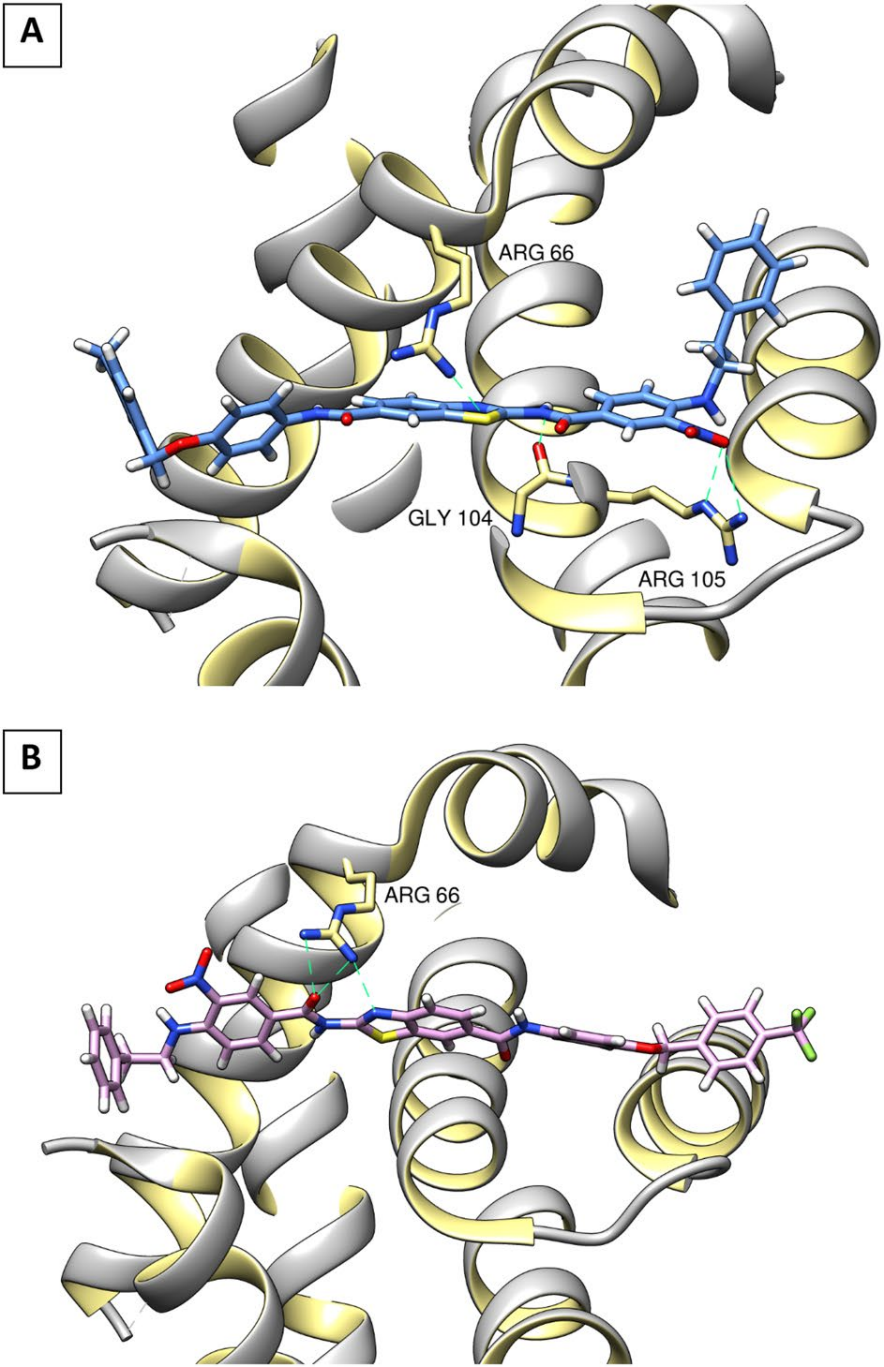
3D interaction diagram of the most active compounds **13c** (**A**) and **13d **(**B**) with BCL-2 BH3 binding groove. Figure 11 was generated using Chimera 1.16 (https://www.cgl.ucsf.edu/chimera/).

*P*-substituted-benzyloxyphenyl derivatives **(series 3 & 4)**, exhibited the best BCL-2 inhibitory activity as they fulfilled most of the key interactions with BCL-2, where the key hydrogen bonds with Gly 104 and Arg 66 residues were established (as seen in compounds **13c** & **13d**, which are the most active compounds against BCL-2), as well as a pi–pi interaction with Tyr67 and Tyr161. Also, pi-alky interactions were observed with various amino acid residues (Arg 98, Ala 59 & Pro163) resulting in better docking scores as well, which could explain the good BCL-2 inhibitory activity of those compounds (Fig. 11A and B).

**Figure 12:**
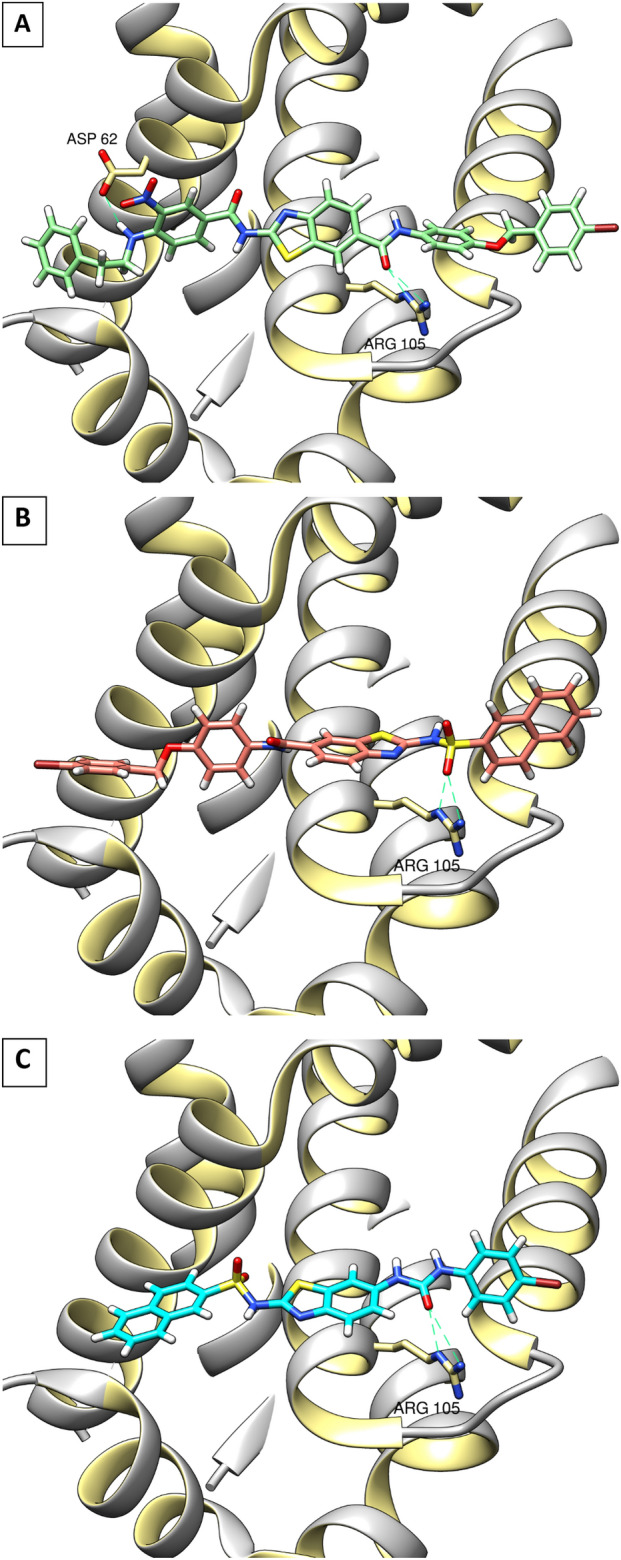
3D interaction diagram of the compounds **13b** (**A**), **8d** (**B**) & **17f** (**C**) with BCL-2 BH3 binding groove. Figure 12 was generated using Chimera 1.16 (https://www.cgl.ucsf.edu/chimera/).

Regarding the moderately active compounds **(8a,c,d, 13b, 14b & 17f)**, they showed the same binding mode and interactions as the most active compounds **(13c & 13d)** but they missed a key HB with Gly 104 which could explain its moderate activity (Fig. 12). However, they formed another H-bond with Arg 105 residue compensating the loss of the main HB with Gly 104 (Table S1).

On the contrary, piperazine derivatives (**series 1 & 2**) failed to exhibit BCL-2 inhibitory activity as they missed the key interaction with Gly104 residue which seemed to be an essential feature for BCL-2 inhibitory activity, although they maintained the same binding mode and the same key hydrogen bond with Arg 66 and hydrophobic interactions (Pi-Pi) with Tyr 67, Tyr 161. Generally, most of inactive compounds missed this key hydrogen bond interaction with Gly 104 residue. Moreover, they showed lower docking scores relative to that of the docked lead compound. 2D interactions, docking scores, and detailed binding interactions for active compounds with BCL-2 inhibition > 50% are reported in Table S1 in supporting info.

#### Physicochemical and drug likeness properties

**Figure 13:**
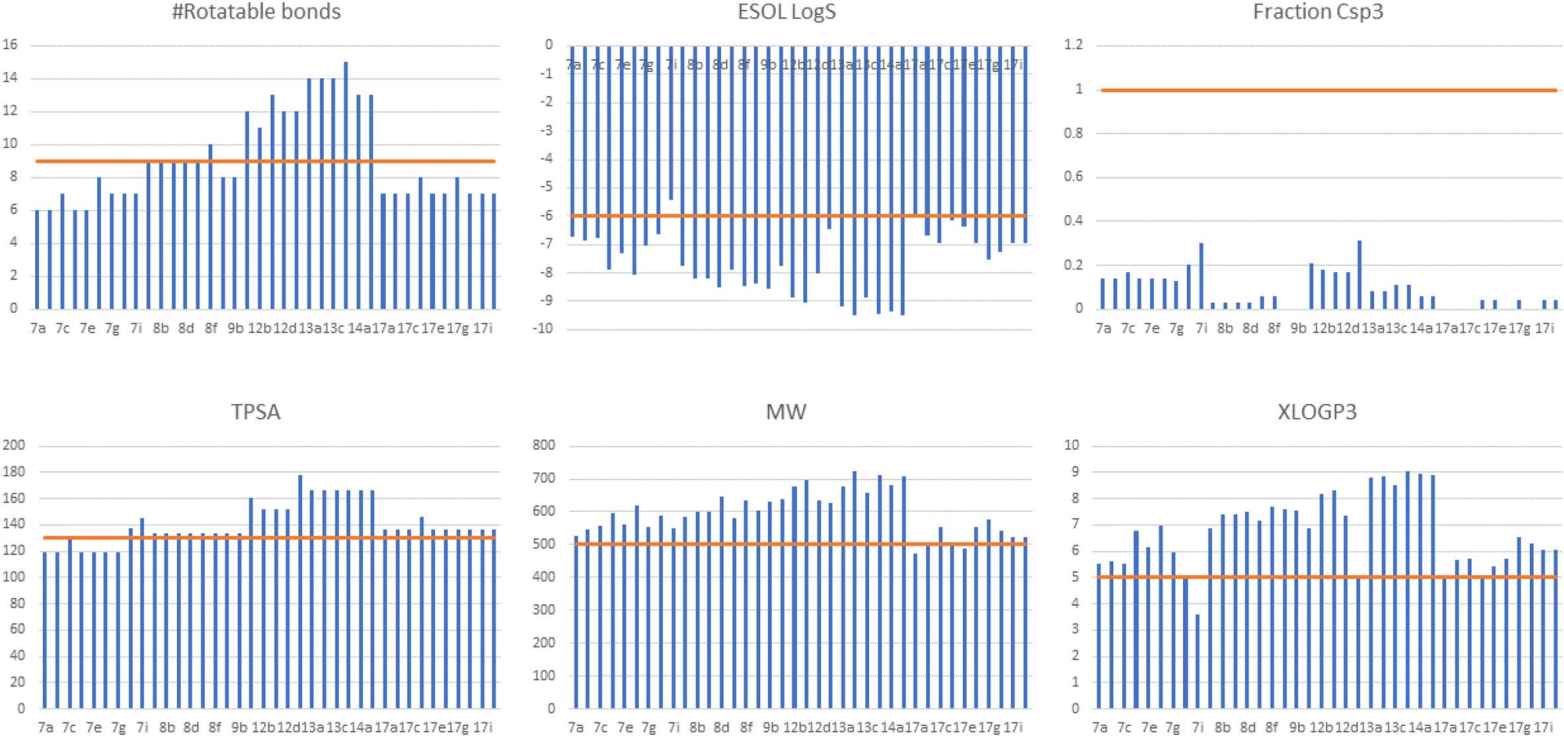
Six physicochemical properties that determine the bioavailability, number of rotatable bonds, solubility ESOL Log S, sp3 carbon fraction, topological polar surface area (TPSA), molecular weight (MW), lipophilicity (XLOGP3). That upper/lower cutoff for each property is drawn in orange horizontal line.

The *insilico* physicochemical properties of the reported compounds were calculated using SwissADME web server. Six parameters that detect the oral bioavailability were calculated namely number of rotatable bonds, Solubility Log S, fraction of sp3 carbons, Topological polar surface area (TPSA), molecular weight (MW), and XLOGP3 for lipophilicity, as shown in Fig. 13. Most of the compounds showed acceptable number of rotatable bonds (< 9), TPSA (< 130 Å^2^), and sp3 carbon fraction (< 1). Most of the compounds showed moderate water solubility (ESOL Log S < -6), slightly high molecular weight (> 500), and high lipophilicity (XLOGP3 > 5),. These results gave a basis for further drug development for the reported series to adjust the lipophilic/hydrophilic balance, solubility, and molecular weight.

The drug likeness for the reported compounds was also predicted using SwissADME web server by calculating five filters namely, Lipinski, Ghose, Veber, Egan, and Muegge. The results were reported by the number of violations each compound commits per filter in Table 5. The results are depicted as a heat map. Most compounds fulfil all the filters except for the Ghose filter which come from higher molecular weight and molar refractivity. This also can be exploited in prospective drug development projects by adjusting the molecular weight of the designed compounds.

**Table 5:** The predicted Drug likeness for the synthesized compounds using SwissADME web server.

ID	Lipinski	Ghose	Veber	Egan	Muegge
**7a**	1	2	0	0	1
**7b**	1	3	0	1	1
**7c**	1	2	0	0	1
**7d**	2	3	0	1	1
**7e**	1	3	0	1	1
**7f**	2	4	0	1	2
**7g**	1	3	0	0	1
**7h**	1	2	0	1	1
**7i**	1	2	1	1	0
**8a**	2	3	0	2	1
**8b**	2	3	0	2	2
**8c**	2	3	0	2	2
**8d**	2	3	0	2	2
**8e**	2	3	0	2	1
**8f**	2	3	0	2	2
**9a**	2	3	0	2	2
**9b**	2	3	0	2	2
**12a**	2	3	2	1	3
**12b**	1	4	2	2	3
**12c**	1	4	2	2	3
**12d**	1	3	2	1	3
**12e**	2	3	2	1	2
**13a**	2	4	2	2	3
**13b**	2	4	2	2	3
**13c**	1	4	2	2	3
**13d**	2	4	2	2	3
**14a**	2	4	2	2	3
**14b**	2	4	2	2	3
**17a**	0	2	0	2	1
**17b**	1	3	0	2	1
**17c**	1	3	0	2	1
**17d**	1	3	1	2	1
**17e**	0	3	0	2	1
**17f**	1	3	0	2	1
**17g**	2	3	0	2	1
**17h**	2	3	0	2	1
**17i**	1	3	0	2	1
**17j**	1	3	0	2	1

## Conclusion

Our main approach was to design new small molecules as BCL-2 inhibitors with potential anti-cancer activity based on literature review and SAR studies, novel benzothiazole-based compounds were designed, synthesized and evaluated for their in vitro BCL-2 inhibitory activity. The 4-CH_3_ and 4-CF_3_ benzyloxy derivatives (**13c** & **13d**) having 3-nitro-4-phenethyl amino moiety as the P4 hydrophobic binding group, exhibited the highest inhibitory activity against BCL-2 protein with IC_50_ values of 0.471 and 0.363 µM, respectively. The designed and synthesized compounds could act as a potential cornerstone for future design of more potent and selective BCL-2 inhibitors and hence promising anticancer agents.

### Supplementary Information

Supplementary Information.

## Data Availability

All data generated or analysed for this study are included in this published paper (and its Supplementary Information files).

## References

[1] Avendaño C, Menéndez JC (2015). Medicinal Chemistry of Anticancer Drugs.

[2] Hanahan D, Weinberg RA (2011). Hallmarks of cancer: The next generation. Cell.

[3] Renehan AG, Booth C, Potten CS (2001). What is apoptosis, and why is it important?. BMJ.

[4] Soliman AM (2021). Induction of apoptosis, cytotoxicity and radiosensitization by novel 3,4-dihydroquinazolinone derivatives. Bioorg. Med. Chem. Lett..

[5] Wong RSY (2011). Apoptosis in cancer: From pathogenesis to treatment. J. Exp. Clin. Cancer Res..

[6] Kasibhatla S, Tseng B (2003). Why target apoptosis in cancer treatment?. Mol. Cancer Ther..

[7] Boice A, Bouchier-Hayes L (2020). Targeting apoptotic caspases in cancer. Biochim. Biophys. Acta Mol. Cell Res..

[8] Van Opdenbosch N, Lamkanfi M (2019). Caspases in cell death, inflammation, and disease. Immunity.

[9] Garrido C (2006). Mechanisms of cytochrome c release from mitochondria. Cell Death Differ..

[10] Pfeffer CM, Singh ATK (2018). Apoptosis: A target for anticancer therapy. Int. J. Mol. Sci..

[11] Reed JC, Pellecchia M (2005). Apoptosis-based therapies for hematologic malignancies. Blood.

[12] Adams JM, Cory S (2007). The Bcl-2 apoptotic switch in cancer development and therapy. Oncogene.

[13] Kale J, Osterlund EJ, Andrews DW (2018). BCL-2 family proteins: Changing partners in the dance towards death. Cell Death Differ..

[14] Warren CFA, Wong-Brown MW, Bowden NA (2019). BCL-2 family isoforms in apoptosis and cancer. Cell Death Dis..

[15] Kroemer G, Galluzzi L, Brenner C (2007). Mitochondrial membrane permeabilization in cell death. Physiol. Rev..

[16] Hata AN, Engelman JA, Faber AC (2015). The BCL2 family: Key mediators of the apoptotic response to targeted anticancer therapeutics. Cancer Discov..

[17] Kvansakul M, Hinds MG (2015). The Bcl-2 family: Structures, interactions and targets for drug discovery. Apoptosis.

[18] Chan SL, Yu VC (2004). Proteins of the bcl-2 family in apoptosis signalling: From mechanistic insights to therapeutic opportunities. Clin. Exp. Pharmacol. Physiol..

[19] Youle RJ, Strasser A (2008). The BCL-2 protein family: Opposing activities that mediate cell death. Nat. Rev. Mol. Cell Biol..

[20] Obeng E (2021). Apoptosis (programmed cell death) and its signals—A review. Braz J Biol.

[21] Shimizu S (2004). Role of Bcl-2 family proteins in a non-apoptotic programmed cell death dependent on autophagy genes. Nat. Cell Biol..

[22] Reed JS (2011). The role of MHC class I allele Mamu-A*07 during SIV(mac)239 infection. Immunogenetics.

[23] Khan KH, Blanco-Codesido M, Molife LR (2014). Cancer therapeutics: Targeting the apoptotic pathway. Crit. Rev. Oncol. Hematol..

[24] Kapoor I (2020). Targeting BCL-2 in B-cell malignancies and overcoming therapeutic resistance. Cell Death Dis..

[25] Ishikawa M, Hashimoto Y (2011). Improvement in aqueous solubility in small molecule drug discovery programs by disruption of molecular planarity and symmetry. J. Med. Chem..

[26] Sleebs BE (2011). Quinazoline sulfonamides as dual binders of the proteins B-cell lymphoma 2 and B-cell lymphoma extra long with potent proapoptotic cell-based activity. J. Med. Chem..

[27] Perez HL (2012). Identification of a phenylacylsulfonamide series of dual Bcl-2/Bcl-xL antagonists. Bioorg. Med. Chem. Lett..

[28] Schroeder GM (2012). Pyrazole and pyrimidine phenylacylsulfonamides as dual Bcl-2/Bcl-xL antagonists. Bioorg. Med. Chem. Lett..

[29] Touré BB (2013). The role of the acidity of N-heteroaryl sulfonamides as inhibitors of bcl-2 family protein-protein interactions. ACS Med. Chem. Lett..

[30] Filippakopoulos P (2012). Histone recognition and large-scale structural analysis of the human bromodomain family. Cell.

[31] Berman HM (2000). The protein data bank. Nucleic Acids Res..

[32] Pettersen EF (2004). UCSF Chimera–a visualization system for exploratory research and analysis. J. Comput. Chem..

[33] Gibson CL (2009). Diversity oriented syntheses of fused pyrimidines designed as potential antifolates. Org. Biomol. Chem..

[34] Leow ML (2013). Benzofuran-based estrogen receptor α modulators as anti-cancer therapeutics: In silico and experimental studies. Curr. Med. Chem..

[35] González-Alvarez M (2003). Development of novel copper(II) complexes of benzothiazole- N-sulfonamides as protective agents against superoxide anion. Crystal structures of [Cu( N-2-(4-methylbenzothiazole)benzenesulfonamidate)(2)(py)(2)] and [Cu( N-2-(6-nitrobenzothiazole)naphthalenesulfonamidate)(2)(py)(2)]. J. Biol. Inorg. Chem..

[36] Manoharan D (2017). Synthesis, characterization and evaluation of antidiabetic activity of novel indoline derivatives. Bangladesh J. Pharmacol..

[37] Shoemaker RH (2006). The NCI60 human tumour cell line anticancer drug screen. Nat. Rev. Cancer.

[38] Wu G (2003). Detailed analysis of grid-based molecular docking: A case study of CDOCKER-A CHARMm-based MD docking algorithm. J. Comput. Chem..

