# Supplementary material for: Design, molecular modelling and synthesis of novel benzothiazole derivatives as BCL-2 inhibitors
Source: Sci Rep. 2023 Sep 20;13:15554. doi: 10.1038/s41598-023-41783-1 (PMC10511702; doi:10.1038/s41598-023-41783-1)
Supplement: Supplementary file 1 — Supplementary Information. [file 41598_2023_41783_MOESM1_ESM.docx]

# Supporting Information for:

**Design, Molecular Modelling and Synthesis of Novel Benzothiazole Derivatives as BCL-2 inhibitors**

**Hoda S. Ismail, Amira Khalil, Rabah A. Taha, Deena S. Lasheen**

**Dalal A. Abou El Ella**

1. **Synthetic Procedure of key intermediates……………………………………………………………………………………………………………..…2**
2. **Synthetic procedures and structural characterization of the synthesized compounds……………………………………………..14**
3. **Docking poses and energies of key products…………………………………………………………………………………………………………..35**
4. **Spectral Data of final compounds……………………………………………………………………………………………………………………..……39**
5. **NCI assay protocol………………………………………………………………………………………………………………………………………………….76**
6. **References ……………………………………………………………………………………………………………………………………………………..…….76**

# Synthetic Procedure of key intermediates

**Ethyl 4-fluoro-3-nitrobenzoate (1):[1]**

Conc. HNO_3_ (8 mL) was added dropwise to a mixture of ethyl 4-fluorobenzoate (8.94 g; 53.7 mmol) in conc. H_2_SO_4_ (8 mL) at 0 °C. After stirring for 2 hours at 0 °C, the mixture was poured onto ice and extracted with EtOAc (3 X 50 mL). The organic layers were combined, washed with saturated NaHCO_3_ solution (100 ml), dried over anhydrous Na_2_SO_4_ and concentrated under reduced pressure to give ethyl 4-fluoro-3-nitrobenzoate **(1)** as a pale yellow solid with a yield of (10.77g, 95%); m.p. 42-44 **°**C as reported [1].

**Ethyl 3-nitro-4-(phenethylamino)benzoate (2):**

Phenethylamine (2.3 mL; 17.8 mmol) was added to a stirred solution of ethyl 4-fluoro-3-nitrobenzoate **(1)** (3.60 g, 16.9 mmol) and K_2_CO_3_ (4.67 g, 33.8 mmol) in 50 mL DMF. After stirring for 4 hours at the mixture was poured onto ice water, stirred for 10 minutes, filtered and dried to give ethyl 3-nitro-4-(phenethylamino)benzoate **(2)** as yellow solid (4.99 g ,94%); m.p. 92-94**°**C; **(**M.wt.: 314.34, C_17_H_18_N_2_O_4_); **^1^H-NMR (400 MHz, DMSO-*d_6_*)** δ 10.3 (s, 1H, NH D_2_O exchangeable), 8.71 (1H, s, Ar-H), 8.25 (1H, d, *J* = 8.4 Hz, Ar-H), 7.28 (2H, d, *J* =7.8 Hz, Ar-H), 7.2 (1H, t, *J*= 7.7 Hz, Ar-H), 7.09 (2H, d, *J*= 7.8, Ar-H), 6.82 (1H, d, *J* = 8.4 Hz, Ar-H), 4 .45 (2H, q, *J* = 7.1 Hz, CH_3_CH_2_O-), 3.88 (2H, t, *J* = 5.7 Hz, -N-CH_2_-CH_2_-), 3.28 (2H, t, *J* = 5.7 Hz, -CH_2_-Ar), 1.44 (3H, t, *J* = 7.2 Hz, CH_3_CH_2_O-).

**3-Nitro-4-(phenethylamino)benzoic acid (3):**

Ethyl 3-nitro-4-(phenethylamino)benzoate **(2)** (2.16 g, 6.9 mmol) was added to a solution of LiOH.H_2_O (0.579 g, 13.8 mmol) in ethanol (50%, 70mL).The mixture was heated under reflux for 2 hours. The resulting solution was allowed to cool to room temperature then added to 10% HCl/ice (100 mL) with continued stirring. The resulting solid was filtered, washed with water and recrystallized from THF/water to afford 3-nitro-4-(phenethylamino)benzoic acid **(3)** as yellow solid (1.86 g ,94%); m.p. 198-200**°**C; **(**M.wt.: 286.28, C_15_H_14_N_2_O_4_); **^1^H NMR (400 MHz, DMSO-*d_6_*)** δ 12.20 (s, 1H, COOH, D_2_O exchangeable), 10.3 (s, 1H, NH D_2_O exchangeable), 8.69 (1H, s, Ar-H), 7.43 (1H, d, J = 8.4 Hz, Ar-H), 7.28 (2H, d, J =7.8 Hz, Ar-H), 7.2 (1H, t, J= 7.7 Hz, Ar-H), 7.09 (2H, d, J= 7.8, Ar-H), 6.92 (1H, d, J = 8.4 Hz, Ar-H) 3.88 (2H, t, J = 5.7 Hz, -N-CH_2_-CH_2_-), 3.18 (2H, t, J = 5.7 Hz, -CH_2_-Ar).

### 5.1.2.1.2. Supplementary figure 1:

**1-(Substituted-benzyloxy)-4-nitrobenzene (4a-f) [2, 3]:**

**General Procedure:**

A mixture of 4-nitrophenol (0.4 g, 2.88 mmol), the respective substituted benzyl bromide (*viz*; 3-fluorobenzyl bromide, 3-chlorobenzyl bromide, 4-chlorobenzyl bromide, 4-bromobenzyl bromide, 4-methylbenzyl bromide and 4-trifluoromethyl benzyl bromide) (2.88 mmol) and anhydrous K_2_CO_3_ (0.66 g, 4.78 mmol) in dry DMF (10 mL) was heated at 80 ^o^C for 24 hours. After cooling, the reaction mixture was poured into ice/water (100 mL). The precipitated solid was filtered, washed with water, and then recrystallized from ethanol/water to afford the titled compounds **(4a-f)** [2, 3].

**1-((3-Fluorobenzyl)oxy)-4-nitrobenzene** **(4a):**

The titled compound was obtained as yellow solid (0.69g, 97%); m.p. 104-105°C (as reported).

**1-((3-Chlorobenzyl)oxy)-4-nitrobenzene (4b):[2]**

The titled compound was obtained as off-white solid (0.74g, 98%); m.p. 98-100°C (as reported).[2]

**1-((4-Chlorobenzyl)oxy)-4-nitrobenzene (4c)**:[4]

The titled compound was obtained as white solid (0.73g, 97%); m.p. 114-115°C (as reported).[4]

**1-((4-Bromobenzyl)oxy)-4-nitrobenzene (4d)**:[5]

The titled compound was obtained as off-white solid (0.84g, 95%); m.p. 116-118°C (as reported).[5]

**1-((4-Methylbenzyl)oxy)-4-nitrobenzene (4e)** [3]

The titled compound was obtained as off-white solid (0.69g, 99%); m.p. 108-109°C (as reported).[3]

**1-((4-Trifluoromethylbenzyl)oxy)-4-nitrobenzene (4f)**:

The titled compound was obtained as off-white solid (0.86g, 100%); m.p. 74-76°C; (M.wt.= 297.23, C_14_H_10_F_3_NO_3_); **^1^H NMR (400 MHz, DMSO-*d*_6_)**  δ 7.43(d, J = 8.3 Hz, 2H, Ar-H), 7.28 (d, J = 8.3 Hz, 2H, Ar-H), 6.77 (d, J = 6.6 Hz, 2H, Ar–H), 6.62 (d, 𝐽 = 6.6 Hz, 2H, Ar–H), 5.22 (s, 2H, O-CH_2_).

**4-((Substituted-benzyl)oxy)aniline (5a-f)**:[6]

**General Procedure:**

A mixture of the relevant nitro derivative **(4a-f)** (3 mmol), reduced iron powder (0.5 g, 9 mmol) and NH_4_Cl (1.44 g, 27 mmol) in 70% EtOH/H_2_O (20 mL) was heated at reflux temperature (70 ^o^C) for 2 hours. The reaction mixture was filtered over a Celite pad to remove the insoluble iron oxides. The filtrate was evaporated under vacuum, EtOAc (50 mL) was added to the residue, and then the resulting suspension was filtered to remove the inorganic salts. The filtrate was dried over anhydrous Na_2_SO_4_ and evaporated under vacuum to afford the crystals of the titled compounds **(5a-f)** which were recrystallized from ethyl acetate/petroleum ether.

**4-((3-Fluorobenzyl)oxy)aniline (5a)**:

The titled compound was obtained as brown crystals (0.65g, 85%); m.p. 84-86°C (as reported).

**4-((3-Chlorobenzyl)oxy)aniline (5b)**

The titled compound was obtained as brown crystals (0.68g, 97%); m.p. 60-62°C (as reported).

**4-((4-Chlorobenzyl)oxy)aniline (5c):**

The titled compound was obtained as brown crystals (0.60g, 84%); m.p. 108-109°C (as reported).

**4-((4-Bromobenzyl)oxy)aniline (5d)**:

The titled compound was obtained as brown crystals (0.71g, 85%); m.p. 110-112°C (as reported).

**4-((4-Methylbenzyl)oxy)aniline (5e)**:

The titled compound was obtained as brown crystals (0.41g, 64%); m.p. 105-106°C (as reported).

**4-((4-Triflouromethylbenzyl)oxy)aniline (5f)**:

The titled compound was obtained as brown crystals (0.52g, 65%); m.p. 108-110°C,( M.wt. = 267.25, C_14_H_12_F_3_NO); **^1^H NMR (400 MHz, DMSO-*d*_6_)** δ 7.43(d, *J* = 8.3 Hz, 2H, Ar-H), 7.28 (d, *J* = 8.3 Hz, 2H, Ar-H), 6.77 (d, *J* = 6.6 Hz, 2H, Ar–H), 6.62 (d, *𝐽* = 6.6 Hz, 2H, Ar–H), 5.22 (s, 2H, O-CH_2_). 4.62 (s, 2H, NH D_2_O exchangeable).

### 5.1.2.1.1. Supplementary figure 2:

**4-Nitro-1-(substituted-phenoxy)benzene (6a,b)**:[7]

**General Procedure:**

A mixture of 1-fluoro-4-nitrobenzene (0.4 g, 2.85 mmol), the respective substituted phenol (*viz*; 4-bromophenol, 4-chloro-3-fluoro phenol) (2.76 mmol) and anhydrous K_2_CO_3_ (1.9 g, 13.8 mmol) in dry DMF (10 mL) was heated at 50 ^o^C for 24 hours. After cooling, the reaction mixture was poured onto ice/water (100 mL). The precipitated solid was filtered, washed with water, and dried to afford the titled compounds **(6a,b**).

**4-Nitro-1-(4-bromophenoxy)benzene (6a)**:[8]

The titled compound was obtained as off-white powder (0.84 g, 100%); m.p. 96-98 ^o^C (as reported).[8]

**4-Nitro-1-(3-fluoro-4-chlorophenoxy)benzene (6b)**:

The titled compound was obtained as off-white powder (0.72 g, 94%); m.p.74-76 ^o^C (as reported).

**4-(Substituted-phenoxy)aniline (7a,b)**:

**General Procedure:**

A mixture of the relevant nitro derivative **(6a,b)** (1.5 mmol) and stannous chloride dihydrate (1.69 g, 7.5 mmol) in EtOAc (20 mL) was heated at reflux temperature (80 ^o^C) for 24 hours. After cooling, ice-cold water (100 mL) was added to the reaction mixture and then was separated, where the organic layer was washed multiple times with aqueous 10% NaOH (20 mL) then washed with brine (50 mL). The organic layer was separated, dried over anhydrous Na_2_SO_4_ and evaporated under vacuum to afford crystals of the titled compounds **(7a,b)**.

**4-(4-Bromophenoxy)aniline (7a):**

The titled compound was obtained as brown crystals (0.36 g, 92%); m.p. 76-78^o^C (as reported).

**4-(3-Fluoro-4-chlorophenoxy)aniline (7b):**

The titled compound was obtained as brown crystals (0.32 g, 90%); m.p. 71-72^o^C (as reported).

### 5.1.2.2. Supplementary figure 3:

**Ethyl 2-aminobenzo[*d*]thiazole-6-carboxylate (8)**: [9]

A stirred solution of ethyl 4-aminobenzoate (benzocaine) (1.73 g, 10.5 mmol) and potassium thiocyanate (3.06 g, 31.5 mmol) in glacial acetic acid (20 mL) was cooled to 0 ^o^C. To this solution bromine (99%) (0.54 mL, 10.5 mmol) was added dropwise at such a rate to keep the temperature below 10 °C throughout the addition. Stirring was continued for an additional 3 hours at room temperature and the separated salt was filtered, washed with acetic acid and dried, then the residue was dissolved in hot water and neutralized with aqueous ammonia solution (25%, 50 mL), the precipitated solid was filtered, washed with water and dried, recrystallized from ethanol/water to afford the titled compound **(8)** as white crystals (2.10 g, 90%); m.p. 241-242^o^C (as reported).[9]

***N*-(6-Nitrobenzo[*d*]thiazol-2-yl)naphthalene-2-sulfonamide (15) and *N*-(6-Aminobenzo[*d*]thiazol-2-yl) naphthalene-2-sulfonamide (16)**

Compounds 15 and 16 were synthesized according to the reported procedure [10] and all data were as reported.

***Ethyl 2-(naphthalene-2-sulfonamido)benzo[d]thiazole-6-carboxylate (5):***

To a solution of ethyl 2-aminobenzo[*d*]thiazole-6-carboxylate **(4)** (4.44g, 20 mmol) in dry pyridine (10 mL), 2-naphthylsulfonyl chloride (6.80 g, 30 mmol) was added portion-wise. The solution turned yellow upon addition. After 5 minutes of stirring at room temperature, the mixture was heated (70-80 °C) for 2 hours. The mixture was allowed to cool to room temperature then poured on a bed of ice, and the resulting solid was filtered, washed with water and dried to afford the titled compound **(5)** as orange solid with a yield (7.66 g, 93%); m.p. 294-296**°**C; (M.wt. = 412.48, C_20_H_16_N_2_O_4_S_2_); **^1^H NMR (400 MHz, DMSO-*d*_6_)** δ 13.40 ( s, 1H, NH sulfonamide D_2_O exchangeable), 8.54 (s, 1H, naphthyl H^1^), 8.18 (d, *J*= 7.5 Hz, 1H, naphthyl), 8.11 (d, *J*= 8.5 Hz, 1H, benzothiazole), 8.03 (d, *J*= 7.5 Hz, 1H, naphthyl), 7.96 (s, 1H, benzothiazole), 7.88 (d, *J*= 8.5 Hz, 1H, benzothiazole),7.73-7.61 (m, 2H, naphthyl), 7.24 (t, *J* = 7.0 Hz, 2H, naphthyl), 4.3 (q, *J*= 7.1 Hz, 2H, CH_3_-**CH_2_**-O-), 1.31 (t, *J* = 7.1 Hz, 3H, **CH_3_**-CH_2_-O-).

***2-(Naphthalene-2-sulfonamido)benzo[d]thiazole-6-carboxylic acid (6)****:*

Ethyl 2-(naphthalene-2-sulfonamido)benzo[*d*]thiazole-6-carboxylate **(5)** (2.84 g, 6.9 mmol) was added to a solution of LiOH.H_2_O (0.579 g, 13.8 mmo) in ethanol (50%, 70 mL).The mixture was heated under reflux for 2 hours. The resulting solution was allowed to cool to room temperature then added to 10% HCl/ice (100 mL) with continued stirring. The resulting solid was filtered, washed with water and allowed to dry to afford the titled compound **(6)** as buff solid with a yield (2.25 g ,85%); m.p. 208-210**°**C; (M.wt.= 384.43, C_18_H_12_N_2_O_4_S_2_); **^1^H NMR (400 MHz, DMSO-*d6*)** δ 13.40 ( s, 1H, NH sulfonamide D_2_O exchangeable), 12.20 (s, 1H, COOH, D_2_O exchangeable), 8.54 (s, 1H, naphthyl H^1^), 8.18 (d, J= 7.5 Hz, 1H, naphthyl), 8.11 (d, J= 8.5 Hz, 1H, benzothiazole), 8.03 (d, J= 7.5 Hz, 1H, naphthyl), 7.96 (s, 1H, benzothiazole), 7.88 (d, J= 8.5 Hz, 1H, benzothiazole), 7.73-7.68 (m, 2H, naphthyl), 7.24 (t, J = 7.0 Hz, 2H, naphthyl).

***N-(6-(4-Substituted-piperazine-1-carbonyl)benzo[d]thiazol-2-yl)naphthalene-2-sulfonamide (7a-i)****:*

**General Procedure:**

A mixture of 2-(Naphthalene-2-sulfonamido)benzo[*d*]thiazole-6-carboxylic acid **(6)** (0.19g , 0.5 mmol), the respective 1-substituted piperazine (*viz*;1-phenyl piperazine, 1-(2-fluorophenyl)piperazine, 1-(2-methoxyphenyl)piperazine, 1-(3,4 dichlorophenyl)piperazine , 1-(4-chlorophenyl)piperazine, 1-benzhydrylpiperazine, 1-(*trans*-cinnamyl)piperazine, 1-piperonylpiperazine and 1-(2-tetrahydrofuroyl)-piperazine) (0.5 mmol), EDC.HCl (0.19g, 1mmol) and DMAP (0.12g, 1mmol) was stirred in dry DMF (5 mL) under N_2_ atmosphere for 72 hours at room temperature. The mixture was added to ice/H_2_O (50 mL) with continued stirring. The resulting solid was filtered, dried and then purified by column chromatography (gradient elution starting from (DCM/MeOH 9.9:0.1) and increasing polarity to (DCM/MeOH 9.7:0.3)) to afford the titled compounds **(7a-i)**.

***N-(6-(4-Phenylpiperazine-1-carbonyl)benzo[d]thiazol-2-yl)naphthalene-2-sulfonamide (7a)****:*

The titled compound was obtained as white solid (0.16 g, 60 %); m.p 282-284 °C; **^1^H NMR (400 MHz, DMSO-*d*_6_)** δ 13.40 ( s, 1H, NH sulfonamide D_2_O exchangeable), 8.54 (s, 1H, naphthyl H^1^), 8.18 (d, *J* = 7.5 Hz, 1H, naphthyl), 8.11 (d, *J* = 8.5 Hz, 1H, benzothiazole), 8.03 (d, *J* = 7.5 Hz, 1H, naphthyl), 7.97 (s, 1H, benzothiazole), 7.89 (d, *J* = 8.5 Hz, 1H, benzothiazole), 7.71- 7.64 (m, 2H, naphthyl), 7.47 (d, *J* = 8.1 Hz, 1H, naphthyl), 7.35 (d, J= 8.2 Hz, 1H, naphthyl), 7.23 (t, *J* = 7.6 Hz, 2H, Ar-H), 6.95 (d, *J* = 8.0 Hz, 2H, Ar-H), 6.81 (t, *J* = 7.1 Hz, 1H, Ar-H), 3.35 (s, 4H, piperazine), 3.16 (s, 4H, piperazine); **MS:** (M.wt.: 528.65) : *m/z* 531.13 [M+2, (2.9%)], 529.13 [M+1, (33.5%)], 528.13(100%); **Anal.** Calcd for C_28_H_24_N_4_O_3_S_2_: C, 63.62; H, 4.58; N, 10.60; Found: C, 63.42; H, 4.60; N, 10.55.

***N*-(6-(4-(2-Fluorophenyl)piperazine-1-carbonyl)benzo[*d*]thiazol-2-yl)naphthalene-2-sulfonamide (7b):**

The titled compound was obtained as white solid (0.16 g, 60 %); m.p 290-292 °C; **^1^H NMR (400 MHz, DMSO-*d*_6_)** δ 13.41 ( s, 1H, NH sulfonamide D_2_O exchangeable), 8.54 (s, 1H, naphthyl H^1^), 8.19 (d, *J* = 7.5 Hz, 1H, naphthyl), 8.11 (d, *J* = 8.5 Hz, 1H, benzothiazole), 8.04 (d, *J* = 7.5 Hz, 1H, naphthyl), 7.97 (s, 1H, benzothiazole), 7.87 (d, *J* = 8.5 Hz, 1H, benzothiazole), 7.72-7.65 (m, 2H, naphthyl), 7.48 (d, *J* = 8.1 Hz, 1H, naphthyl), 7.35 (d, J= 8.2 Hz, 1H, naphthyl), 7.18-7.11 (m, 2H, Ar-H), 7.07-7.03 (m, 2H, Ar-H), 3.35 (s, 4H, piperazine), 3.16 (s, 4H, piperazine); MS: (M.wt.: 546.64) : *m/z* 548.18 [M+2, (15%)], 547.18 [M+1, (33.5%)], 546.18(100%); Anal. Calcd for C_28_H_23_FN_4_O_3_S_2_: C, 61.52; H, 4.24; F, 3.48; N, 10.25; Found: C, 61.42; H, 4.30; N, 10.25.

***N*-(6-(4-(2-Methoxyphenyl)piperazine-1-carbonyl)benzo[*d*]thiazol-2-yl)naphthalene-2-sulfonamide (7c):**

The titled compound was obtained as off-white solid (0.14 g, 50 %); m.p 258-260 °C; **^1^H NMR (400 MHz, DMSO-*d*_6_)**  δ 13.40 (s, 1H, NH sulfonamide D_2_O exchangeable), 8.55 (s, 1H, naphthyl H^1^), 8.19 (d, *J* = 7.5 Hz, 1H, naphthyl), 8.11 (d, *J* = 8.5 Hz, 1H, benzothiazole), 8.03 (d, *J* = 7.5 Hz, 1H, naphthyl), 7.96 (s, 1H, benzothiazole), 7.89 (d, *J* = 8.5 Hz, 1H, benzothiazole), 7.71-7.64 (m, 2H, naphthyl), 7.48 (d, *J* = 8.1 Hz, 1H, naphthyl), 7.35 (d, J= 8.2 Hz, 1H, naphthyl), 6.96 ( s, 2H, Ar-H), 6.89 (s, 2H, Ar-H), 3.78 (s, 3H, O-CH_3_), 3.36 (s, 4H, piperazine), 2.97 (s, 4 H, piperazine); MS: (M.wt.: 558.67) : *m/z* 560.14 [M+2, (11%)], 559.14 [M+1, (34.6%)], 558.14(100%); Anal. Calcd for C_29_H_26_N_4_O_4_S_2_: C, 62.35; H, 4.69; N, 10.03; Found: C, 62.40; H, 4.60; N, 10.05.

***N*-(6-(4-(4-Chlorophenyl)piperazine-1-carbonyl)benzo[*d*]thiazol-2-yl)naphthalene-2-sulfonamide (7d):**

The titled compound was obtained as off-white solid (0.14 g, 50 %); m.p 260-262°C; **^1^H NMR (400 MHz, DMSO-*d*_6_)**  δ 13.42 (s, 1H, NH sulfonamide D_2_O exchangeable), 8.54 (s, 1H, naphthyl H^1^), 8.18 (d, *J* = 7.6 Hz, 1H, naphthyl), 8.11 (d, *J* = 8.7 Hz, 1H, benzothiazole), 8.03 (d, *J* = 7.7 Hz, 1H, naphthyl), 7.96 (s, 1H, benzothiazole), 7.89 (d, *J* = 8.6 Hz, 1H, benzothiazole), 7.71-7.64 (m, 2H, naphthyl), 7.47 (d, *J* = 8.2 Hz, 1H, naphthyl H^6^), 7.35 (d, *J* = 8.2 Hz, 1H, naphthyl), 7.25 (d, *J* = 8.8 Hz, 2H, Ar-H), 6.96 (d, *J* = 8.8 Hz, 2H, Ar-H), 3.33 (s, 4H, piperazine), 3.17 (s, 4H, piperazine); MS: (M.wt.: 563.09) : *m/z* 565.09 [M+2, (13.3%)], 564.09 [M+1, (46.6%)], 562.09(100%); Anal. Calcd for C_28_H_23_ClN_4_O_3_S_2_: C, 59.72; H, 4.12; Cl, 6.30; N, 9.95; Found: C, 59.68; H, 4.20; N, 10.10.

***N*-(6-(4-(3,4-Dichlorophenyl)piperazine-1-carbonyl)benzo[*d*]thiazol-2-yl)naphthalene-2-sulfonamide (7e)**:

The titled compound was obtained as white solid (0.15 g, 50 %); m.p 272-274°C; **^1^H NMR (400 MHz, DMSO-*d*_6_)** δ 13.38 (s, 1H, NH sulfonamide D_2_O exchangeable), 8.54 (s, 1H, naphthyl H^1^), 8.18 (d, *J* = 7.8 Hz, 1H, naphthyl), 8.11 (d, *J* = 8.7 Hz, 1H, benzothiazole), 8.03 (d, *J* = 7.8 Hz, 1H, naphthyl), 7.96 (s, 1H, benzothiazole), 7.87 (d, *J* = 8.6 Hz, 1H, benzothiazole),7.71-7.65 (m, 2H, naphthyl), 7.48 (d, *J* = 8.3 Hz, 1H, naphthyl), 7.42 (d, *J* = 9.0 Hz, 1H, Ar-H), 7.35 (d, *J* = 8.3 Hz, 1H, naphthyl), 7.15 (dd, *J* =9.0, 2.6Hz, 1H, Ar-H), 6.95 (dd, *J* = 9.0, 2.6 Hz, 1H, Ar-H), 3.35 (s, 4H, piperazine), 3.24 (s, 4H, piperazine); **MS:** (M.wt.: 597.54): *m/z* 599.07 [M+2, (24.5%)], 598.07 [M+1, (73.9%)], 596.05 (100%); **Anal.** Calcd for C_28_H_22_Cl_2_N_4_O_3_S_2_: C, 56.28; H, 3.71; Cl, 11.87; N, 9.38; Found: C, 56.38; H, 3.52; N, 9.40.

***N*-(6-(4-Benzhydrylpiperazine-1-carbonyl)benzo[*d*]thiazol-2-yl)naphthalene-2-sulfonamide (7f)**:

The titled compound was obtained as white solid (0.12 g, 40 %); m.p 200-202°C; **^1^H NMR (400 MHz, DMSO-*d*_6_)** δ 13.35 (s, 1H, NH sulfonamide D_2_O exchangeable), 8.51 (s, 1H, naphthyl H^1^), 8.17 (d, *J* = 7.7 Hz, 1H, naphthyl), 8.09 (d, *J* = 8.7 Hz, 1H, benzothiazole), 8.02 (d, *J* = 7.8 Hz, 1H, naphthyl), 7.96 (s, 1H, benzothiazole), 7.85 (dd, *J* = 8.7, 1.6 Hz, 1H, benzothiazole), 7.70-7.63 (m, 2H, naphthyl),7.44- 7.37 (m, 5H, benzhydryl), 7.32- 7.28 (t, *J* = 7.2 Hz, 5H, benzhydryl), 7.19 (t, *J* = 7.2 Hz, 2H, naphthyl), 4.35 (s, 1H, CH benzhydryl), 3.54 (s, 4H, piperazine), 2.33 (s, 4H, piperazine); **MS:** (M.wt.: 618.77) : *m/z* 620.17 [M+2, (17.9%)], 619.18 [M+1, (40%)], 618 (100%); **Anal.** Calcd for C_35_H_30_N_4_O_3_S_2_: C, 67.94; H, 4.89; N, 9.05; Found: C, 67.78; H, 4.80; N, 9.00.

**(*E*)-*N*-(6-(4-Cinnamylpiperazine-1-carbonyl)benzo[*d*]thiazol-2-yl)naphthalene-2-sulfonamide (7g):**

The titled compound was obtained as white solid (0.10 g, 40 %); m.p 240-242°C; **^1^H NMR (400 MHz, DMSO-*d*_6_)** δ 13.35 (s, 1H, NH sulfonamide D_2_O exchangeable), 8.50 (s, 1H, naphthyl H^1^), 8.15 (d, *J* = 7.6 Hz, 1H, naphthyl), 8.06 (d, *J* = 8.7 Hz, 1H, benzothiazole), 8.00 (d, *J* = 7.8 Hz, 1H, naphthyl), 7.87 (s, 1H, benzothiazole), 7.85 (dd, *J* = 8.7, 1.6 Hz, 1H, benzothiazole), 7.68 -7.62 (m, 2H, naphthyl), 7.45 (d, *J* = 7.5 Hz, 1H, naphthyl), 7.40 – 7.29 (m, 5H, Ar-H), 7.26 (t, *J* = 7.1 Hz, 1H, naphthyl), 6.61 (d, *J* = 15.4 Hz, 1H, Ar-**CH**=CH-CH_2_-), 6.30 (dt, *J* = 15.4, 6.6 Hz, 1H, Ar-CH=**CH**-CH_2_-), 3.53 (s, 4H, piperazine), 3.32 (d, *J* = 6.4 Hz, 2H,- **CH_2_**-N), 2.62 (s, 4H, piperazine); **MS:** (M.wt.: 568.71) : *m/z* 571.15 [M+2, (3.3%)], 570.15 [M+1, (16.3%)], 569.15[M^+^,35.8%], 568.15 (100%); **Anal.** Calcd for C_31_H_28_N_4_O_3_S_2_: C, 65.47; H, 4.96; N, 9.85; Found: C, 65.48; H, 4.89; N, 9.73.

***N*-(6-(4-(Benzo[*d*]dioxol-5-ylmethyl)piperazine-1-carbonyl)benzo[*d*]thiazol-2-yl)naphthalene-2-sulfonamide (7h)**:

The titled compound was obtained as white solid (0.12 g, 40 %); m.p. 178-180°C; **^1^H NMR (400 MHz, DMSO-*d*_6_)** δ 13.35 (s, 1H, NH sulfonamide D_2_O exchangeable), 8.50 (s, 1H, naphthyl H^1^), 8.17 (d, *J* = 7.9 Hz, 1H, naphthyl), 8.09 (d, *J* = 8.7 Hz, 1H, benzothiazole), 8.02 (d, *J* = 7.8 Hz, 1H, naphthyl), 7.87 (d, *J* = 9.5 Hz, 2H, benzothiazole), 7.69-6.62 (m, 2H, naphthyl), 7.38 (d, *J* = 8.3 Hz, 1H, naphthyl), 7.32 (d, *J* = 8.3 Hz, 1H, naphthyl), 6.92 – 6.86 (m, 2H, Ar-H), 6.79 (d, *J* = 7.9 Hz, 1H, Ar-H), 6.02 (s, 2H, CH_2_- dioxolane), 3.66 (s, 2H,-**CH_2_**-piprazine), 3.35 (s, 4H, piperazine), 2.47 (s, 4H, piperazine); **MS:** (M.wt.: 586.68) : *m/z* 589.13 [M+2, (4.4%)], 588.14 [M+1, (16.4%)], 586.13 (100%); **Anal.** Calcd for C_30_H_26_N_4_O_5_S_2_: C, 61.42; H, 4.47; N, 9.55; Found: C, 61.50; H, 4.49; N 9.25.

***N*-(6-(4-(Tetrahydrofuran-2-carbonyl)piperazine-1-carbonyl)benzo[*d*]thiazol-2-yl)naphthalene-2-sulfonamide (7i):**

The titled compound was obtained as buff solid (0.08 g, 30 %); m.p. 230-232°C; **^1^H NMR (400 MHz, DMSO-*d*_6_)** δ 13.41 (s, 1H, NH sulfonamide D_2_O exchangeable), 8.54 (s, 1H, naphthyl H^1^), 8.17 (d, *J* = 7.6 Hz, 1H, naphthyl), 8.10 (d, *J* = 8.7 Hz, 1H, benzothiazole), 8.02 (d, *J* = 7.8 Hz, 1H, naphthyl), 7.93 (s, 1H, benzothiazole), 7.88 (d, *J* = 8.6 Hz, 1H, benzothiazole), 7.68- 7.52 (m, 2H, naphthyl), 7.48 (d, *J* = 8.3 Hz, 1H, naphthyl), 7.35 (d, *J* = 8.3 Hz, 1H, naphthyl), 4.66 (s, 1H, furoyl H^2^), 3.76 ( t, J=14.36 ,2H, furoyl H^5^) , 3.53 (s, 4H, piperazine), 2.47 (s, 4H, piperazine), 2.09-2.04 (m, 4H, furoyl); **MS:** (M.wt.: 550.65) : *m/z* 552.13 [M+2, (15.3%)], 551.13 [M+1, (30.3%)], 550.13 (100%); **Anal.** Calcd for C_27_H_26_N_4_O_5_S_2_: C, 58.89; H, 4.76; N, 10.17; Found: C, 58.68; H, 4.79; N10.25.

***N*-(4-Substituted-benzyl)oxy)phenyl)-2-(naphthalene-2-sulfonamido)benzo[*d*]thiazole-6-carboxamide (8a-f):**

**General Procedure:**

A mixture of 2-(naphthalene-2-sulfonamido)benzo[*d*]thiazole-6-carboxylic acid **(6)** (0.19 g, 0.5 mmol), the respective amino compounds **(Ia-f)** (0.5 mmol), EDC.HCl (0.19g, 1mmol) and DMAP (0.12g, 1mmol) was stirred in dry DMF (5 mL) under N_2_ atmosphere for 72 hours at room temperature. The mixture was added to ice/H_2_O (50 mL) with continued stirring. The resulting solid was filtered, dried and then purified by column chromatography (gradient elution starting from (DCM/MeOH 9.9:0.1) and increasing polarity to (DCM/ MeOH 9.7:0.3)) to afford the titled compounds **(8a-f)**.

***N*-(4-((3-Fluorobenzyl)oxy)phenyl)-2-(naphthalene-2-sulfonamido)-benzo[*d*]thiazole-6-carboxamide (8a):**

The titled compound was obtained as off-white solid (0.19 g, 65 %); m.p. 146-148°C; **^1^H NMR (400 MHz, DMSO-*d*_6_)** δ 9.95 (s,1H, NH amide D_2_O exchangeable), 8.32 (s, 1H, naphthyl), 8.13 (s, 1H, benzothiazole), 8.11 (d, J = 7.7 Hz, 1H, naphthyl), 8.02 (d, *J* = 7.7 Hz, 1H, naphthyl), 7.89 (d, *J* = 8.3 Hz, 1H, naphthyl), 7.80 (d, *J* = 8.5 Hz, 1H, benzothiazole), 7.74 (s, 1H, Ar-H), 7.71 -7.66 (m, 2H, naphthyl), 7.57 (d, J = 8.6 Hz, 1H, benzothiazole), 7.42(d, *J* = 8.6 Hz, 2H, Ar-H), 7.34 (d, *J* = 8.3 Hz, 1H, naphthyl), 7.19 (s, 1 H, Ar-H), 7.02 (d, *J* = 8.6 Hz, 2H, Ar-H), 6.96 (d, *J* = 8.7 Hz, 2H, Ar-H), 5.12 (s, 2H, O-CH_2_); **^13^C NMR (101 MHz, DMSO)** δ 165.36, 163.86, 161.43, 156.67, 155.88, 149.78, 145.56, 140.16, 137.89, 134.12, 134.02, 132.05, 130.96, 129.36, 128.06, 127.43, 126.02, 125.64, 125.07, 122.98, 122.25, 121.63, 121.04, 120.38, 116.43, 115.23, 114.99, 114.59, 114.37, 107.28, 69.86**; MS:** (M.wt.: 583) : *m/z* 585.10 [M+2, (16.3%)], 584.11 [M+1, (40%)], 583.10 (100%); **Anal.** Calcd for C_31_H_22_FN_3_O_4_S_2_: C, 63.79; H, 3.80; F, 3.26; N, 7.20; Found: C, 63.57; H, 3.69; N 7.25.

***N*-(4-((3-Chlorobenzyl)oxy)phenyl)-2-(naphthalene-2-sulfonamido)benzo[d]thiazole-6-carboxamide (8b):**

The titled compound was obtained as off-white solid (0.18 g, 60 %); m.p. 198-200°C; **^1^H NMR (400 MHz, DMSO-*d*_6_)** δ 9.94 (s,1H, NH amide D_2_O exchangeable), 8.32 (s, 1H, naphthyl), 8.12 (s, 1H, benzothiazole), 8.07 (d, J = 7.7 Hz, 1H, naphthyl), 8.01 (d, *J* = 7.7 Hz, 1H, naphthyl), 7.89 (d, *J* = 8.3 Hz, 1H, naphthyl), 7.80 (d, *J* = 8.5 Hz, 1H, benzothiazole), 7.74 (s, 1H, Ar-H), 7.72 -7.66 (m, 2H, naphthyl), 7.57 (d, J = 8.6 Hz, 1H, benzothiazole), 7.42(d, *J* = 8.6 Hz, 2H, Ar-H), 7.34 (d, *J* = 8.3 Hz, 1H, naphthyl), 7.19 (s, 1 H, Ar-H), 6.99 (d, *J* = 8.6 Hz, 2H, Ar-H), 6.96 (d, *J* = 8.7 Hz, 2H, Ar-H), 5.12 (s, 2H, O-CH_2_); **MS:** (M.wt.: 600.11) : *m/z* 602.08 [M+2, (11 %)], 601.07 [M+1, (48.3 %)], 600.07 [M^+^, (33 %)],599.10 (100%); **Anal.** Calcd for C_31_H_22_ClN_3_O_4_S_2_: C, 62.04; H, 3.70; Cl, 5.91; N, 7.00; Found: C, 62.00; H, 3.69; N 7.15.

***N*-(4-((4-Chlorobenzyl)oxy)phenyl)-2-(naphthalene-2-sulfonamido)benzo[*d*]thiazole-6-carboxamide (8c)**:

The titled compound was obtained as white solid (0.16 g, 55 %); m.p. 296-298°C; **^1^H NMR (400 MHz, DMSO-*d*_6_)** δ 10.22 (s, 1H, NH amide D_2_O exchangeable), 8.51 (s, 1H, naphthyl H1), 8.31 (s, 1H, benzothiazole), 8.15 (d, *J* = 7.7 Hz, 1H, naphthyl), 8.06 (d, *J* = 8.6 Hz, 1H, benzothiazole), 8.01 (d, *J* = 7.5 Hz, 1H, naphthyl), 7.94 (d, *J* = 8.3 Hz, 1H, naphthyl), 7.86 (d, *J* = 8.5 Hz, 1H, benzothiazole), 7.80 (d, *J* = 8.8 Hz, 2H, Ar-H), 7.70-7.65 (m, 2H, naphthyl), 7.49 (d, *J* = 8.6 Hz, 2H, Ar-H), 7.38 (d, *J* = 8.3 Hz, 1H, naphthyl), 7.24 (d, *J* = 8.7 Hz, 2H, Ar-H), 6.97 (d, *J* = 8.7 Hz, 2H, Ar-H), 5.19 (s, 2H, O-CH_2_-); **MS**: (M.wt.: 600.11) : m/z 602.08 [M+2, (11 %)], 601.07 [M+1, (47.5 %)], 600.07 [M+, (33.9%)], 599.10 (100%); **Anal.** Calcd for C_31_H_22_ClN_3_O_4_S_2_: C, 62.04; H, 3.70; Cl, 5.91; N, 7.00; Found: C, 62.00; H, 3.65; N 7.05.

***N*-(4-((4-Bromobenzyl)oxy)phenyl)-2-(naphthalene-2-sulfonamido)benzo[*d*]thiazole-6-carboxamide (8d)**:

The titled compound was obtained as white solid (0.14 g, 45 %); m.p. ≥ 300°C; ^1^H NMR (400 MHz, DMSO-*d*_6_) δ 10.13 (s, 1H, NH amide D_2_O exchangeable), 8.52 (s, 1H, naphthyl H^1^), 8.33 (s, 1H, benzothiazole), 8.16 (d, *J* = 7.7 Hz, 1H, naphthyl), 8.07 (d, *J* = 8.6 Hz, 1H, benzothiazole), 8.01 (d, *J* = 7.5 Hz, 1H, naphthyl), 7.90 (d, *J* = 8.3 Hz, 1H, naphthyl), 7.86 (d, *J* = 8.5 Hz, 1H, benzothiazole), 7.80 (d, *J* = 8.8 Hz, 2H, Ar-H), 7.71-7.66 (m, 2H, naphthyl), 7.55 (d, *J* = 8.6 Hz, 2H, Ar-H), 7.43 (d, *J* = 8.3 Hz, 1H, naphthyl), 7.37 (d, *J* = 8.7 Hz, 2H, Ar-H), 7.00 (d, *J* = 8.7 Hz, 2H, Ar-H ), 5.08 (s, 2H, O-CH_2_-); MS: (M.wt.: 644.56 ) : *m/z* 647.02 [M+2, (15.9%)], 646.02 [M+1, (37.4 %)], 644.02 [M^+^, (31.4%)], 645.02 (100%); Anal. Calcd for C_31_H_22_BrN_3_O_4_S_2_: C, 57.77; H, 3.44; Br, 12.40; N, 6.52; Found: C, 57.79; H, 3.60; N 6.35.

***N*-(4-((4-Methylbenzyl)oxy)phenyl)-2-(naphthalene-2-sulfonamido)benzo[*d*]thiazole-6-carboxamide (8e):**

The titled compound was obtained as faint purple solid (0.1 g, 35 %); m.p ≥ 300°C; **^1^H NMR (400 MHz, DMSO-*d*_6_)** δ 10.14 (s,1H, NH amide D_2_O exchangeable), 8.54 (s, 1H, naphthyl H^1^), 8.35 (s, 1H, benzothiazole), 8.19 (d, *J* = 6.7 Hz, 1H, naphthyl), 8.12 (d, *J* = 9.0 Hz, 1H, benzothiazole), 8.03 (d, *J* = 6.6 Hz, 1H, naphthyl), 7.94 (d, *J* = 9.2 Hz, 1H, benzothiazole), 7.89 (d, *J* = 7.1 Hz, 2H, Ar-H), 7.70 -7.68 (m, *J* = 6.7 Hz, 2H, naphthyl), 7.39 (d, *J* = 8.4 Hz, 2H, Ar-H), 7.33 (d, *J* = 6.6 Hz, 2H, naphthyl), 7.20 (d, *J* = 7.1 Hz, 2H, Ar-H), 6.99 (d, *J* = 8.5 Hz, 2H, Ar-H), 5.07 (s, 2H, O-CH_2_-), 2.32(s, 3H, CH_3_); **MS:** (M.wt.: 579.69 ): *m/z* 582 [M+2, (3.1%)], 581 [M+1, (16.8 %)], 580 [M^+^, (37.8%)], 579 (100%); **Anal.** Calcd for C_32_H_25_N_3_O_4_S_2_: C, 66.30; H, 4.35; N, 7.25; Found: C, 66.31; H, 4.50; N 7.35.

***N*-(4-((4-Trifluormethylbenzyl)oxy)phenyl)-2-(naphthalene-2-sulfonamido)benzo-[*d*]thiazole-6-carboxamide *)*8f)**:

The titled compound was obtained as faint buff solid (0.1 g, 30 %); m.p ≥ 300°C; **^1^H NMR (400 MHz, DMSO-*d*_6_)** δ 10.15 (s,1H, NH amide D_2_O exchangeable), 8.54 (s, 1H, naphthyl H^1^), 8.35 (s, 1H, benzothiazole), 8.19 (d, *J* = 6.7 Hz, 1H, naphthyl), 8.11 (d, *J* = 9.0 Hz, 1H, benzothiazole), 8.03 (d, *J* = 6.6 Hz, 1H, naphthyl), 7.95 (d, *J* = 9.2 Hz, 1H, benzothiazole), 7.89 (d, *J* = 7.1 Hz, 2H, Ar-H), 7.69-7.63 (m, 2H, naphthyl), 7.39 (d, *J* = 8.4 Hz, 2H, Ar-H), 7.34 (d, *J* = 6.6 Hz, 2H, naphthyl), 7.21 (d, *J* = 7.1 Hz, 2H, Ar-H), 6.99 (d, *J* = 8.5 Hz, 2H, Ar-H), 5.04 (s, 2H, O-CH_2_-); **MS:** (Mwt.: 633.66 ) : *m/z* 635.10 [M+2, (15.7%)], 634.10 [M+1, (37.5 %)], 633.1 (100%); **Anal.** Calcd for C_32_H_22_F_3_N_3_O_4_S_2_: C, 60.65; H, 3.50; F, 8.99; N, 6.63; Found: C, 60.35; H, 3.50; N 6.55.

***N*-(4-(Substituted-phenoxy)phenyl)-2-(naphthalene-2-sulfonamido)benzo[*d*]thiazole-6-carboxamide (9a,b)**:

**General Procedure:**

A mixture of 2-(naphthalene-2-sulfonamido)benzo[*d*]thiazole-6-carboxylic acid **(6)** (0.19 g, 0.5 mmol), the respective amino compounds **(IIa,b)** (0.5 mmol), EDC.HCl (0.19g, 1mmol) and DMAP (0.12g, 1mmol) was stirred in dry DMF (5 mL) under N_2_ atmosphere for 72 hours at room temperature. The mixture was added to ice/H_2_O (50 mL) with continued stirring. The resulting solid was filtered, dried and then purified by column chromatography (gradient elution starting from (DCM/MeOH 9.9:0.1) and increasing polarity to (DCM/ MeOH 9.7:0.3)) to afford the titled compounds **(9a,b)**.

***N*-(4-(4-Chloro-3-fluorophenoxy)phenyl)-2-(naphthalene-2-sulfonamido)benzo[*d*]thiazole-6-carboxamide (9a):**

The titled compound was obtained as off-white solid (0.1 g, 35 %); m.p 294 -296 °C; **^1^H NMR (400 MHz, DMSO-*d*_6_)** δ 10.37 (s, 1H, NH amide D_2_O exchangeable), 8.56 (s, 1H, naphthyl H^1^), 8.40 (s, 1H, benzothiazole), 8.19 (d, *J* = 7.8 Hz, 1H, naphthyl), 8.11 (d, *J* = 8.4 Hz, 1H, benzothiazole), 8.04 (d, *J* = 7.8 Hz, 1H, naphthyl), 7.98 (d, *J* = 8.1 Hz, 1H, benzothiazole), 7.88 (d, *J* = 7.8 Hz, 1H, Ar-H), 7.83 (d, *J* = 7.2 Hz, 2H, Ar-H), 7.75 – 7.62 (m, 2H, naphthyl), 7.57 (t, J= 8.8 Hz, 1H, Ar-H), 7.40 (d, *J* = 7.8 Hz, 1H, naphthyl), 7.11 (t, *J* = 9.0 Hz, 3H, Ar-H & naphthyl H^7^), 6.84 (d, *J* = 7.8 Hz, 1H, Ar-H); **MS**: (M.wt.: 604.07 ): *m/z* 606.05 [M+2, (14.1%)], 605.05 [M+1, (47.9 %)], 603.05 (100%); **Anal.** Calcd for C_30_H_19_ClFN_3_O_4_S_2_ C, 59.65; H, 3.17; Cl, 5.87; F, 3.15; N, 6.96; Found: C, 60.05; H, 3.10; N 6.95.

***N*-(4-(4-Bromophenoxy)phenyl)-2-(naphthalene-2-sulfonamido)benzo[*d*]thiazole-6-carboxamide (9b):**

The titled compound was obtained as white solid (0.1 g, 30 %); m.p. 280-282°C; **^1^H NMR (400 MHz, DMSO-*d*_6_)** δ 10.33 (s, 1H, NH amide D_2_O exchangeable), 8.55 (s, 1H, naphthyl H^1^), 8.39 (s, 1H, benzothiazole H^7^), 8.19 (d, *J* = 7.7 Hz, 1H, naphthyl H^4^), 8.11 (d, *J* = 8.6 Hz, 1H, benzothiazole H^4^), 8.03 (d, *J* = 7.5 Hz, 1H, naphthyl H^3^), 7.97 (d, *J* = 8.3 Hz, 1H, naphthyl H^6^), 7.88 (d, *J* = 8.5 Hz, 1H, benzothiazole H^5^), 7.80 (d, *J* = 8.8 Hz, 2H, Ar-H), 7.68 (p, *J* = 6.7 Hz, 2H, naphthyl H^5,8^), 7.55 (d, *J* = 8.6 Hz, 2H, Ar-H), 7.40 (d, *J* = 8.3 Hz, 1H, naphthyl H^7^), 7.07 (d, *J* = 8.7 Hz, 2H, Ar-H), 6.96 (d, *J* = 8.7 Hz, 2H); **MS:** (M.wt.: 630.53 ): *m/z* 633.01 [M+2, (15.7%)], 632.10 [M+1, (35.2%)], 631.1 (100%); **Anal.** Calcd for C_30_H_20_BrN_3_O_4_S_2_: C, 57.15; H, 3.20; Br, 12.67; N, 6.66; Found: C, 57.35; H, 3.50; N 6.55.

**Ethyl 2-(3-nitro-4-(phenethylamino)benzamido)benzo[*d*]thiazole-6-carboxylate (10)**:

A mixture of 3-nitro-4-(phenethylamino)benzoic acid **(3)** (2.86g, 10 mmol), ethyl 2-aminobenzo[*d*]thiazole-6-carboxylate **(4)** (2.22g, 10 mmol), EDC.HCl (4.1g, 20 mmol) and DMAP (2.59g, 20 mmol) was stirred in dry DMF (10 mL) under N_2_ atmosphere for 72 hours at room temperature. The mixture was added to ice/H_2_O (100 mL) with continued stirring. The resulting solid was filtered, washed with diethyl ether, dried and then purified by column chromatography (gradient elution starting from (DCM/MeOH 9.9:0.1) and increasing polarity to (DCM/MeOH 9.7:0.3)) to afford the titled compound **(10)** as orange solid (2.33 g, 95%)**;** m.p. 168 -170 °C; (M.wt.= 490.53, C_25_H_22_N_4_O_5_S); **^1^H NMR (400 MHz, DMSO-*d*_6_)** δ 10.30 (s, 1H, NH amide D_2_O exchangeable), 9.03 (s, 1H, NH D_2_O exchangeable), 8.79 (s,1H, benzothiazole), 8.44 (s, 1H, Ar-H), 8.14 (d, *J* = 7.8, 1H, Ar-H), 7.99 (d, *J* = 8.5 Hz, 1H, benzothiazole), 7.89 (d *, J* = 8.5, 1H, benzothiazole), 7.38 -7.30 (m, 5H, phenyl), 6.9 (d, *J* = 7.8, 1H, Ar-H), 4.24 (q, *J* = 7.1 Hz, 2H, CH_3-_**CH_2_**O-), 3.68 (t, *J* = 6.8 Hz, 2H, -N-**CH_2_**-CH_2_-), 2.98 (t, *J* = 6.8 Hz, 2H, -**CH_2_**-Ar), 1.31 (t, *J* = 7.1 Hz, 3H, **CH_3_**-CH_2_-).

**2-(3-Nitro-4-(phenethylamino)benzamido)benzo[*d*]thiazole-6-carboxylic acid (11)**:

The ester derivative **(10)** (3.38 g, 6.9 mmol) was added to a solution of LiOH.H_2_O (0.579 g, 13.8 mmol) in ethanol (50%, 70 mL). The mixture was heated under reflux for 2 hours. The resulting solution was allowed to cool to room temperature then added to 10% HCl/ice (100 mL) with continued stirring. The resulting solid was filtered, washed with water (100 mL) and allowed to dry to afford the titled compound **(11)** as yellow solid with a yield (2.71 g ,85%); m.p. 208-210**°**C; (M.wt.= 462.48, C_23_H_18_N_4_O_5_S); **^1^H NMR (400 MHz, DMSO-*d*_6_)** δ 12.20 (s, 1H, COOH, D_2_O exchangeable), 10.30 (s, 1H, NH amide D_2_O exchangeable), 9.03 (s, 1H, NH D_2_O exchangeable), 8.79 (s,1H, benzothiazole), 8.44 (s, 1H, Ar-H), 8.14 (d, J = 7.8, 1H, Ar-H), 7.99 (d, *J* = 8.5 Hz, 1H, benzothiazole), 7.89 (d *, J* = 8.5, 1H, benzothiazole), 7.38 -7.30 (m, 5H, phenyl), 7.04 (d, J = 7.8, 1H, Ar-H), 3.68 (t, J = 6.8 Hz, 2H, -N**-CH_2_**-CH_2_-), 2.98 (t, J = 6.8 Hz, 2H, -**CH_2_**-Ar).

**3-Nitro-4-(phenethylamino)-*N*-(6-(4-substituted-piperazine-1-carbonyl)benzo[*d*]thiazol-2-yl)benzamide (12a-e):**

**General Procedure:**

A mixture of 2-(3-nitro-4-(phenethylamino)benzamido)benzo[*d*]thiazole-6-carboxylic acid **(6)** (0.23g , 0.5 mmol), the respective 1-substituted piperazine (*viz*;1-(2-methoxyphenyl)piperazine, 1-(3,4dichlorophenyl)piperazine, 1-(benzhydryl) piperazine, 1-(*trans*-cinnamyl)piperazine and 1-(2-tetrahydrofuroyl) piperazine) (0.5 mmol), EDC.HCl (0.19g, 1mmol) and DMAP (0.12g, 1mmol) was stirred in dry DMF (5 mL) under N_2_ atmosphere for 72 hours at room temperature. The mixture was added to ice/H_2_O (50 mL) with continued stirring. The resulting solid was filtered, dried and then purified by column chromatography (gradient elution starting from (DCM/MeOH 9.9:0.1) and increasing polarity to (DCM/MeOH 9.7:0.3)) to afford the titled compounds **(12a-e)**.

***N*-(6-(4-(2-Methoxyphenyl)piperazine-1-carbonyl)benzo[*d*]thiazol-2-yl)-3-nitro-4-(phenethylamino)benzamide (12a):**

The titled compound was obtained as yellow solid (0.15g, 50%); m.p: 184-186°C; **^1^H NMR (400 MHz, DMSO-*d*_6_)** δ 10.30 (s, 1H, NH amide D_2_O exchangeable), 9.03 (s, 1H, NH D_2_O exchangeable), 8.79 (s,1H, benzothiazole), 8.44 (s, 1H, Ar-H), 8.14 (d, J = 7.8, 1H, Ar-H), 7.99 (d, *J* = 8.5 Hz, 1H, benzothiazole), 7.89 (d *, J* = 8.5, 1H, benzothiazole), 7.38 -7.30 (m, 5H, phenyl), 7.04 (d, J = 7.8, 1H, Ar-H), 6.95 ( d, J= 8.06, 2H, Ar-H), 6.88 (d, J= 8.11, 2H), 3.77 (s, 3H, O-CH_3_), 3.64 (t, J = 6.8 Hz, -2H, N**-CH_2_**-CH_2_-), 3.53 (s, 4H, piperazine), 2.93 (t, J = 6.8 Hz, 2H, -**CH_2_**-Ar), 2.32 (s, 4H, piperazine); **MS**: (M.wt.: 636.22): *m/z*, 638.22 [M+2, (8.8%)], 637.22 [M+1, (37%)], 636.22[M^+^,(100%)]; **Anal.** Calcd for C_34_H_32_N_6_O_5_S: C, 64.14; H, 5.07; N, 13.20; Found: C, 64.17; H, 5.09; N, 13.23.

***N*-(6-(4-(3,4-Dichlorophenyl)piperazine-1-carbonyl)benzo[*d*]thiazol-2-yl)-3-nitro-4-(phenethylamino)benzamide (12b):**

The titled compound was obtained as yellow solid (0.15g, 45%); m.p: 176-178 °C; **^1^H NMR (400 MHz, DMSO-*d*_6_)** δ 10.30 (s, 1H, NH amide D_2_O exchangeable), 9.02 (s, 1H, NH D_2_O exchangeable), 8.34 (s,1H, benzothiazole), 8.17 (s, 1H, Ar-H), 7.81 (d, *J* = 7.8, 1H, Ar-H), 7.63 (d, *J* = 8.5 Hz, 1H, benzothiazole), 7.42 (d *, J* = 8.5 Hz, 1H, benzothiazole), 7.33 -7.30 (m, 5H, phenyl), 7.24 (s, 1H, Ar-H), 7.18 (d, *J*= 8.2 Hz, 2H, Ar- H), 6.95 (d, *J =* 7.7 Hz, 1H, Ar-H), 3.64 (s, 6H, -N**-CH_2_**-CH_2_-& piperazine), 3.24 (s, 4H, piperazine), 2.96 (t, 2H, J = 6.8 Hz, -**CH_2_**-Ar); **MS**: (M.wt.: 675.58) : *m/z*, 677.12 [M+2, (27.3%)], 675.13 [M+, (37%)], 647.13 (100%); **Anal.** Calcd for C_33_H_28_Cl_2_N_6_O_4_S: C, 58.67; H, 4.18; N, 12.44; Found: C, 58.65; H, 4.20; N, 12.47.

***N*-(6-(4-Benzhydrylpiperazine-1-carbonyl)benzo[*d*]thiazol-2-yl)-3-nitro-(phenethylamino)-benzamide (12c)**:

The titled compound was obtained as yellow solid (0.13 g, 35%); m.p.170-172°C; **^1^H NMR (400 MHz, DMSO-*d*_6_)** δ 10.30 (s, 1H, NH amide D_2_O exchangeable), 9.02 (s, 1H, NH D_2_O exchangeable), 8.30 (s, 1H Ar-H), 8.08 (s, 1H, benzothiazole), 7.56 ( dd, J= 7.9 , 2.0 Hz ,1H, benzothiazole), 7.44 (d, J= 7.4 Hz, 4H, Ar-H), 7.32-7.28 (m, 10 H, benzhydryl), 7.24-7.19 (m, 3H, Ar-H), 7.14 (d, J= 7.9 Hz,1H, Ar-H), 4.35 (s, 1H,CH benzhydryl), 3.64 (t, 2H, J = 6.8 Hz, -N**-CH_2_**-CH_2_-), 3.53 (s, 4H, piperazine), 2.95 (t, 2H, J = 6.8 Hz, -**CH_2_**-Ar), 2.33 (s, 4H, piperazine); **MS:** (M.wt.: 696.82) : *m/z* 698.25 [M+2, (5.9%)], 697.25[M+1, (3.0%)],696.25 (100%); **Anal.** Calcd for C_40_H_36_N_6_O_4_S: C, 68.95; H, 5.21; N, 12.06; Found: C, 68.65; H, 5.20; N, 12.27.

**(*E*)-*N*-(6-(4-Cinnamylpiperazine-1-carbonyl)benzo*[d*]thiazol-2-yl)-3-nitro-4-(phenethyl-amino)benzamide (12d)**

The titled compound was obtained as yellow solid (0.13g , 40%); m.p.244-246°C; **^1^H NMR (400 MHz, DMSO-*d*_6_)** δ 10.30 (s, 1H, NH amide D_2_O exchangeable), 9.00 (s, 1H, NH D_2_O exchangeable), 8.54 (s, 1H, Ar-H), 8.26 (d, J=8.4 Hz, 1H, Ar-H), 8.06 (s, 1H, benzothiazole), 7.78 (d, J = 8.5 Hz, 1H, Ar-H), 7.47-7.39 (m, 3 H, Ar-H), 7.34 -7.30 (m, 6 H, Ar-H), 7.27-7.23 (m, 3 H, Ar-H), 6.58 (d, *J* = 15.8 Hz, 1H, Ar-**CH**=CH-CH_2_-), 6.30 (dt, *J* = 15.8, 6.5 Hz , 1H, Ar-CH=**CH**-CH_2_-), 3.74 (t, 2H, *J* = 6.8 Hz, -N**-CH_2_**-CH_2_-), 3.53 (s, 4H, piperazine), 3.17(d, *J* = 7.06 Hz, 2H, -**CH_2_**-N), 2.97 (t, 2H, *J* = 6.8 Hz, -**CH_2_**-Ar), 2.47 (s, 4H, piperazine); **MS**: (M.wt.: 646.76) : *m/z* 648.24[M+2, (14%)], 647.24[M+1, (40%)],646.25 (100%); **Anal.** Calcd for C_36_H_34_N_6_O_4_S: C, 66.85; H, 5.30; N, 12.99; Found: C, 66.65; H, 5.20; N, 12.97.

**3-Nitro-4-(phenethylamino)-*N*-(6-(4-(tetrahydrofuran-2-carbonyl)piperazine-1-carbonyl)-benzo[*d*]thiazol-2-yl)benzamide (12e)**:

The titled compound was obtained as yellow solid (0.13 g, 40%); m.p.134-136°C; **^1^H NMR (400 MHz, DMSO-*d*_6_)** δ 10.30 (s, 1H, NH amide D_2_O exchangeable), 9.00 (s, 1H, NH D_2_O exchangeable), 8.79 (s,1H, benzothiazole), 8.44 (s, 1H, Ar-H), 8.14 (d, 1H, *J* = 7.8 Hz, Ar-H), 7.99 (d, *J* = 8.5 Hz, 1H, benzothiazole), 7.89 (d *, J* = 8.5 Hz, 1H, benzothiazole ), 7.38 -7.30 (m, 5H, phenyl), 7.04 (d, *J* = 7.8 Hz,1H, Ar-H), 4.7 (t , *J* = 9.3 Hz, 1H, furoyl H^2^), 3.76 ( t, *J* =14.36 Hz ,2H, furoyl H^5^) , 3.53 (s, 4H, piperazine), 3.68 (t, *J* = 6.8 Hz, 2H, -N**-CH_2_**-CH_2_-), 2.98 (t, *J* = 6.8 Hz, 2H, -**CH_2_**-Ar), 2.47 (s, 4H, piperazine), 2.09-2.04 (m, 4H, furoyl); **MS:** (M.wt.: 628.7) : *m/z* 630.21[M+2, (12.8%)], 629.21[M+1, (38.7%)],628.21 (100%); **Anal.** Calcd for C_32_H_32_N_6_O_6_S: C, 61.13; H, 5.13; N, 13.37; Found: C, 61.15; H, 5.11; N, 13.33.

***N*-(4-((Substituted-benzyl)oxy)phenyl)-2-(3-nitro-4-(phenethylamino)benzamido) benzo[*d*]thiazole-6-carboxamide (13a-d):**

**General Procedure:**

A mixture of 2-(3-nitro-4-(phenethylamino)benzamido)benzo[*d*]thiazole-6-carboxylic acid **(11)** (0.23g , 0.5 mmol), the respective amino compounds **(Ib**, **Id**, **Ie** & **If**) (0.5 mmol), EDC.HCl (0.19g, 1mmol) and DMAP (0.12g, 1mmol) was stirred in dry DMF (5 mL) under N_2_ atmosphere for 72 hours at room temperature. The mixture was added to ice/H_2_O (50 mL) with continued stirring. The resulting solid was filtered, dried and then purified by column chromatography (gradient elution starting from (DCM/MeOH 9.9:0.1) and increasing polarity to (DCM/MeOH 9.7:0.3)) to afford the titled compounds **(13a-d)**.

***N*-(4-((3-Chlorobenzyl)oxy)phenyl)-2-(3-nitro-4-(phenethylamino)benzamido) benzo[*d*]thiazole-6-carboxamide (13a):**

The titled compound was obtained as yellow solid (0.15 g, 45%); m.p. 260-262°C; **^1^H NMR (400 MHz, DMSO-*d*_6_)** δ 13.11 (s, 1H, NH amide D_2_O exchangeable), 10.22 (s, 1H, NH amide D_2_O exchangeable), 9.03 (s, 1H, NH amide D_2_O exchangeable), 8.60 (s, 1H, benzothiazole H^7^), 8.56 (s, 1H, Ar-H), 8.26 (d, 1H, *J* = 9.4 Hz, Ar-H), 8.04 (d, *J* = 9.2 Hz, 1H, benzothiazole), 7.85 (d *, J* = 9.2 , 1H, benzothiazole), 7.72 (d, J= 8.8 Hz, 2H, Ar-H), 7.53 (s, 1H, Ar-H), 7.43 (s, 3 H, Ar-H), 7.35 -7.28 (m, 5H, phenyl), 7.27 (d, *J* = 9 Hz, 1H, Ar-H), 7.03 (d, *J*= 8.8 Hz, 2H, Ar-H), 5.13 (s, 2H, -**CH_2_**-O-Ar), 3.72 (t, *J* = 6.8 Hz, 2H, -N**-CH_2_**-CH_2_-), 2.98 (t, 2H, *J* = 6.8 Hz, -**CH_2_**-Ar); **^13^C NMR (101 MHz, DMSO-*d*_6_)** δ 165.22, 163.86, 161.43, 154.73, 147.55, 140.29, 139.08, 135.78, 133.56, 133.21, 132.79, 131.00, 130.56, 130.23, 129.30, 128.95, 128.49, 128.16, 127.72, 126.91, 126.61, 126.38, 122.53, 122.37, 121.96, 121.64, 120.80, 115.27, 115.21, 114.37, 107.28, 68.90, 44.37, 34.76; **MS:** (M.wt.: 678.17) : *m/z* 680.16 [M+2, (16.2%)], 679.15 [M+1, (46.2%)], 677.16 [M^+^ (100%)]; **Anal.** Calcd for C_36_H_28_ClN_5_O_5_S: C, 63.76; H, 4.16; Cl, 5.23; N, 10.33; Found: C, 63.55; H, 4.12; N, 10.33.

***N*-(4-((4-Bromobenzyl)oxy)phenyl)-2-(3-nitro-4-(phenethylamino)benzamido)-benzo[*d*]thiazole-6-carboxamide (13b):**

The titled compound was obtained as yellow solid (0.15 g, 45%); m.p. 260-262°C; **^1^H NMR (400 MHz, DMSO-*d*_6_)** δ 13.10 (s, 1H, NH amide D_2_O exchangeable), 10.22 (s,1H, NH amide D_2_O exchangeable), 9.03 (s,1H, NH amide D_2_O exchangeable), 8.60 (s,1H, benzothiazole), 8.54 (s, 1H, Ar-H), 8.27 (dd, 1H, *J* = 9.4, 2.7 Hz, Ar-H), 8.04 (d, *J* = 9.2 Hz, 1H, benzothiazole), 7.85 (d *, J* = 9.2, 1H, benzothiazole), 7.71 (d, *J* = 8.8 Hz, 2H, Ar-H), 7.60 (d, *J* = 8.3 Hz, 2H, Ar-H), 7.43 (d, *J* = 8.5 Hz, 2H, Ar-H), 7.34 -7.28 (m, 5H, phenyl), 7.27 (d, *J* = 9 Hz, 1H, Ar-H), 7.03 (d, *J* = 8.9 Hz, 2H, Ar-H), 5.09 (s, 2H, -**CH_2_**-O-Ar), 3.74 (t, *J* = 6.8 Hz, 2H, -N**-CH_2_**-CH_2_-), 2.98 (t, 2H, *J* = 6.8 Hz, -**CH_2_**-Ar); **MS:** (M.wt.: 722.61) : *m/z* 724.10 [M+2, (39.6%)], 723.10 [M+1, (96.3%)], 722.10 [M^+^ (40.2%)]; 721.10 (100%); **Anal.** Calcd for C_36_H_28_BrN_5_O_5_S: C, 59.84; H, 3.91; Br, 11.06; N, 9.69; Found: C, 59.80; H, 4.10; N, 9.65.

***N*-(4-((4-Methylbenzyl)oxy)phenyl)-2-(3-nitro-4-(phenethylamino)benzamido)benzo-[*d*]thiazole-6-carboxamide (13c):**

The titled compound was obtained as yellow solid (0.13 g, 40%); m.p. 138-140 °C; **^1^H NMR (400 MHz, DMSO-*d*_6_)** δ 13.10 (s, 1H, NH amide D_2_O exchangeable), 10.13 (s,1H, NH amide D_2_O exchangeable), 9.03 (s,1H, NH amide D_2_O exchangeable), 8.60 (s,1H, benzothiazole), 8.54 (s, 1H, Ar-H), 8.27 (dd, 1H, *J* = 9.4, 2.7 Hz, Ar-H), 8.04 (d, *J* = 9.2 Hz, 1H, benzothiazole), 7.85 (d *, J* = 9.2, 1H, benzothiazole), 7.71 (d, *J* = 8.8 Hz, 2H, Ar-H), 7.60 (d, *J* = 8.3 Hz, 2H, Ar-H), 7.43 (d, *J* = 8.5 Hz, 2H, Ar-H), 7.34 -7.28 (m, 5H, phenyl), 7.27 (d, *J* = 9 Hz, 1H, Ar-H), 7.03 (d, *J* = 8.9 Hz, 2H, Ar-H), 5.05 (s, 2H, -**CH_2_**-O-Ar), 3.71 (t, *J* = 6.8 Hz, 2H, -N**-CH_2_**-CH_2_-), 2.98 (t, *J* = 6.8 Hz, 2H, -**CH_2_**-Ar), 2.31 (s, 3H, CH_3_); **^13^C NMR (101 MHz, DMSO-*d*_6_)** δ 165.22, 163.50, 155.07, 146.87, 140.29, 139.14, 137.48, 135.59, 133.56, 133.21, 132.74, 131.00, 130.78, 129.42, 129.29, 128.96, 128.49, 128.23, 128.16, 127.72, 126.91, 126.61, 126.38, 122.52, 122.37, 121.96, 121.61, 120.80, 115.27, 115.20, 114.91, 114.37, 107.28, 69.74, 44.34, 34.77, 21.23; **MS:** (M.wt.: 657.74) : *m/z* 660.21 [M+2, (1.9 %)], 659.20 [M+1, (5.3%)], 658.21 [M^+^ (44.2%)]; 657.21 (100%); **Anal.** Calcd for C_37_H_31_N_5_O_5_S: C, 67.56; H, 4.75; N, 10.65; Found: C, 67.60; H, 4.70; N, 10.65.

***N*-(4-((4-Trifluoromethylbenzyl)oxy)phenyl)-2-(3-nitro-4-(phenethylamino)benzamido)-benzo[*d*]thiazole-6-carboxamide (13d):**

The titled compound was obtained as yellow solid (0.16 g, 45%); m.p 218 -220 °C; **^1^H NMR (400 MHz, DMSO-*d*_6_)** δ 13.10 (s, 1H, NH amide D_2_O exchangeable),10.17 (s,1H, NH amide D_2_O exchangeable), 9.03 (s,1H, NH amide D_2_O exchangeable), 8.60 (s,1H, benzothiazole), 8.54 (s, 1H, Ar-H), 8.27 (dd, 1H, *J* = 9.4, 2.7 Hz, Ar-H), 8.04 (d, *J* = 9.2 Hz, 1H, benzothiazole), 7.85 (d *, J* = 9.2, 1H, benzothiazole), 7.71 (d, *J* = 8.8 Hz, 2H, Ar-H), 7.60 (d, *J* = 8.3 Hz, 2H, Ar-H), 7.43 (d, *J* = 8.5 Hz, 2H, Ar-H), 7.34 -7.28 (m, 5H, Ar-H), 7.27 (d, *J* = 9 Hz, 1H, Ar-H), 7.03 (d, *J* = 8.9 Hz, 2H, Ar-H), 5.05 (s, 2H, -**CH_2_**-O-Ar), 3.71 (t, *J* = 6.8 Hz, 2H, -N**-CH_2_**-CH_2_-), 2.98 (t, 2H, *J* = 6.8 Hz, -**CH_2_**-Ar); **^13^C NMR (101 MHz, DMSO-*d*_6_)** δ 165.22, 163.50, 155.07, 146.87, 142.89, 139.28, 137.48, 135.59, 133.56, 133.21, 132.74, 131.00, 130.83, 129.42, 129.29, 128.95, 128.44, 128.23, 128.16, 127.72, 126.91, 126.61, 125.80, 122.53, 122.37, 121.96, 121.61, 120.80, 115.24, 115.20, 114.93, 114.37, 93.00, 77.57 , 69.03, 44.35, 34.79; **MS:** (M.wt.: 711.71) : *m/z* 713.18 [M+2, (10 %)], 712.18 [M+1, (41.2%)], 711.18 (100%); **Anal.** Calcd for C_37_H_28_F_3_N_5_O_5_S: C, 62.44; H, 3.97; F, 8.01; N, 9.84; Found: C, 62.45; H, 4.10; N, 9.75.

***N*-(4-(Substituted-phenoxy)phenyl)-2-(3-nitro-4-(phenethylamino)benzamido) benzo[*d*]thiazole-6-carboxamide (14a,b):**

**General Procedure:**

A mixture of 2-(3-nitro-4-(phenethylamino)benzamido)benzo[*d*]thiazole-6-carboxylic acid **(11)** (0.23g , 0.5 mmol), the respective amino compounds **(IIa,b)** (0.5 mmol), EDC.HCl (0.19g, 1mmol) and DMAP (0.12g, 1mmol) was stirred in dry DMF (5 mL) under N_2_ atmosphere for 72 hours at room temperature. The mixture was added to ice/H_2_O (50 mL) with continued stirring. The resulting solid was filtered, dried and then purified by column chromatography (gradient elution starting from (DCM/MeOH 9.9:0.1) and increasing polarity to (DCM/MeOH 9.7:0.3)) to afford the titled compounds **(14a,b)**.

***N*-(4-(4-Chloro-3-fluorophenoxy)phenyl)-2-(3-nitro-4-(phenethylamino)benzamido)-benzo[*d*]thiazole-6-carboxamide (14a):**

The titled compound was obtained as yellow solid (0.17 g, 50%); m.p. 196 -198°C; **^1^H NMR (400 MHz, DMSO-*d*_6_)** δ 10.32 (s, 1H, NH amide D_2_O exchangeable), 8.80 (s,1H, benzothiazole), 8.45 (s, 1H, Ar-H), 8.14 (d, *J* = 10.6 Hz, 1H, Ar-H), 7.83 (d, *J* = 8.9 Hz, 2H, Ar-H), 7.57 (t, *J* = 8.8 Hz, 1H, Ar-H), 7.34 -7.28 (m, 5H, Ar-H), 7.25 (d, *J* = 9.0 Hz, 2H, Ar-H), 7.13 (d, *J* = 8.7 Hz, 2H, Ar-H), 7.08 (d, *J*= 9.0 Hz, 2H, Ar-H) 6.85 (d, *J* = 10.4 Hz, 1H, Ar-H), 3.72 (t, *J* = 6.8 Hz, 2H, -N**-CH_2_**-CH_2_-), 2.98 (t, *J* = 6.8 Hz, 2H, -**CH_2_**-Ar); **MS:** (M.wt.: 682.12) : *m/z* 684.12 [M+2, (16.4 %)], 683.12 [M+1, (46.2%)], 681.12 (100%); **Anal.** Calcd for C_35_H_25_ClFN_5_O_5_S: C, 61.63; H, 3.69; N, 10.27; Found: C, 61.45; H, 3.70; N, 10.25.

***N*-(4-(4-Bromophenoxy)phenyl)-2-(3-nitro-4-(phenethylamino)benzamidobenzo[*d*]thiazole-6-carboxamide (14b):**

The titled compound was obtained as yellow solid (0.19 g, 55%); m.p. 212 -214°C; **^1^H NMR (400 MHz, DMSO-*d*_6_)** δ 10.29 (s,1H, NH amide D_2_O exchangeable), 8.80 (s,1H, benzothiazole), 8.45 (s, 1H, Ar-H), 8.14 (dd, *J* = 9.4, 2.7 Hz, 1H, Ar-H), 8.04 (d, *J* = 9.2 Hz, 1H, benzothiazole), 7.85 (d, *J* = 9.2 Hz, 1H, benzothiazole), 7.79 (d, *J* = 9.0 Hz, 2H, Ar-H), 7.55 (d, *J* = 8.9 Hz, 2H, Ar-H), 7.34 -7.28 (m, 5H, phenyl), 7.25 (d, *J* = 9.2 Hz, 1H, Ar-H) 7.06 (d, *J* = 9.0 Hz, 2H, Ar-H), 6.96 (d, *J* = 8.9 Hz, 2H, Ar-H), 3.72 (t, *J* = 6.8 Hz, 2H, -N**-CH_2_**-CH_2_-), 2.98 (t, 2H, *J* = 6.8 Hz, -**CH_2_**-Ar); **MS:** (M.wt.: 708.58) : *m/z* 711.08 [M+2, (13.6%)], 710.09 [M+1, (40.2%)], 709.08 (100%); **Anal.** Calcd for C_35_H_26_BrN_5_O_5_S: C, 59.33; H, 3.70; N, 9.88; Found: C, 59.42; H, 3.70; N, 9.85.

***N*-(6-(3-(Substituted-phenyl)ureido)benzo[*d*]thiazol-2-yl)naphthalene-2-sulfonamide (17a-j):**

**General Procedure:**

To a solution of *N*-(6-aminobenzo[*d*]thiazol-2-yl)naphthalene-2-sulfonamide **(16)** (0.18 g, 0.5 mmol) in THF (5 mL), the respective substituted phenyl isocyanate (1mmol) was added at 0^o^C. The reaction mixture was stirred for 24 hours at room temperature. The resulting precipitate was filtered, washed thoroughly with THF, allowed to dry and then purified by column chromatography (gradient elution starting from (DCM/MeOH 9.9:0.1) and increasing polarity to (DCM/MeOH 9.7:0.3)) to afford the titled compounds **(17a-j)**.

***N*-(6-(3-Phenylureido)benzo[*d*]thiazol-2-yl)naphthalene-2-sulfonamide(17a):**

The titled compound was obtained as white solid (0.06 g, 40 %); m.p. ≥ 300 °C; **1H NMR (400 MHz, DMSO-*d*_6_)** δ 13.17 (s, 1H, 8.82 NH sulfonamide D_2_O exchangeable), 8.96 (s, 1H NH urea D_2_O exchangeable ), 8.83 (s, 1H NH urea D_2_O exchangeable ), 8.52 (s, 1H, naphthyl H^1^), 8.17 (d, *J* = 7.6 Hz, 1H, naphthyl), 8.09 (d, *J* = 8.5 Hz, 1H, benzothiazole), 8.02 (d, *J* = 7.6 Hz, 1H, naphthyl), 7.95 (s, 1H, benzothiazole), 7.86 (d, *J* = 8.5 Hz, 1H, benzothiazole), 7.70 -7.63(m, 2H, naphthyl), 7.45 (d, *J* = 7.7 Hz, 1H, naphthyl), 7.39 (d, *J* = 7.7 Hz, 1H, naphthyl), 7.26 (dt, *J* = 13.9, 7.2 Hz, 4H, Ar-H), 6.97 (t, *J* = 7.2 Hz, 1H, Ar-H ); **MS:** (M.wt.: 474 ): *m/z* 476.08 [M+2, (13.7%)], 475.09 [M+1, (29.4%)], 474.08 (100%); **Anal.** Calcd for C_24_H_18_N_4_O_3_S_2_: C, 60.74; H, 3.82; N, 11.81; Found: C, 60.65; H, 3.80; N 11.79.

***N*-(6-(3-(3-Chlorophenyl)ureido)benzo[*d*]thiazol-2-yl)naphthalene-2-sulfonamide (17b):**

The titled compound was obtained as white solid (0.1 g, 60 %); m.p. ≥ 300 °C; **1H NMR (400 MHz, DMSO-*d*_6_)** δ 13.17 (s, 1H, 8.82 NH sulfonamide D_2_O exchangeable), 8.89 (s, 2H, NH urea D_2_O exchangeable), 8.52 (s, 1H, naphthyl H^1^), 8.18 (d, *J* = 7.6 Hz, 1H, naphthyl), 8.10 (d, *J* = 8.7 Hz, 1H, benzothiazole), 8.01 (d, *J* = 7.7 Hz, 1H, naphthyl), 7.95 (s, 1H, benzothiazole), 7.86 (d, *J* = 8.7 Hz, 1H, benzothiazole), 7.71 (s, 1H, Ar-H), 7.70 - 7.66 (m, 2H, naphthyl), 7.42 (d, *J* = 7.7 Hz, 1H, naphthyl), 7.35 – 7.19 (m, 3H, Ar-H), 7.02 (d, *J* = 7.6 Hz, 1H, naphthyl); **MS:** (M.wt.: 509 ): *m/z* 511.04 [M+2, (11.9%)], 510.04 [M+1, (45.7%)], 508.04 (100%); **Anal.** Calcd for C_24_H_17_ClN_4_O_3_S_2_: C, 56.63; H, 3.37; N, 11.01; Found: C, 56.65; H, 3.40; N 11.09.

***N*-(6-(3-(3-Bromophenyl)ureido)benzo[*d*]thiazol-2-yl)naphthalene-2-sulfonamide (17c):**

The titled compound was obtained as off-white solid (0.07 g, 35 %); m.p. ≥ 300 °C; **1H NMR (400 MHz, DMSO-*d*_6_)** δ 13.17 (s, 1H, NH sulfonamide D_2_O exchangeable), 8.90 (d, *J* = 6.2 Hz, 2H, NH urea D_2_O exchangeable), 8.51 (s, 1H, naphthyl H^1^), 8.17 (d, *J* = 7.4 Hz, 1H, naphthyl), 8.08 (d, *J* = 8.6 Hz, 1H, benzothiazole), 8.01 (d, *J* = 7.3 Hz, 1H, naphthyl), 7.93 (s, 1H, benzothiazole), 7.86 (d, *J* = 8.6 Hz, 1H, benzothiazole), 7.78 (s, 1H, Ar-H), 7.73 - 7.61 (m, 2H, naphthyl), 7.39 (d, *J* = 9.4 Hz, 1H, Ar-H), 7.30 (d, *J* = 7.2 Hz, 1H, naphthyl), 7.24 (t, *J* = 7.1 Hz, 2H, Ar-H), 7.15 (d, *J* = 7.2 Hz, 1H, naphthyl); **MS:** (M.wt.: 553.45 ): *m/z* 556 [M+2, (3.3%)], 554 [M+1, (4.1%)], 553.99 (100%); **Anal.** Calcd for C_24_H_17_BrN_4_O_3_S_2_: C, 52.08; H, 3.10; N, 10.12; Found: C, 52.05; H, 3.30; N 10.09.

***N*-(6-(3-(3-Methoxyphenyl)ureido)benzo[*d*]thiazol-2-yl)naphthalene-2-sulfonamide (17d):**

The titled compound was obtained as white solid (0.07 g, 40 %); m.p. ≥ 300 °C; **1H NMR (400 MHz, DMSO-*d*_6_)** δ 13.17 (s, 1H, 8.82 NH sulfonamide D_2_O exchangeable), 8.90 (d, *J* = 6.2 Hz, 2H, NH urea D_2_O exchangeable), 8.48 (s, 1H, naphthyl H^1^), 8.15 (d, *J* = 7.1 Hz, 1H, naphthyl), 8.05 (d, *J* = 8.5 Hz, 1H, benzothiazole), 8.01 (d, *J* = 7.2 Hz, 1H, naphthyl), 7.94 (s, 1H, benzothiazole), 7.85 (d, *J* = 8.5 Hz, 1H, , benzothiazole), 7.71 (s, 1H, Ar-H), 7.70 - 7.66 (m, 2H, naphthyl), 7.39 (d, *J* = 7.7 Hz, 1H, naphthyl), 7.20 (t, *J* = 7.1 Hz, 2H, Ar-H), 6.93 (d, *J* = 7.6 Hz, 1H, naphthyl), 6.56 (d, *J* = 7.1 Hz, 1H, Ar-H), 3.73 (s, 3H,O-CH_3_); **MS:** (M.wt.: 504.58 ): *m/z* 507.09 [M+2, (2.7%)], 506.09 [M+1, (14.4%)], 505 [M^+^, (30.50%)], 504.09 (100%); **Anal.** Calcd for C_25_H_20_N_4_O_4_S_2_: C, 59.51; H, 4.00; N, 11.10; Found: C, 59.05; H, 3.95; N 11.09.

***N*-(6-(3-(*m*-Tolyl)ureido)benzo[*d*]thiazol-2-yl)naphthalene-2-sulfonamide (17e):**

The titled compound was obtained as white solid (0.09 g, 50 %); m.p ≥ 300 °C; **1H NMR (400 MHz, DMSO-*d*_6_)** δ 13.17 (s, 1H, NH sulfonamide D_2_O exchangeable), 8.83 (s, 1H, NH D_2_O exchangeable), 8.64 (s, 1H, NH D_2_O exchangeable), 8.52 (s, 1H, naphthyl H^1^), 8.18 (d, *J* = 7.1 Hz, 1H, naphthyl), 8.09 (d, *J* = 8.5 Hz, 1H, benzothiazole), 8.02 (d, *J* = 7.2 Hz, 1H, naphthyl), 7.95 (s, 1H, benzothiazole), 7.86 (d, *J* = 8.5 Hz, 1H, benzothiazole), 7.70 - 7.62 (m, 2H, naphthyl), 7.41 (d, *J* = 8.6 Hz, 1H, naphthyl), 7.30 (s, 1H, Ar-H), 7.23 (d, *J* = 8.5 Hz, 2H, naphthyl, Ar-H), 7.15 (t, *J* = 7.7 Hz, 1H, Ar-H), 6.78 (d, *J* = 7.2 Hz, 1H, Ar-H), 2.28 (s, 3H, CH_3_); **MS:** (M.wt.: 488.58 ): *m/z* 491 [M+2, (7.9%)], 490 [M+1, (14.1%)], 489 [M^+^, (30.50%)], 488 (100%); **Anal.** Calcd for C_25_H_20_N_4_O_3_S_2_: C, 61.46; H, 4.13; N, 11.47; Found: C, 61.35; H, 4.15; N 11.39.

***N*-(6-(3-(4-Bromophenyl)ureido)benzo[*d*]thiazol-2-yl)naphthalene-2-sulfonamide (17f):**

The titled compound was obtained as buff solid (0.11 g, 50 %); m.p. ≥ 300 °C; **1H NMR (400 MHz, DMSO-*d*_6_)** δ 13.18 (s, 1H, NH sulfonamide D_2_O exchangeable), 8.97 (s, 2H, NH urea D_2_O exchangeable), 8.52 (s, 1H, naphthyl H^1^), 8.18 (d, *J* = 7.1 Hz, 1H, naphthyl), 8.09 (d, *J* = 8.5 Hz, 1H, benzothiazole), 8.02 (d, *J* = 7.2 Hz, 1H, naphthyl), 7.95 (s, 1H, benzothiazole), 7.87 (d, *J* = 8.5 Hz, 1H, benzothiazole), 7.73 (d, *J* = 7.1 Hz, 2H, Ar-H), 7.71 - 7.61 (m, 2H, naphthyl), 7.52 (d, *J* = 7.1 Hz, 2H, Ar-H), 7.41 (d, *J* = 8.6 Hz, 1H, naphthyl), 7.24 (d, *J* = 8.6 Hz, 1H, naphthyl); **^13^C NMR (101 MHz, DMSO-*d*_6_)** δ 167.00, 152.91, 151.59, 139.66, 139.54, 136.23, 134.55, 132.13, 131.97, 129.66, 128.99, 128.24, 127.96, 126.62, 126.15, 122.64, 122.59, 120.60, 118.90, 113.70, 113.60, 112.57; **MS:** (M.wt.: 553.45 ): *m/z* 556 [M+2, (13 %)], 555 [M+1, (29%)], 553 [M^+^, (24.6%)], 554 (100%); **Anal.** Calcd for C_24_H_17_BrN_4_O_3_S_2_: C, 52.08; H, 3.10; N, 10.12; Found: C, 52.35; H, 3.15; N 10.39.

***N*-(6-(3-(4-Chloro-3-(trifluoromethyl)phenyl)ureido)benzo[*d*]thiazol-2-yl)naphthalene-2-sulfonamide (17g):**

The titled compound was obtained as buff solid (0.09 g, 40 %); m.p ≥ 300 °C; **1H NMR (400 MHz, DMSO-*d*_6_)** δ 13.14 (s, 1H, NH sulfonamide D_2_O exchangeable), 9.21 (s, 1H, NH urea D_2_O exchangeable), 9.02 (s, 1H, NH urea D_2_O exchangeable), 8.52 (s, 1H, naphthyl H^1^), 8.18 (d, *J* = 7.1 Hz, 1H, naphthyl), 8.13 (s, 1H, Ar-H), 8.10 (d, *J* = 8.5 Hz, 1H, benzothiazole), 8.02 (d, *J* = 7.2 Hz, 1H, naphthyl), 7.97 (s, 1H, benzothiazole), 7.87 (d, *J* = 8.3 Hz, 1H, Ar-H)**,** 7.67 (m, 2H, naphthyl), 7.52 (d, *J* = 8.3 Hz, 1H, Ar-H), 7.43 (d, *J* = 8.6 Hz, 1H, naphthyl), 7.24 (d, *J* = 8.6 Hz, 1H, naphthyl); **MS:** (M.wt.: 577 ): *m/z* 579.03 [M+2, (11.9%)], 578.04 [M+1, (3.6%)], 577.03 [M^+^, (30.20%)], 576.03(100%); **Anal.** Calcd for C_25_H_16_ClF_3_N_4_O_3_S_2_: C, 52.04; H, 2.79; N, 9.71; Found: C, 52.15; H, 2.65; N 9.69.

***N*-(6-(3-(3,4-Dichlorophenyl)ureido)benzo[*d*]thiazol-2-yl)naphthalene-2-sulfonamide (17h):**

The titled compound was obtained as white solid (0.09 g, 50 %); m.p. 290 - 292 °C; **1H NMR (400 MHz, DMSO-*d*_6_)** δ 13.14 (s, 1H, NH sulfonamide D_2_O exchangeable), 9.03 (s, 1H, NH urea D_2_O exchangeable), 8.90 (s, 1H, NH urea D_2_O exchangeable), 8.48 (s, 1H, naphthyl H^1^), 8.14 (dd, *J* = 7.2, 2.4 Hz, 1H, naphthyl), 8.10 (s, 1H, Ar-H), 8.05 (d, *J* = 8.5 Hz, 1H, benzothiazole), 7.99 (d, *J* = 7.2 Hz, 1H, naphthyl), 7.88 (s, 1H, benzothiazole), 7.86 (dd, *J* = 8.7, 1.9 Hz, 1H, Ar-H),7.67–7.61 (m, 2H, naphthyl), 7.51 (d, *J* = 8.8 Hz, 1H, Ar-H), 7.33 (d, *J* = 8.9 Hz, 1H, naphthyl), 7.22 (d, *J* = 8.7 Hz, 1H, naphthyl); **MS:** (M.wt.: 543.44 ): *m/z* 545 [M+2, (21.1%)], 544 [M+1,(73.4%)], 543 [M^+^, (3.1%)], 542 (100%); **Anal.** Calcd for C_24_H_16_Cl_2_N_4_O_3_S_2_: C, 53.04; H, 2.97; N, 10.31; Found: C, 53.15; H, 3.15; N 10.39.

***N*-(6-(3-(4-Chloro-2-methylphenyl)ureido)benzo[*d*]thiazol-2-yl)naphthalene-2-sulfonamide (17i):**

The titled compound was obtained as white solid (0.07 g, 35 %); m.p. 280 -282 °C; **1H NMR (400 MHz, DMSO-*d*_6_)** δ 13.14 (s, 1H, NH sulfonamide D_2_O exchangeable), 8.85 (s, 1H, NH urea D_2_O exchangeable), 8.77 (s, 1H, NH urea D_2_O exchangeable), 8.52 (s, 1H, naphthyl H^1^), 8.19 (d, *J* = 7.1 Hz, 1H, naphthyl), 8.13 (s, 1H, Ar-H), 8.10 (d, *J* = 8.5 Hz, 1H, benzothiazole), 8.03 (d, *J* = 7.2 Hz, 1H, naphthyl), 7.94 (s, 1H, benzothiazole), 7.86 (d, *J* = 8.7 Hz, 1H, Ar-H)**,** 7.70 - 7.63 (m, 2H, naphthyl), 7.41 (d, *J* = 8.6 Hz, 1H, naphthyl), 7.24 (d, *J* = 8.6 Hz, 1H, naphthyl), 7.20 (d, *J* = 8.6 Hz, 1H, Ar-H), 2.27 (s, 3H, CH_3_); **MS:** (M.wt.: 523.06 ): *m/z* 525.06 [M+2, (11.9%)], 524.06 [M+1, (33.5%)], 523.06 [M^+^, (30.50%)], 522.06 (100%); **Anal.** Calcd for C_25_H_19_ClN_4_O_3_S_2_: C, 57.41; H, 3.66; N, 10.71; Found: C, 57.35; H, 3.55; N 10.69.

***N*-(6-(3-(3-Chloro-4-methylphenyl)ureido)benzo[*d*]thiazol-2-yl)naphthalene-2-sulfonamide (17j):**

The titled compound was obtained as off-white solid (0.06 g, 30 %); m.p ≥ 300 °C; **1H NMR (400 MHz, DMSO-*d*_6_)** δ 13.14 (s, 1H, NH sulfonamide D_2_O exchangeable), 8.85 (s, 1H, NH urea D_2_O exchangeable), 8.77 (s, 1H, NH urea D_2_O exchangeable), 8.52 (s, 1H, naphthyl H^1^), 8.19 (d, *J* = 7.1 Hz, 1H, naphthyl), 8.13 (s, 1H, Ar-H), 8.10 (d, *J* = 8.5 Hz, 1H, benzothiazole), 8.03 (d, *J* = 7.2 Hz, 1H, naphthyl), 7.94 (s, 1H, benzothiazole), 7.86 (d, *J* = 8.7 Hz, 1H, Ar-H)**,** 7.69 -7.61(m, 2H, naphthyl), 7.41 (d, *J* = 8.6 Hz, 1H, naphthyl), 7.24 (d, *J* = 8.7 Hz, 1H, naphthyl), 7.20 (d, *J* = 8.6 Hz, 1H, Ar-H), 2.27 (s, 3H, CH_3_); **MS:** (M.wt.: 523.06 ): *m/z* 525.06 [M+2, (11.9%)], 524.06 [M+1, (33.5%)], 523.06 [M^+^, (30.50%)], 522.06 (100%); **Anal.** Calcd for C_25_H_19_ClN_4_O_3_S_2_: C, 57.41; H, 3.66; N, 10.71; Found: C, 57.45; H, 3.75; N 10.79.

# Docking poses and energies of key products

## Table S1. 2D interactions, C-Docker docking scores, and binding interactions of active compounds with > 50 % of BCL-2 inhibition. Figures were generated using Discovery studio 2.

| **ID** | **2D Interaction Diagram** | **C-Docker Interaction Energy & binding interactions** | **3D Docking View** | |
| --- | --- | --- | --- | --- |
| **LEAD (compound 2)** | 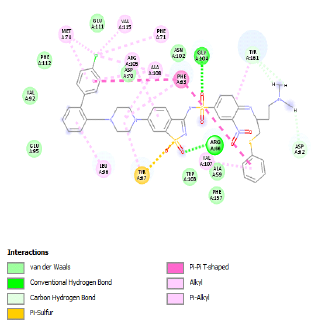 | **-56.038**  **HBA** Gly 104  **HBA** Arg 66  **Pi - Pi** Phe 63  **Pi-Alkyl** Tyr 67  **Pi-Alkyl** Tyr 161  **Pi-Alkyl** Val 107 | 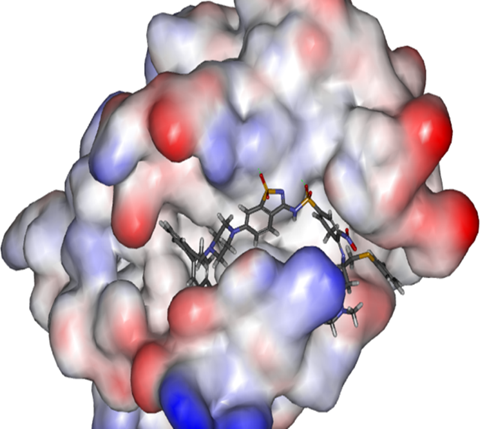 | |
| **7a** | 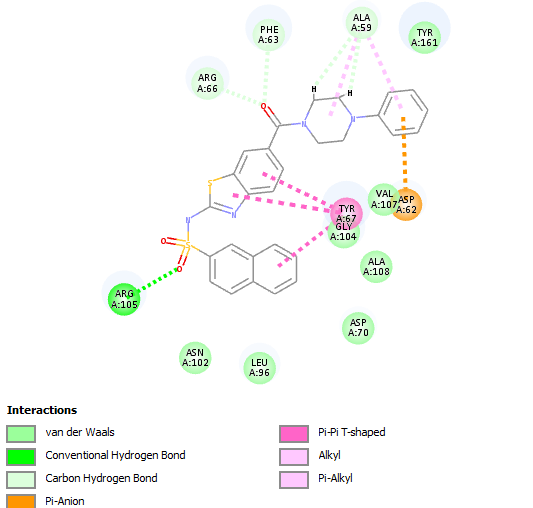 | **-43.36**  **HBA** Arg 105  **Pi – Pi** Tyr 67 | | 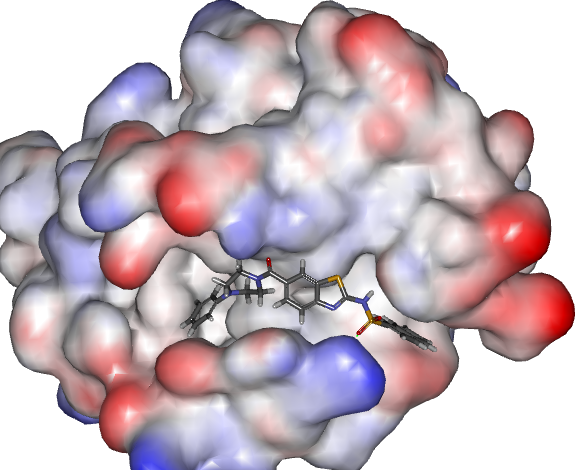 |
| **7b** | 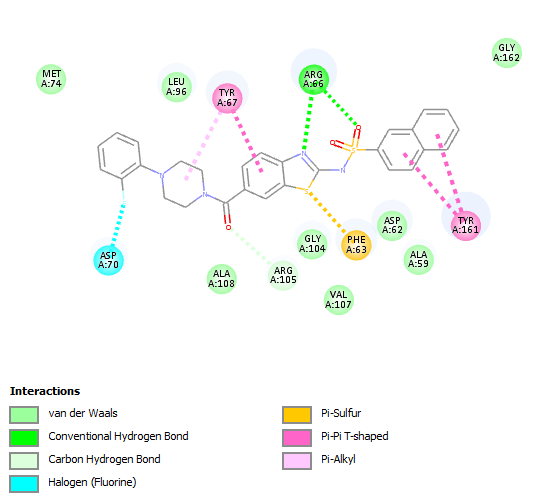 | **-43.33**  **2 HBA** Arg 66  **Pi -sulfur** Phe 63  **Pi-Pi** Tyr 67  **Pi-Pi** Tyr 161  **Halogen (F)** Asp 70 | | 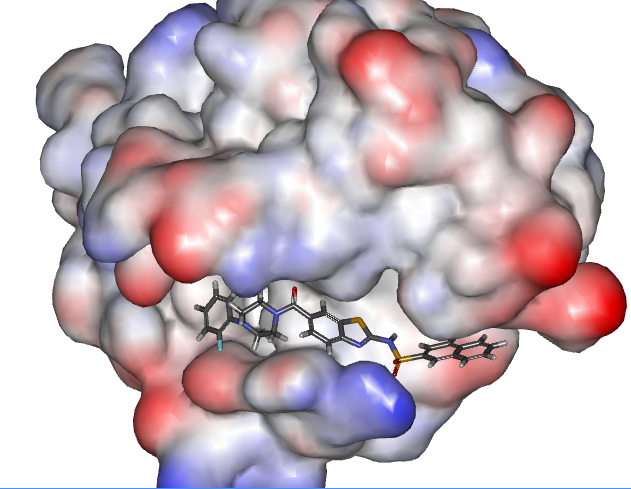 |
| **7c** | **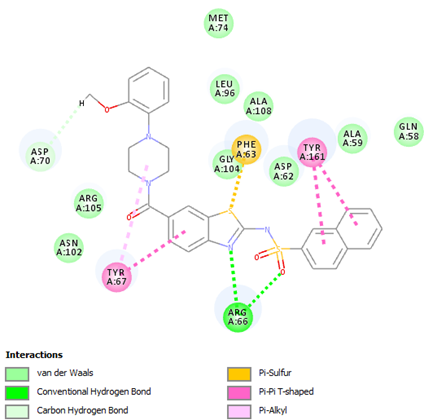** | **-48.74**  **2 HBA** Arg 66  **Pi -sulfur** Phe 63  **Pi-Pi** Tyr 67  **Pi-Pi** Tyr 161 | | 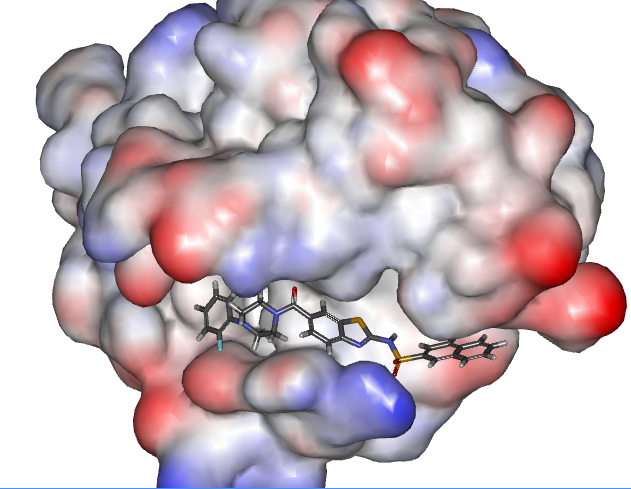 |
| **7d** | 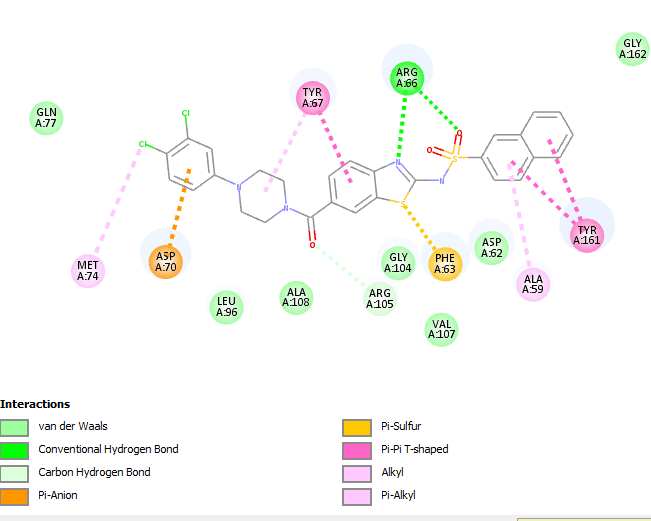 | **-47.02**  **2 HBA** Arg 66  **Pi -sulfur** Phe 63  **Pi-Pi** Tyr 67  **Pi-Pi** Tyr 161 | | 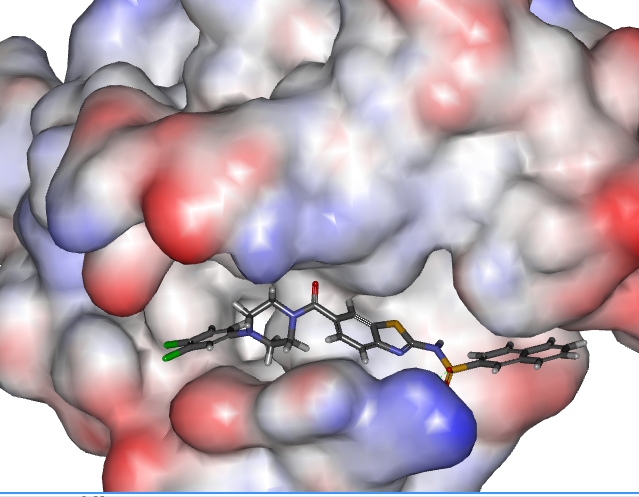 |
| **7e** | 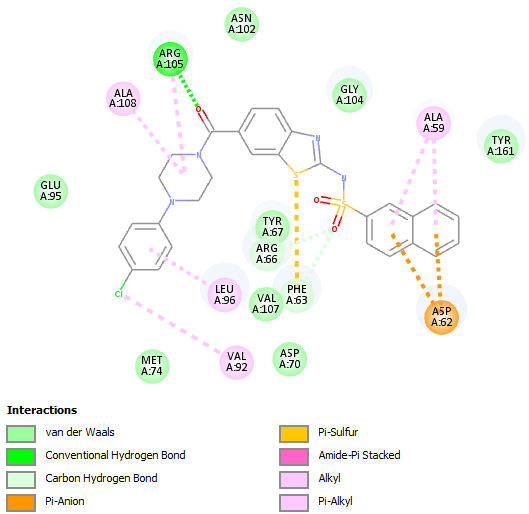 | **-43.76**  **HBA** Arg 105  **Pi -sulfur** Phe 63 | | 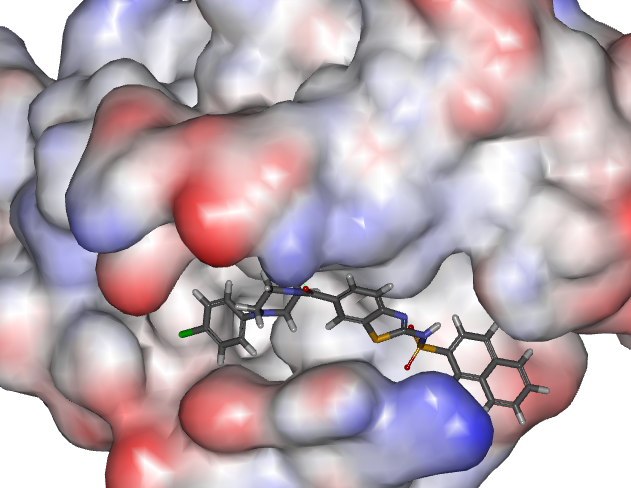 |
| **7f** | 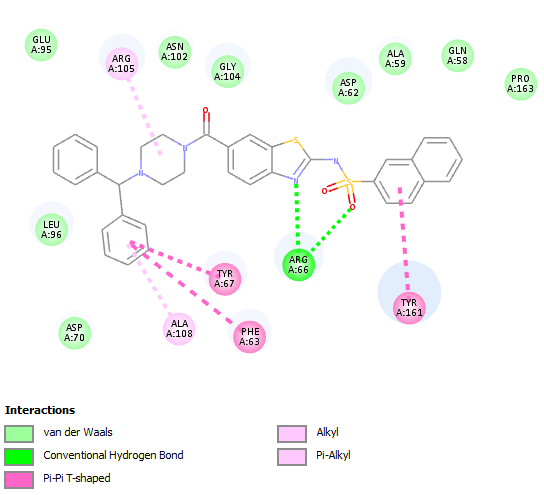 | **-48.98**  **2 HBA** Arg 66  **Pi -Pi** Phe 63  **Pi-Pi** Tyr 67  **Pi-Pi** Tyr 161 | | 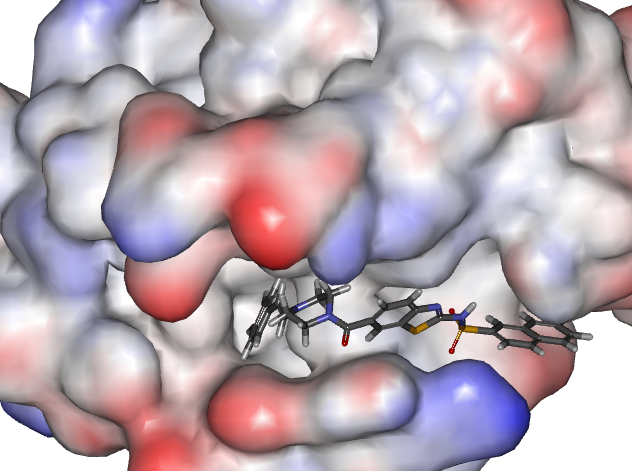 |
| **7g** | **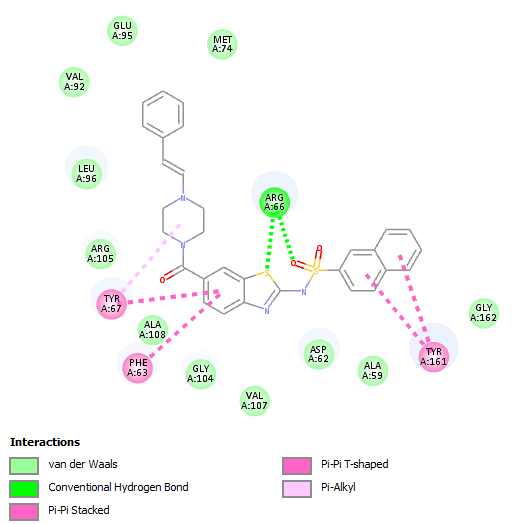** | **-45.16**  **2 HBA** Arg 66  **Pi -Pi** Phe 63  **Pi-Pi** Tyr 67  **Pi-Pi** Tyr 161 | | 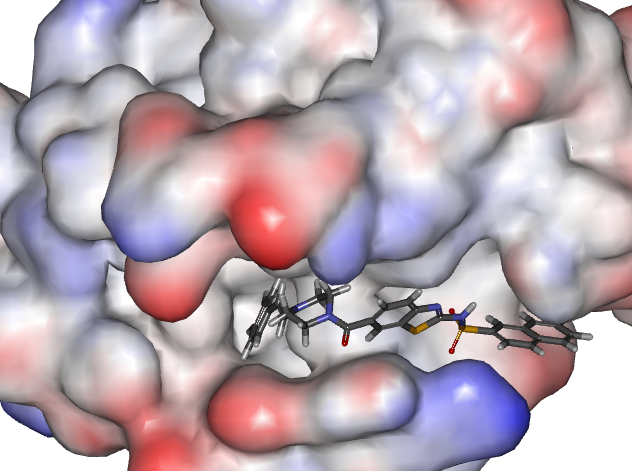 |
| **7h** | 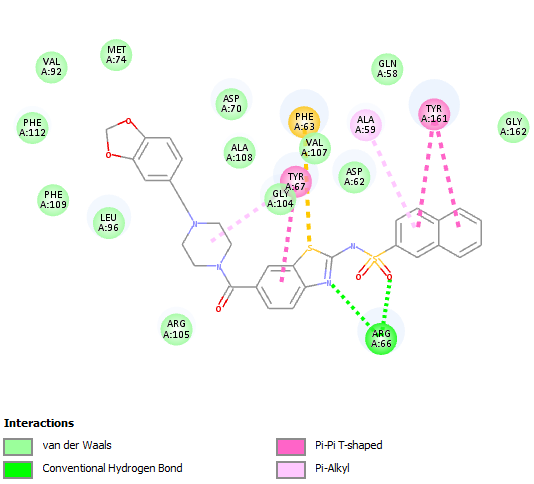 | **-50.63**  **2 HBA** Arg 66  **Pi-Pi** Tyr 67  **Pi-Pi** Tyr 161 | | 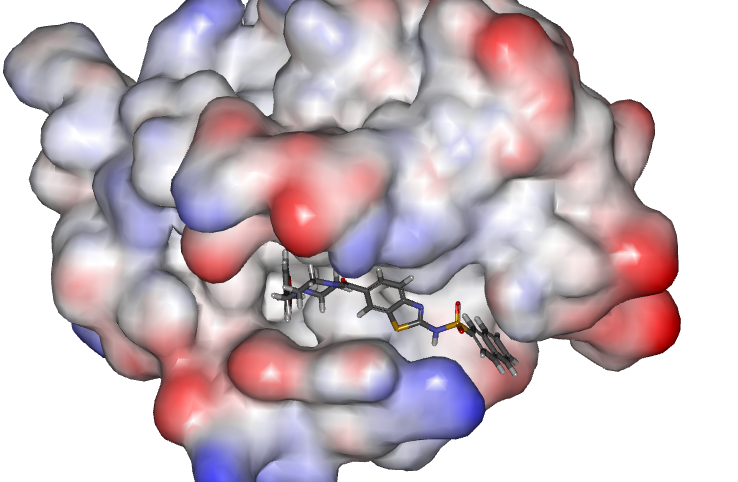 |
| **7i** | 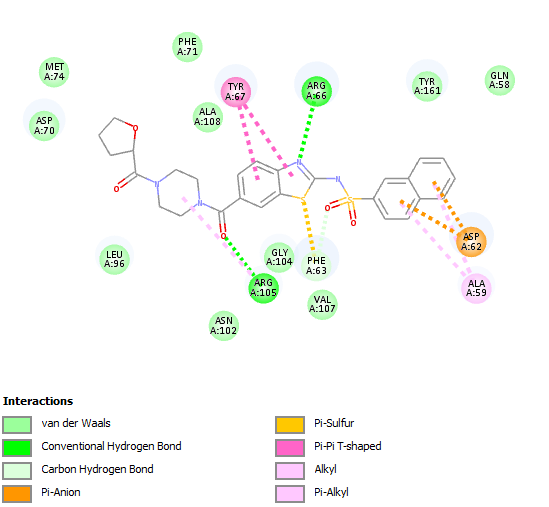 | **-44.72**  **HBA** Arg 66  **HBA** Arg 105  **Pi -sulfur** Phe 63  **Pi-Pi** Tyr 67 | | 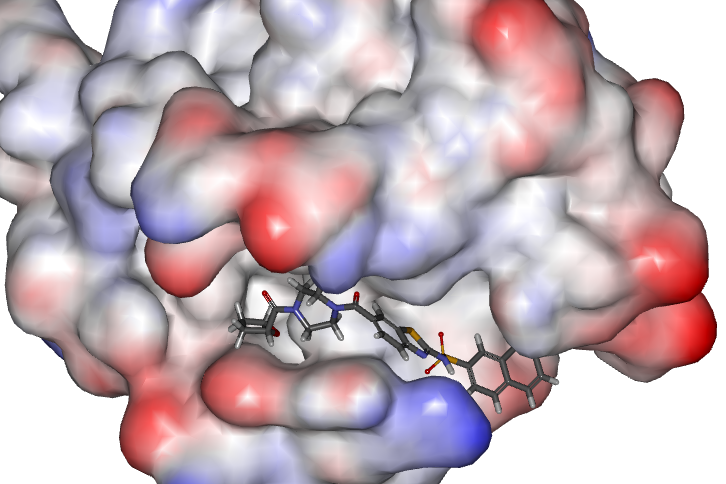 |
| **8a** | 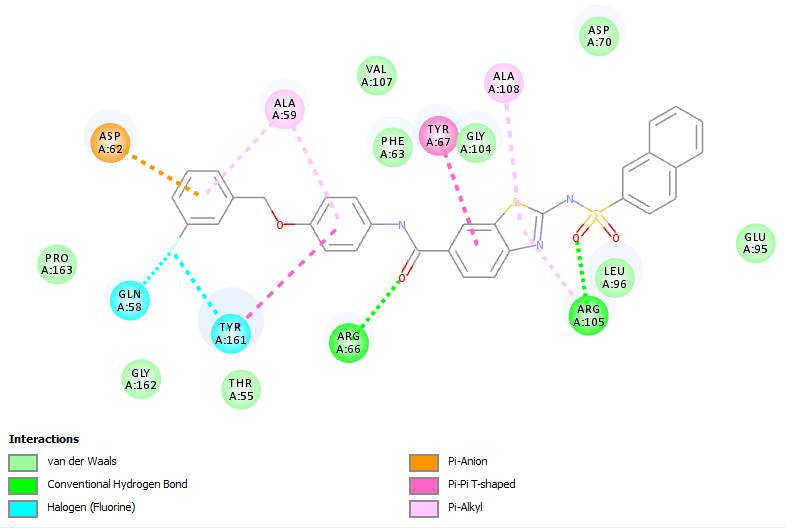 | **-50.19**  **HBA** Arg 66  **HBA** Arg 105  **Pi-Pi** Tyr 67  **Pi-Pi** Tyr 161  **Halogen (F)** Gln58 & Tyr 161 | | 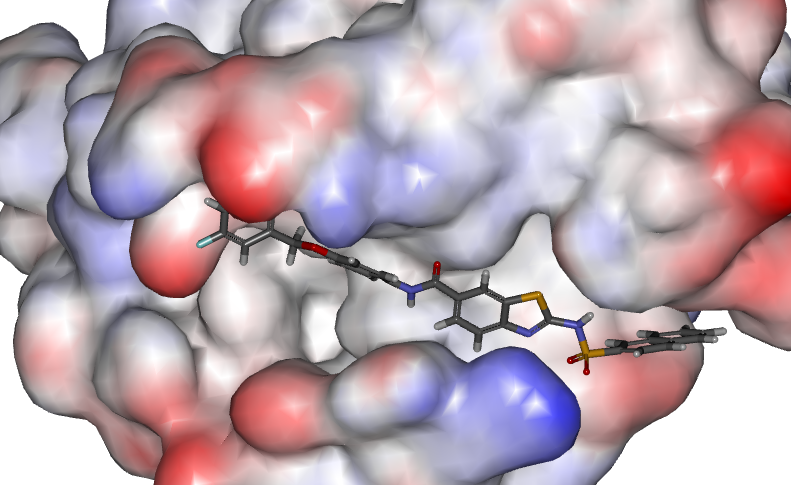 |
| **8b** | 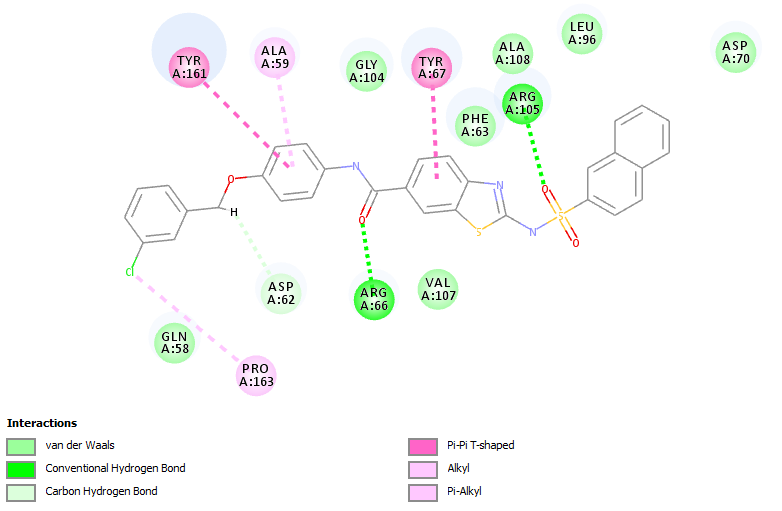 | **-54.84**  **HBA** Arg 66  **HBA** Arg 105  **Pi-Pi** Tyr 67  **Pi-Pi** Tyr 161 | | 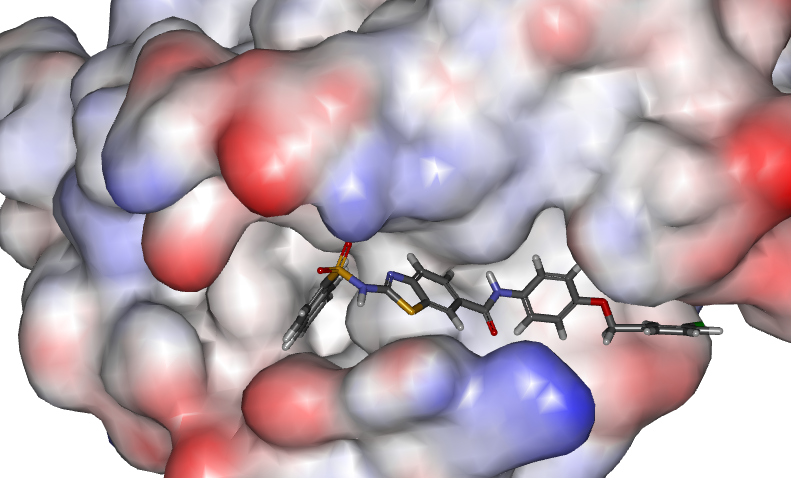 |
| **8c** | 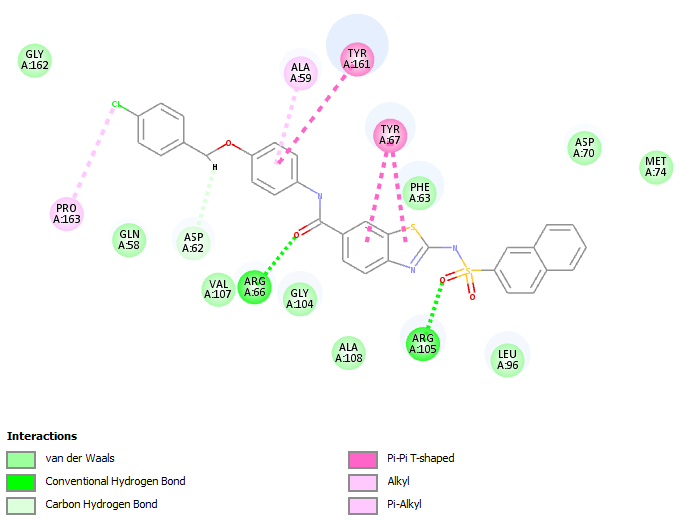 | **-51.64**  **HBA** Arg 66  **HBA** Arg 105  **Pi-Pi** Tyr 67  **Pi-Pi** Tyr 161 | | 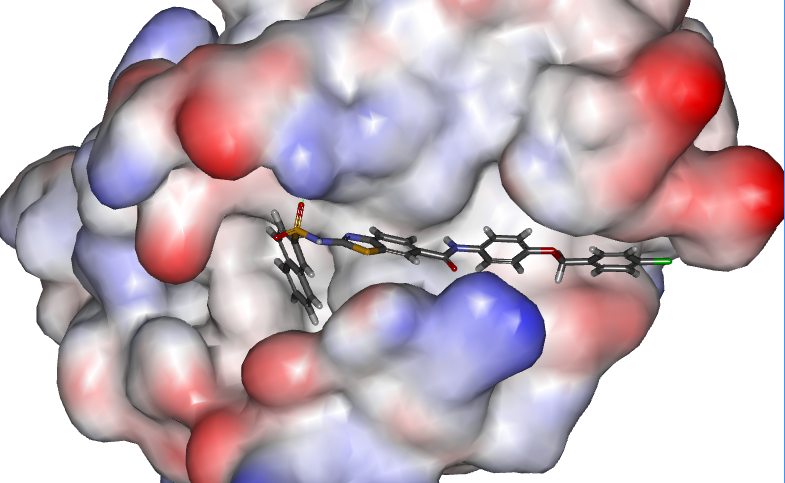 |
| **8d** | 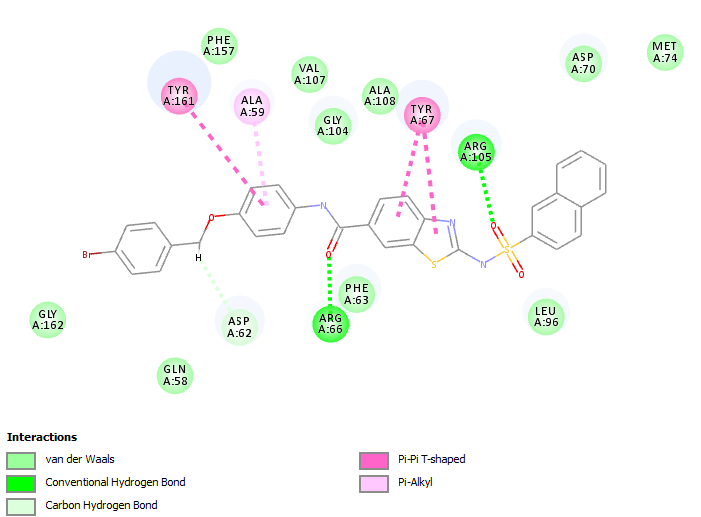 | **-53.08**  **HBA** Arg 66  **HBA** Arg 105  **Pi-Pi** Tyr 67  **Pi-Pi** Tyr 161 | | 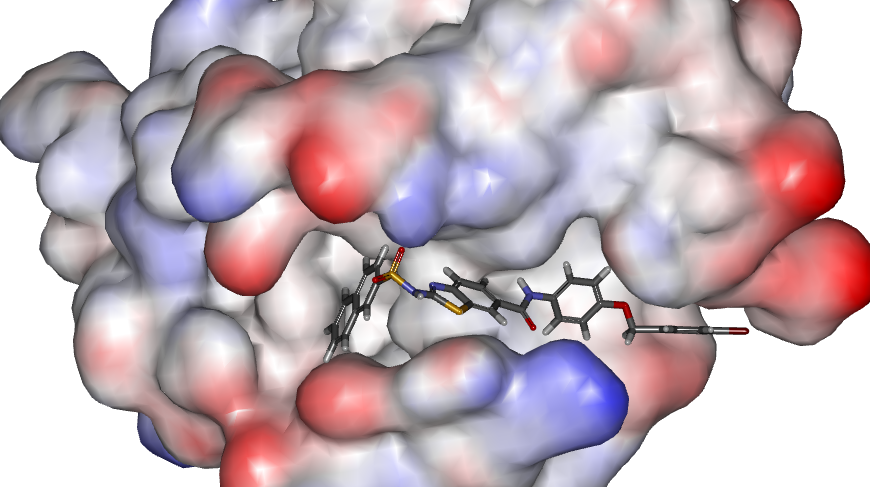 |
| **8e** | 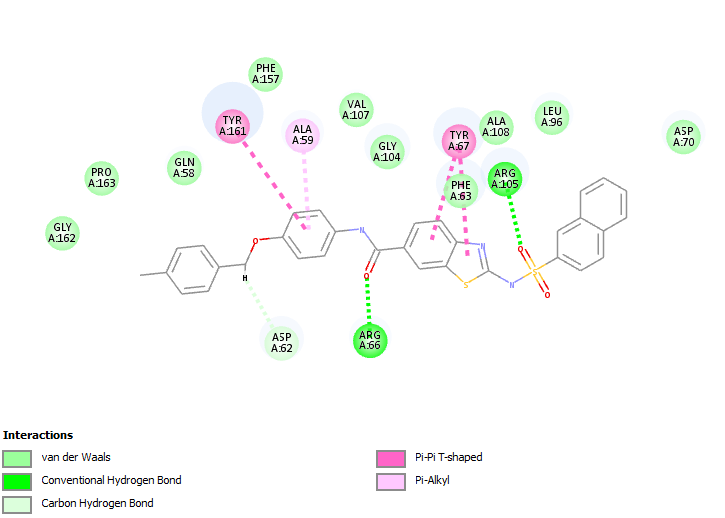 | **-54.59**  **HBA** Arg 66  **HBA** Arg 105  **Pi-Pi** Tyr 67  **Pi-Pi** Tyr 161 | | 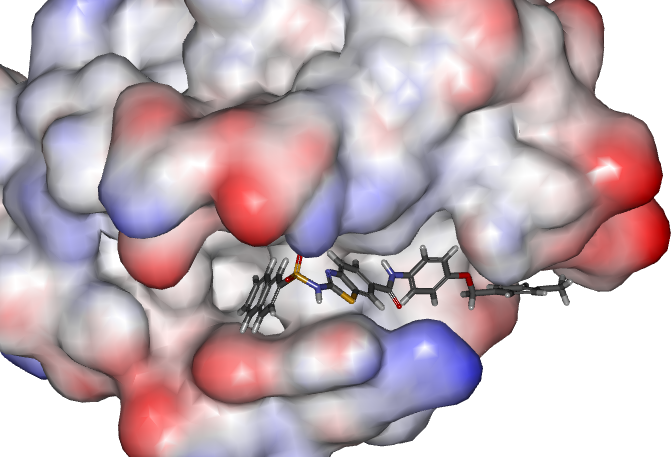 |
| **8f** | 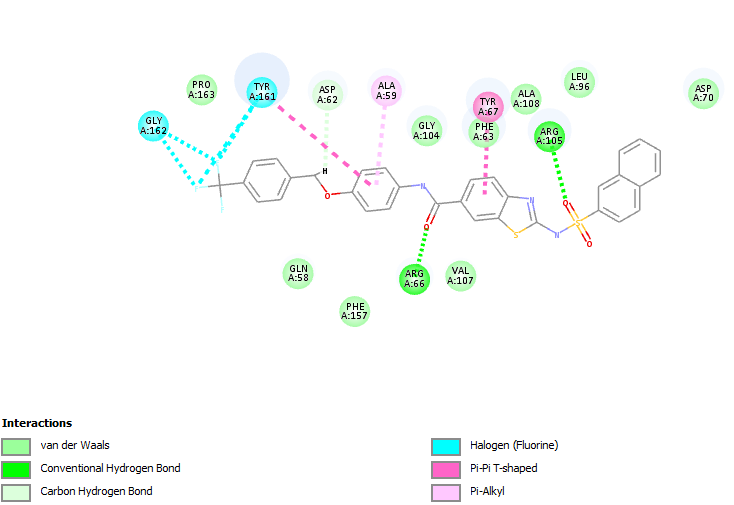 | **-55.18**  **HBA** Arg 66  **HBA** Arg 105  **Pi-Pi** Tyr 67  **Pi-Pi** Tyr 161  **Halogen (F)** Gly162 & Tyr 161 | | 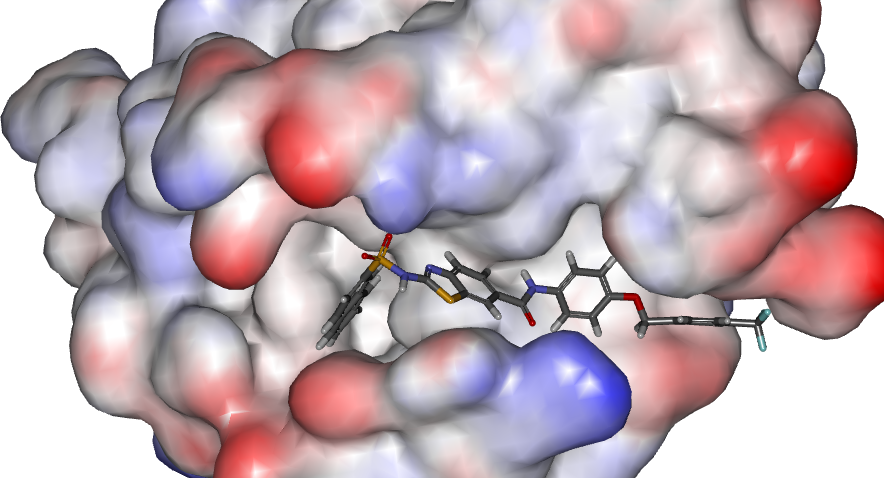 |
| **9a** | 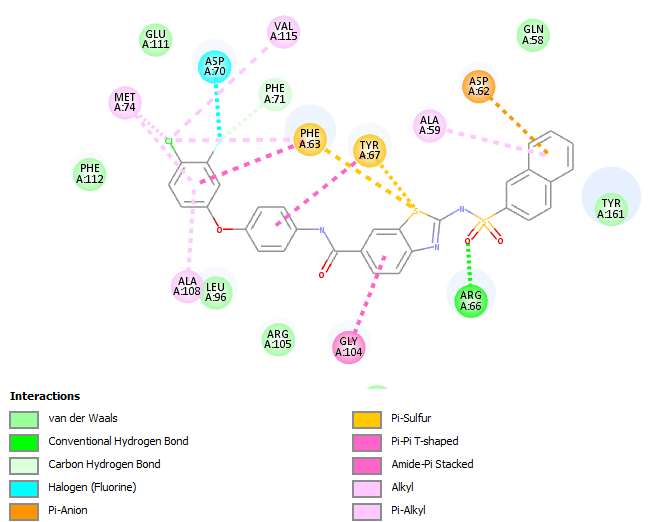 | **-51.31**  **HBA** Arg 66  **Pi-Pi** Gly 104  **Pi-Pi** Tyr 161  **Pi -sulfur** Phe 63  **Pi -sulfur** Tyr 67  **Halogen (F)** Asp70 | | 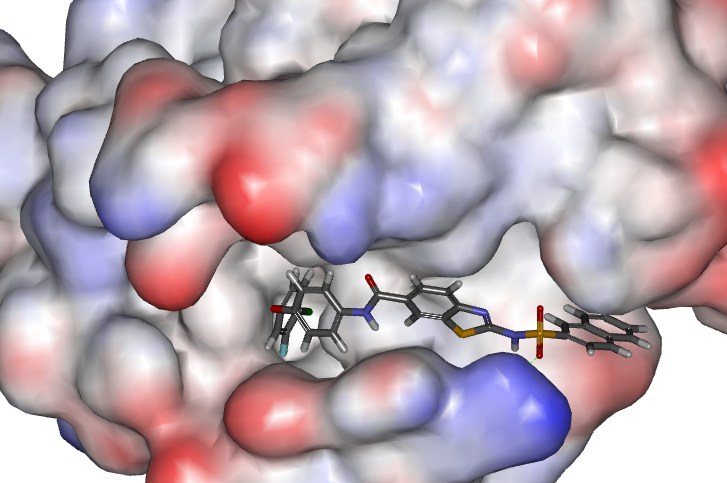 |
| **9b** | 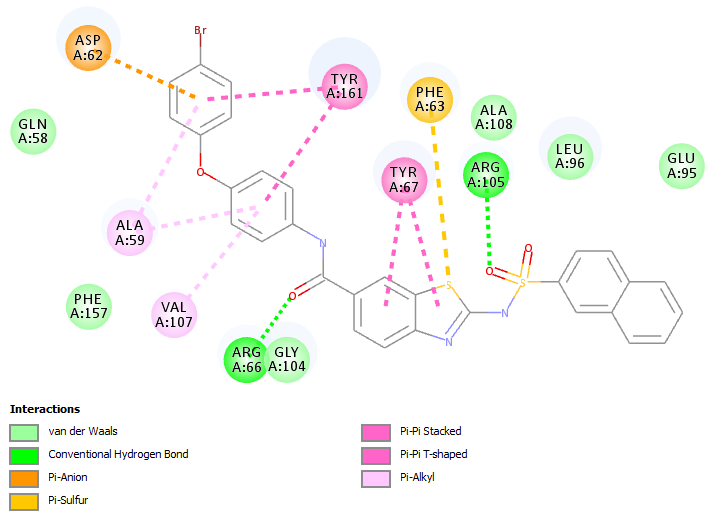 | **-49.52**  **HBA** Arg 66  **HBA** Arg 105  **Pi-Pi** Tyr 67  **Pi-Pi** Tyr 161  **Pi -sulfur** Phe 63 | | 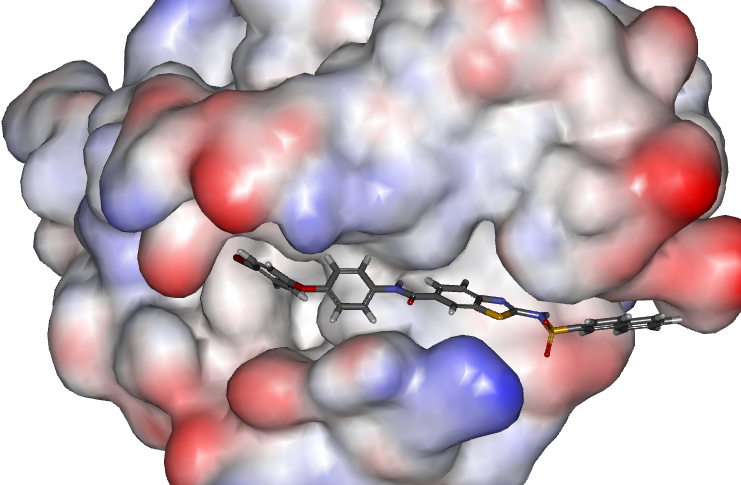 |
| **12a** | 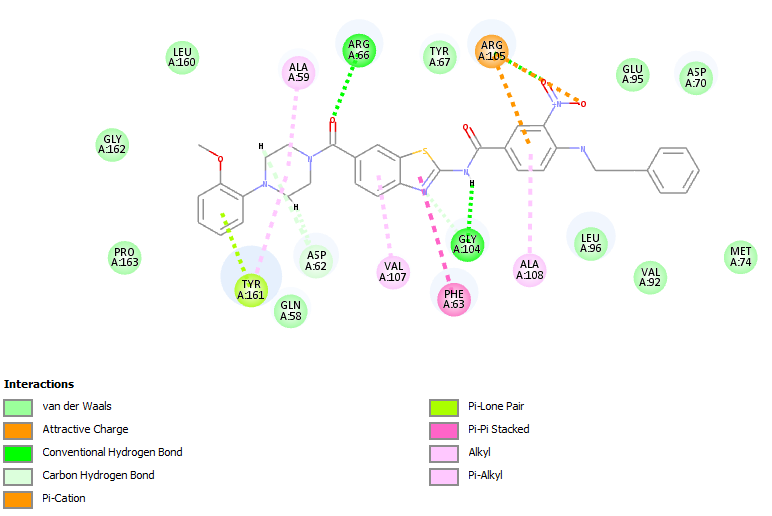 | **-58.07**  **HBA** Arg 66  **HBA** Arg 104  **Pi –Pi** Phe 63 | | 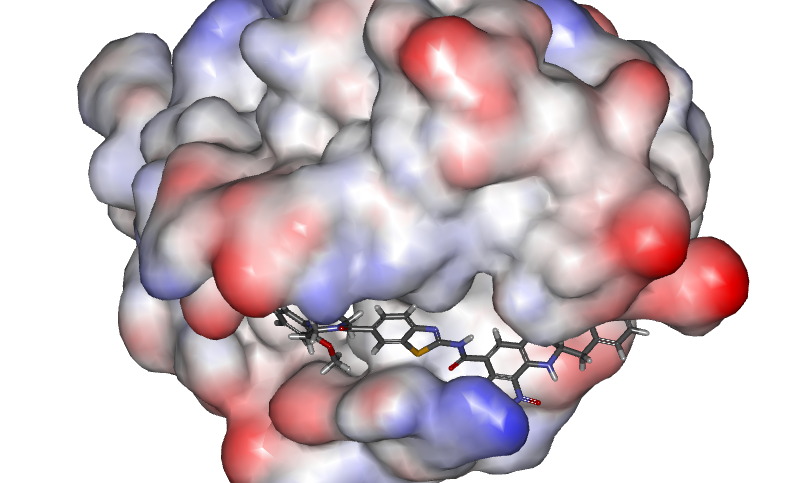 |
| **12b** | 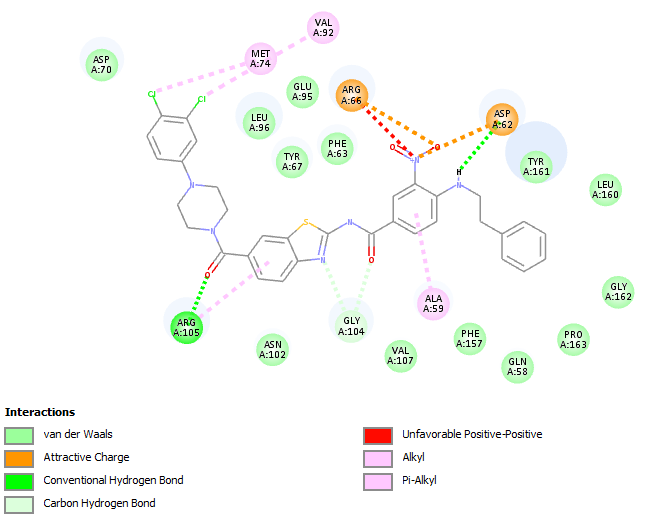 | **-54.23**  **HBA** Arg 105 | | 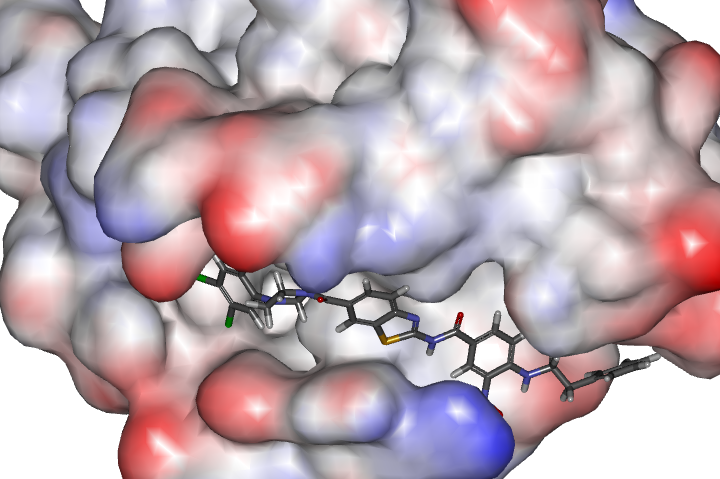 |
| **12c** | 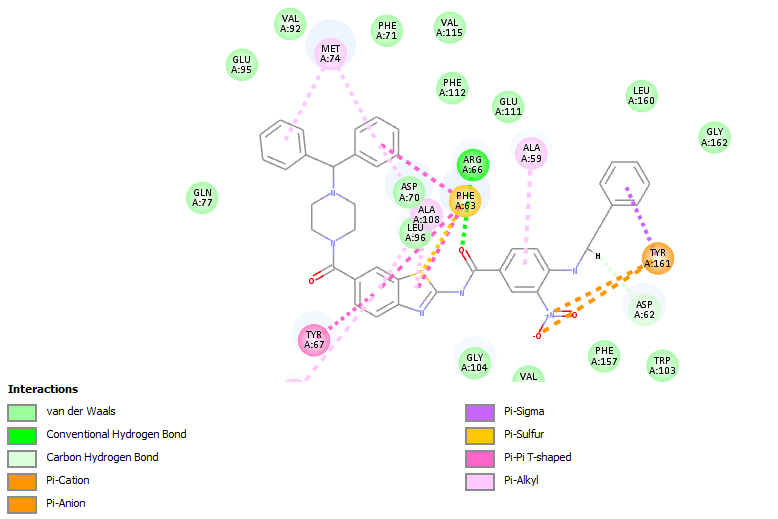 | **-67.57**  **HBA** Arg 66  **Pi-Pi** Tyr 67 | | 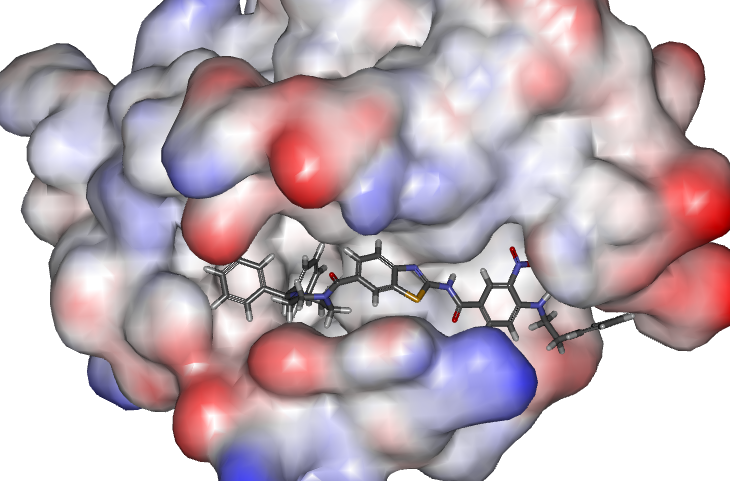 |
| **12d** | 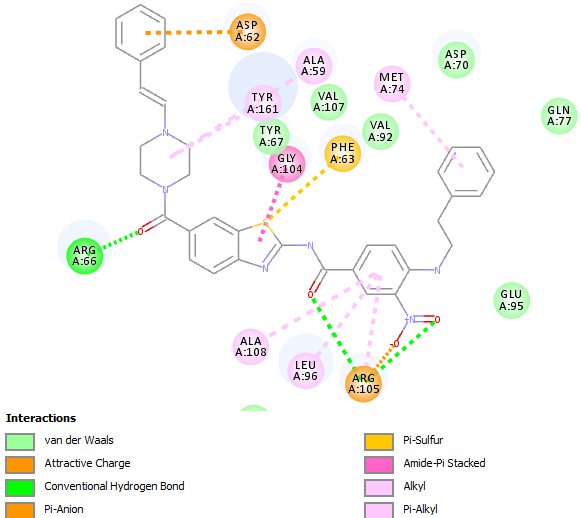 | **-56.56**  **HBA** Arg 66  **HBA** Arg 105  **Pi-Pi** Gly 104 | | 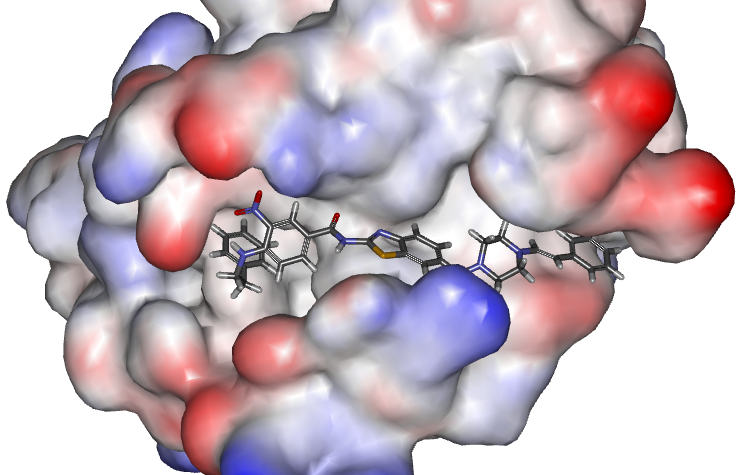 |
| **12e** | 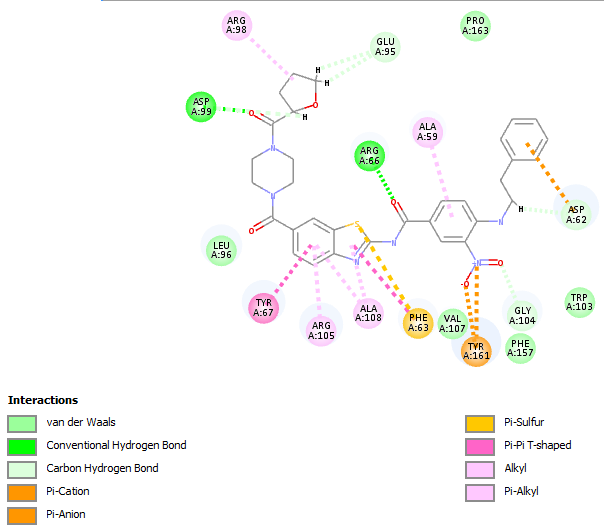 | **-54.20**  **HBA** Arg 66  **HBA** Asp99  **Pi-Pi** Tyr 67 | | 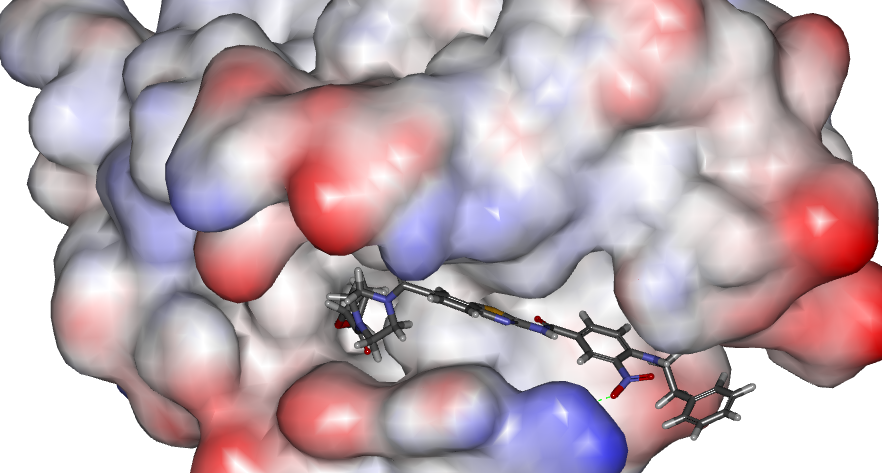 |
| **13a** | 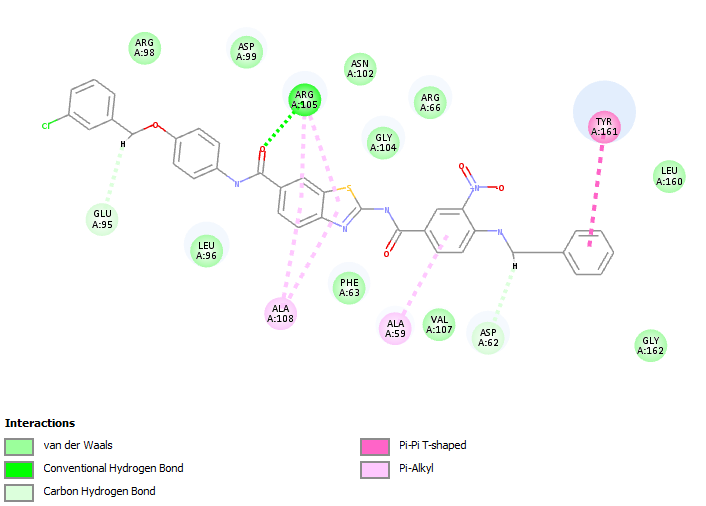 | **-55.70**  **HBA** Arg 105  **Pi-Pi** Tyr 161 | | 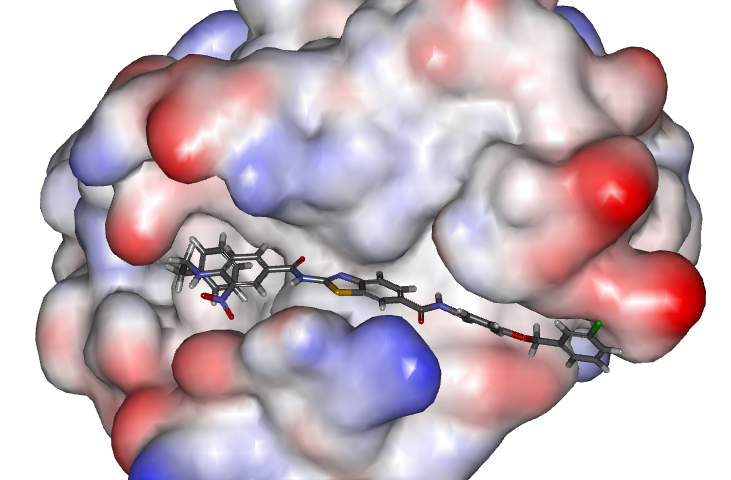 |
| **13b** | 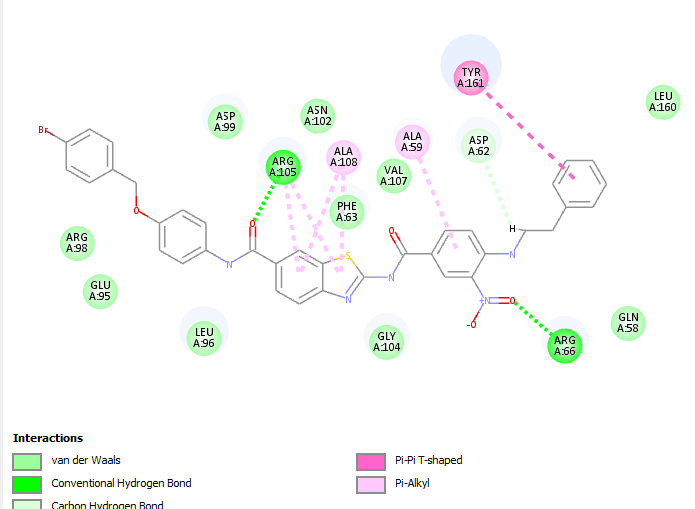 | **-55.83**  **HBA** Arg 66  **HBA** Arg 105  **Pi-Pi** Tyr 161 | | 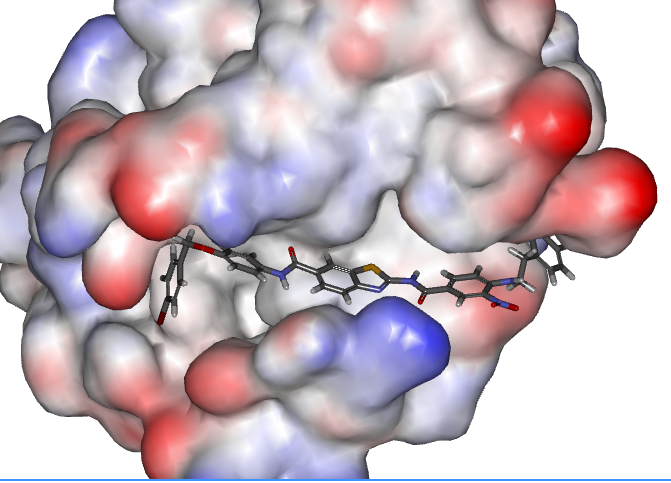 |
| **13c** | 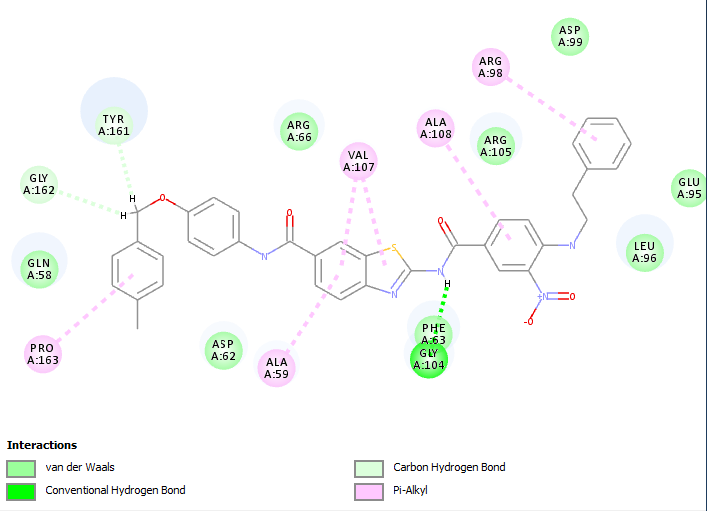 | **-58.05**  **HBA** Gly 104  **Pi-Alkyl** Ala 59  **Pi-Alkyl** Arg 98  **Pi-Alkyl** Val 107  **Pi-Alkyl** Ala 108  **Pi-Alkyl** Pro 163 | | 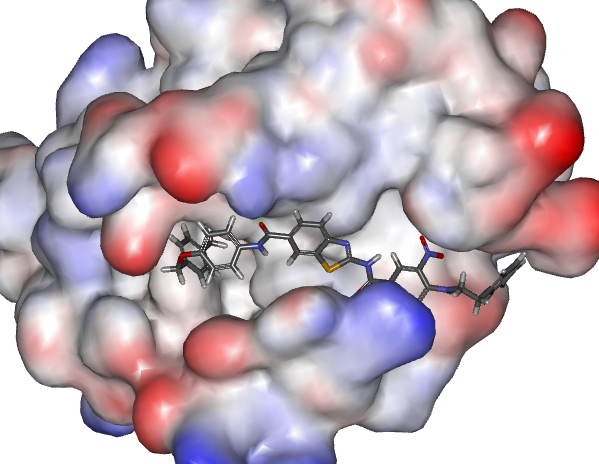 |
| **13d** | 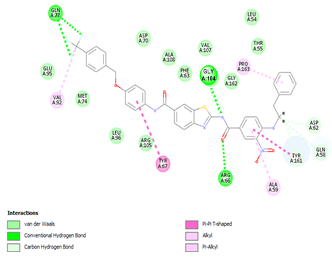 | **-59.15**  **HBA** Gly 104  **Pi-Pi** Tyr 67  **Pi-Pi** Tyr 161  **Pi- Pi-Alkyl** Ala 59  **Pi-Alkyl** Val 92  **Pi-Alkyl** Pro 163 | | 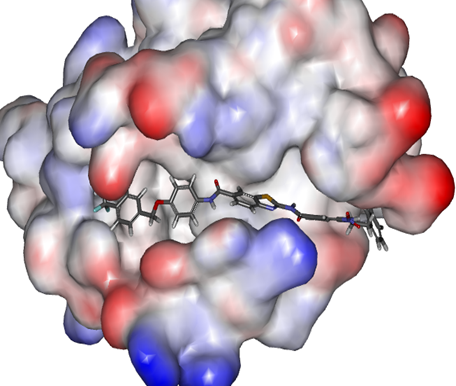 |
| **14a** | 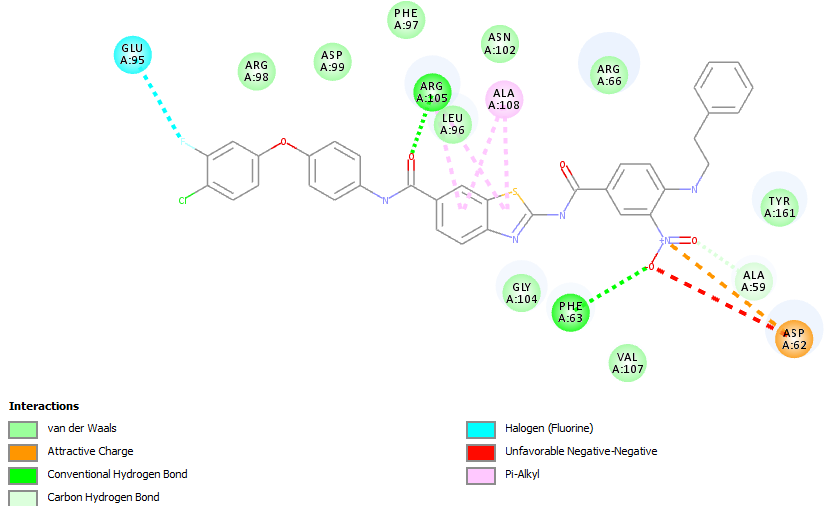 | **-51.19**  **HBA** Arg 105  **HBA** Phe 63  **Pi- alkyl** Ala 108  **Halogen (F)** Glu95 | | 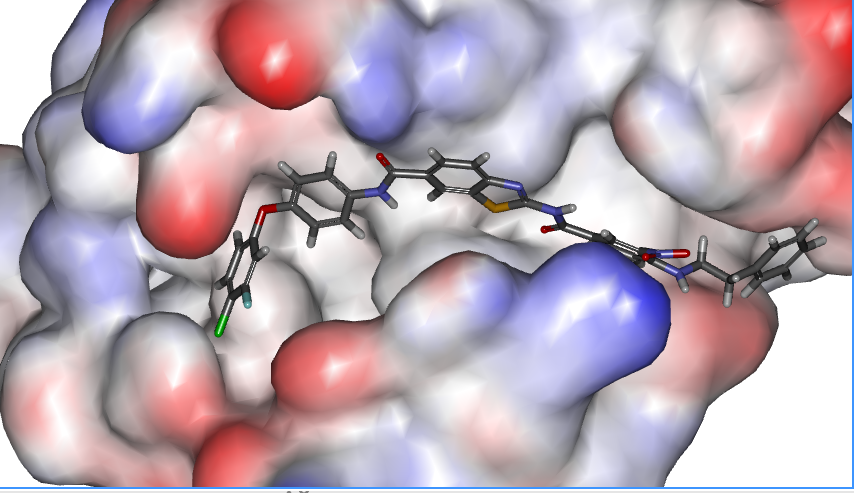 |
| **14b** | 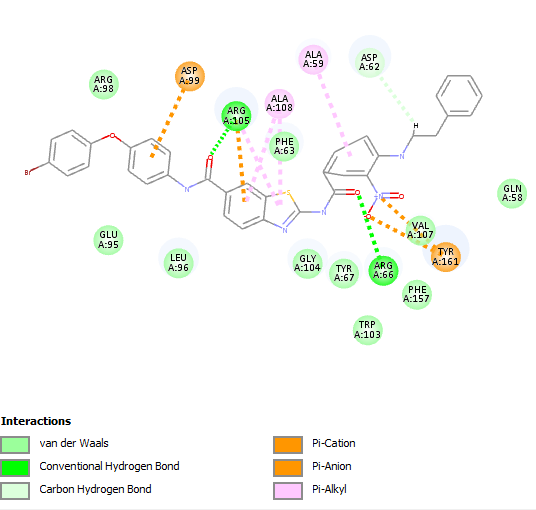 | **-55.11**  **HBA** Arg 66  **HBA** Arg 105  **Pi- alkyl** Ala 59  **Pi- alkyl** Ala 108 | | 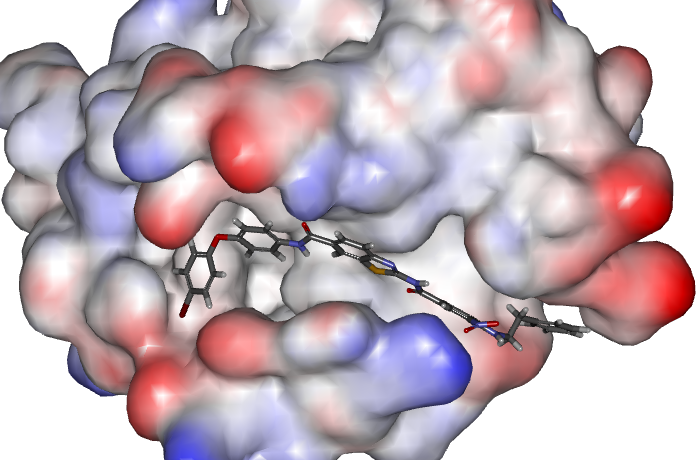 |
| **17a** | 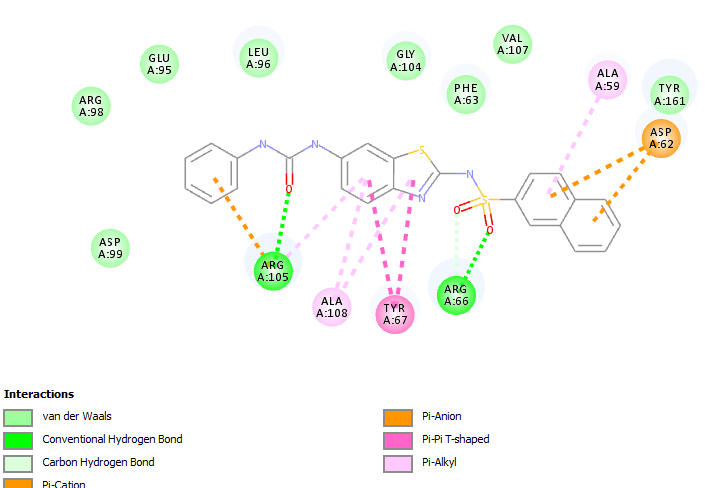 | **-46.49**  **HBA** Arg 66  **HBA** Arg 105  **Pi-Pi** Tyr 67  **Pi- Alkyl** Ala 59  **Pi- Alkyl** Ala 108 | | 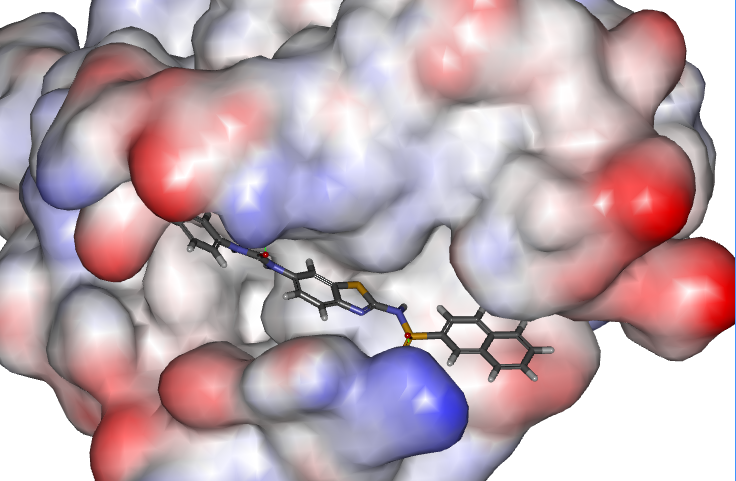 |
| **17b** | 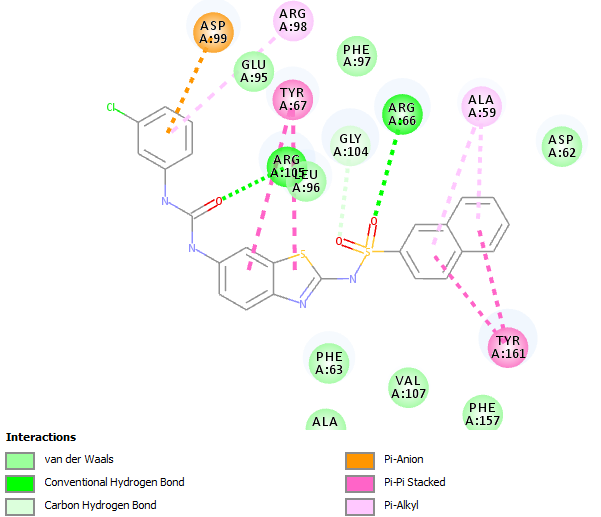 | **-41.09**  **HBA** Arg 66  **HBA** Arg 105  **Pi-Pi** Tyr 67  **Pi-Pi** Tyr 161  **Pi- Alkyl** Ala 59  **Pi- Alkyl** Arg 98 | | 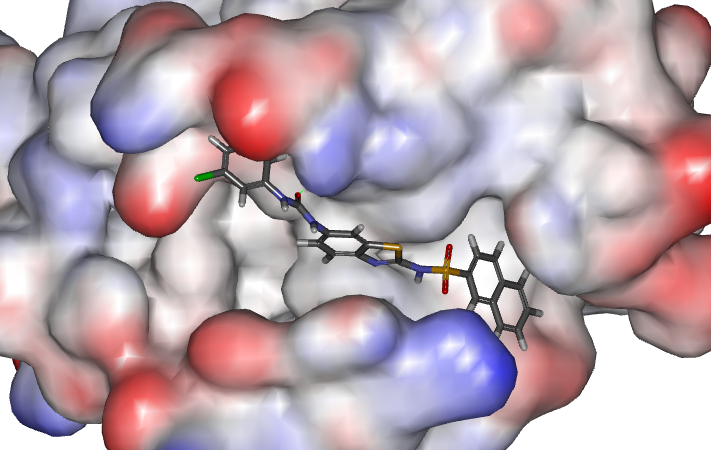 |
| **17c** | 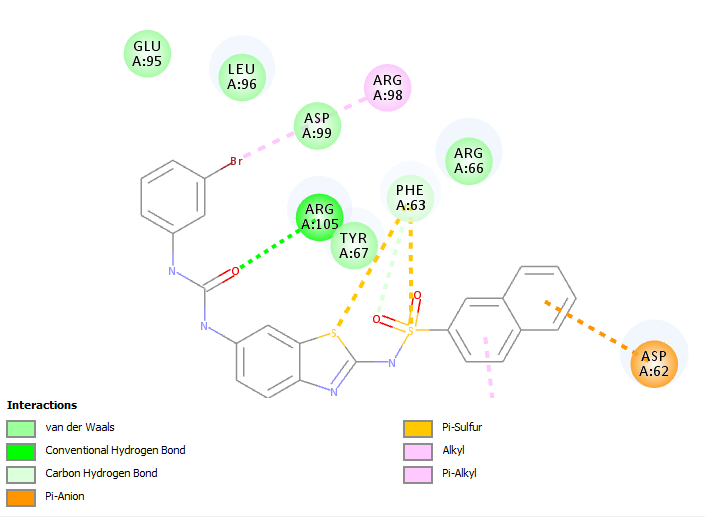 | **-44.77**  **HBA** Arg 105  **Pi- Sulfur** Phe 63  **Pi- Alkyl** Arg 98 | | 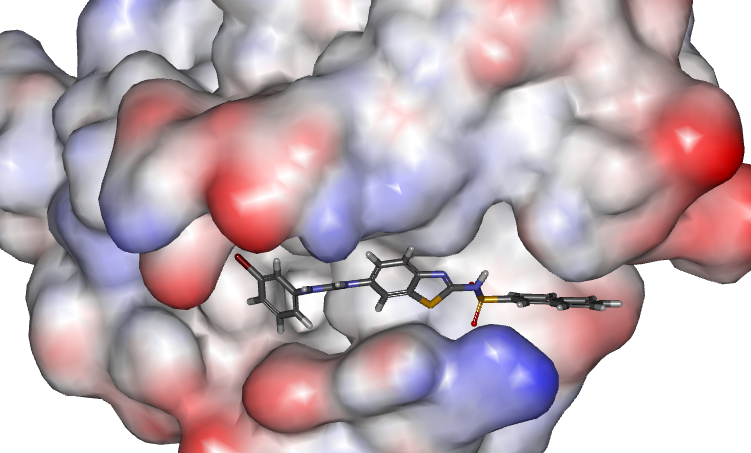 |
| **17d** | 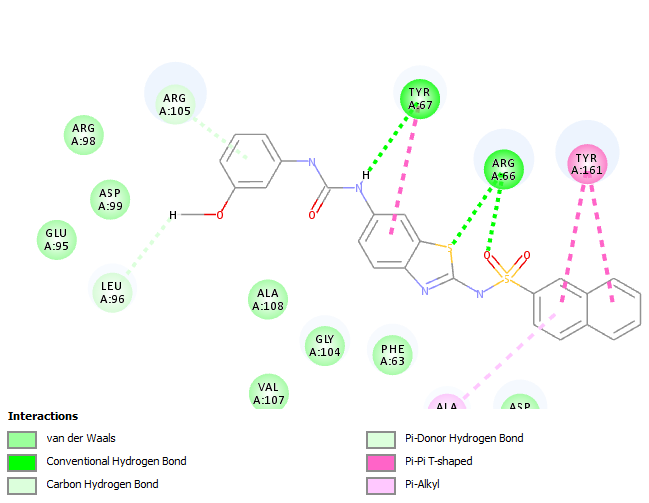 | **-40.31**  **HBA** Arg 66  **HBD** Tyr 67  **Pi-Pi** Tyr 67  **Pi-Pi** Tyr 161 | | 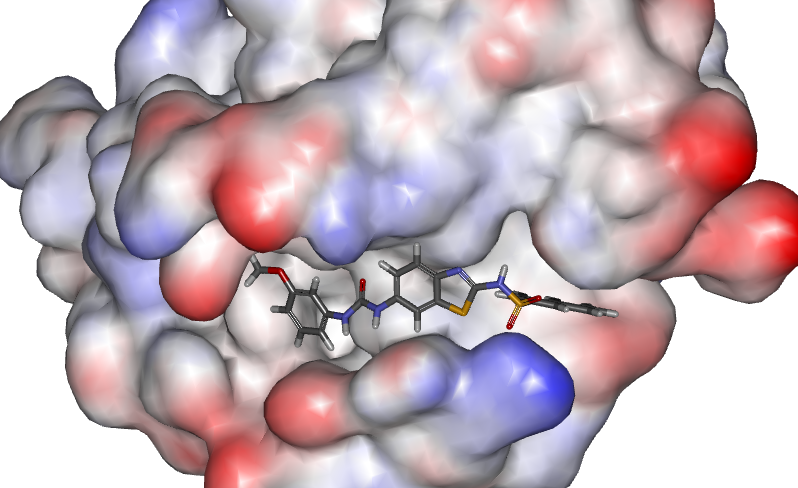 |
| **17e** | 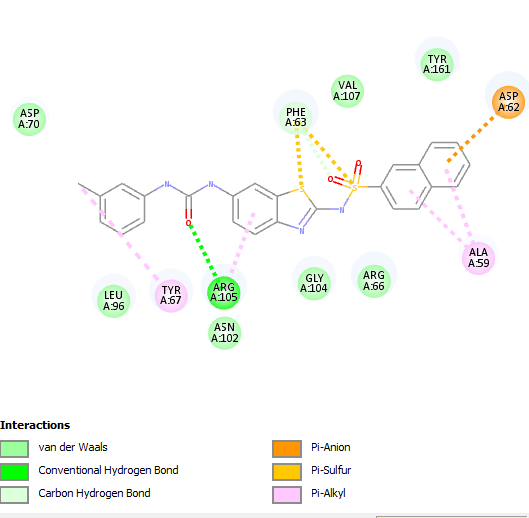 | **-40.44**  **HBA** Arg 105 | | 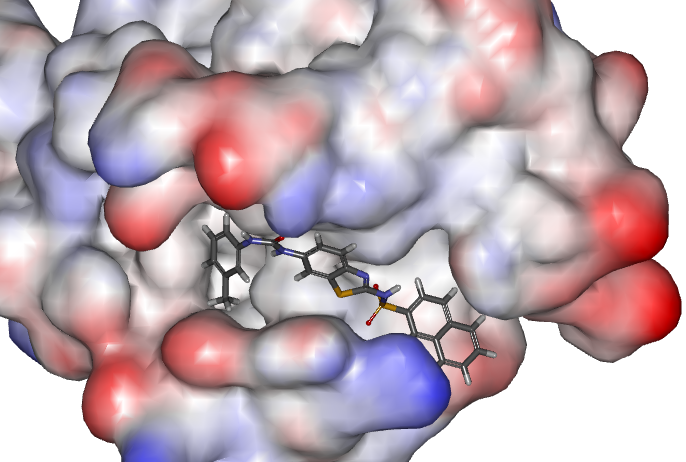 |
| **17f** | 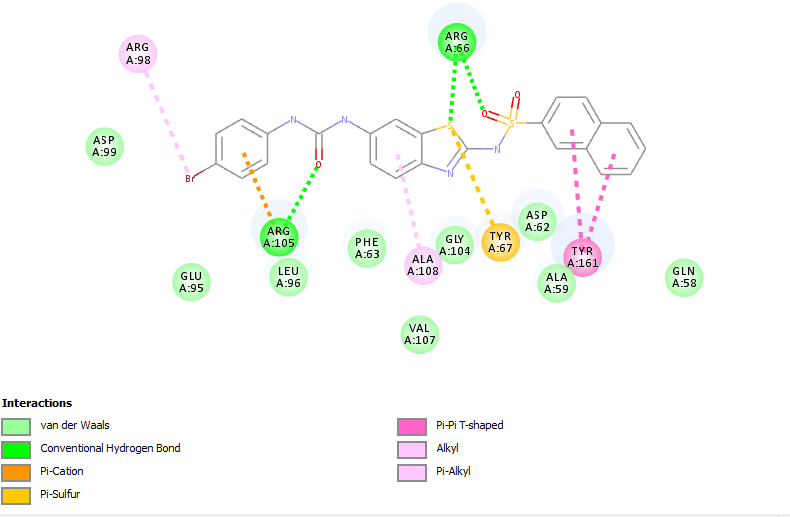 | **-41.69**  **HBA** Arg 66  **HBA** Arg 105  **Pi-Pi** Tyr 161  **Pi- Sulfur** Tyr 67  **Pi- Alkyl** Arg 98  **Pi- Alkyl** Ala 108 | | 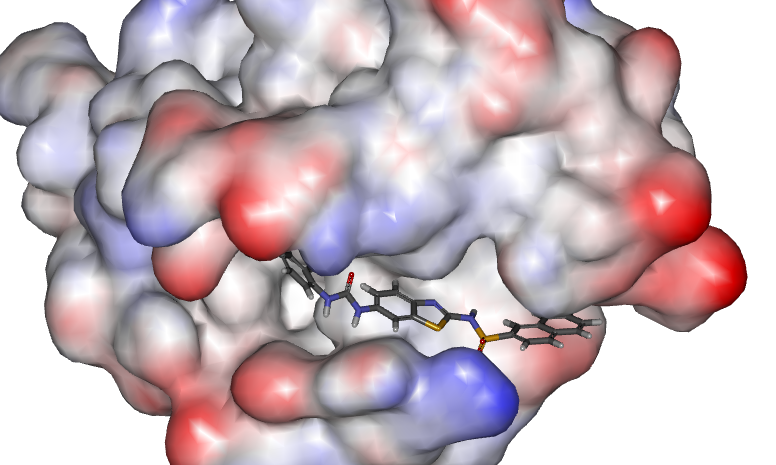 |
| **17g** | 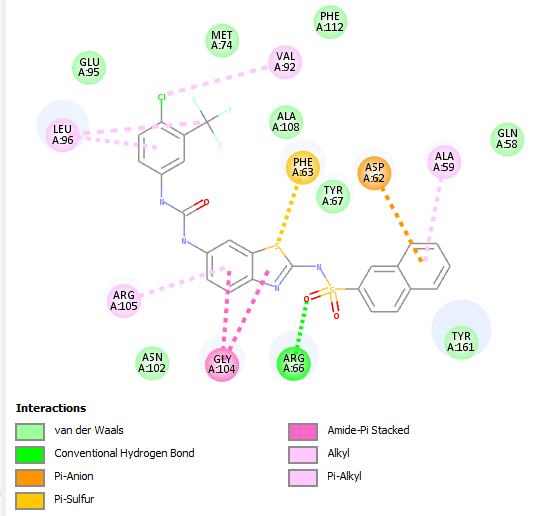 | **-46.49**  **HBA** Arg 66  **Pi- Sulfur** Phe 63  **Pi- Alkyl** Ala 59 | | 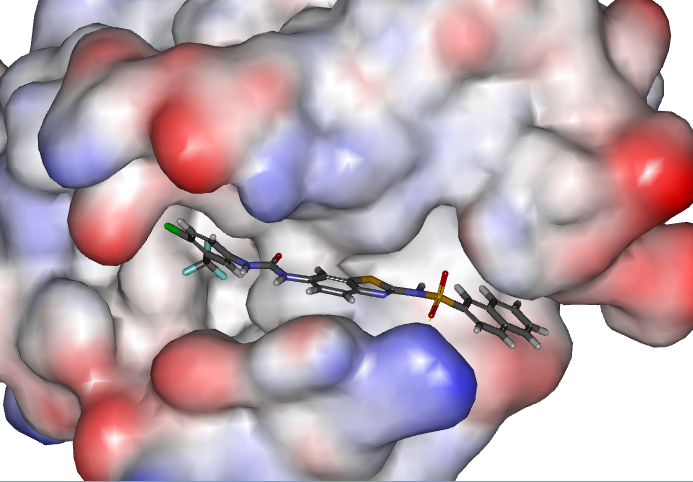 |
| **17h** | 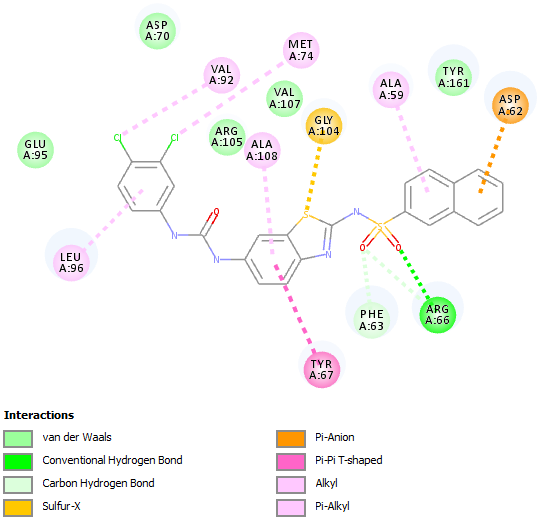 | **-44.95**  **HBA** Arg 66  **Pi-Pi** Tyr 67  **Pi- Alkyl** Ala 59  **Pi- Alkyl** Ala 108 | | 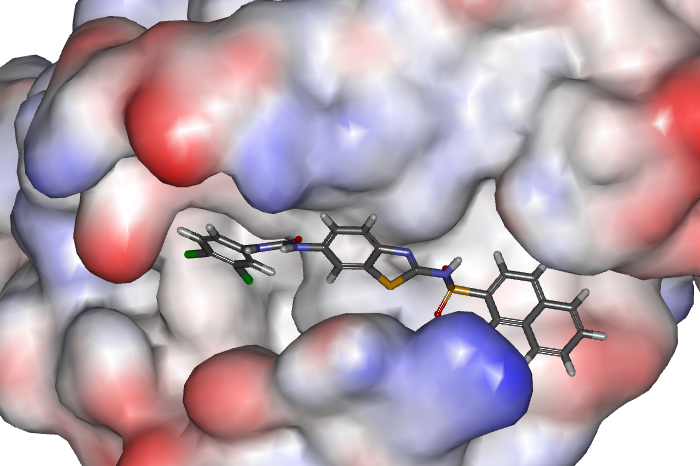 |
| **17i** | 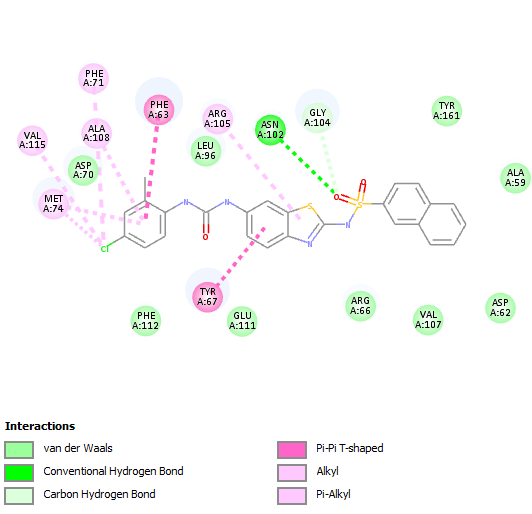 | **-42.20**  **HBA** Asn 102  **Pi-Pi** Phe 63  **Pi-Pi** Tyr 67  **Pi- Alkyl** Ala 108 | | 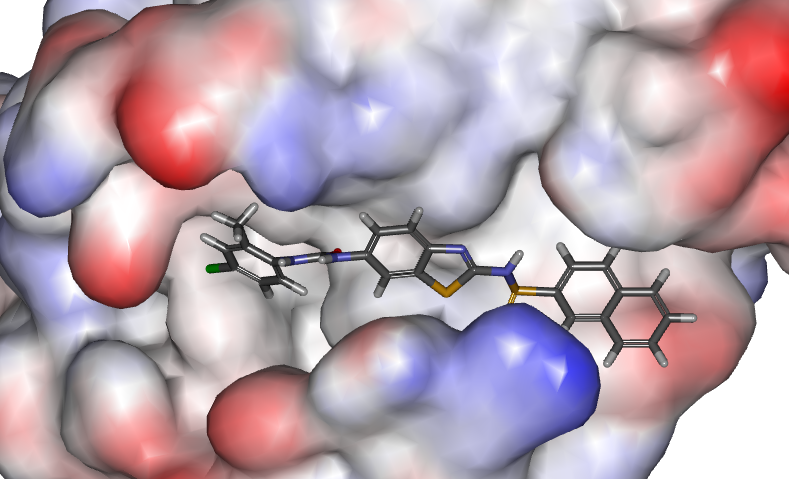 |
| **17j** | 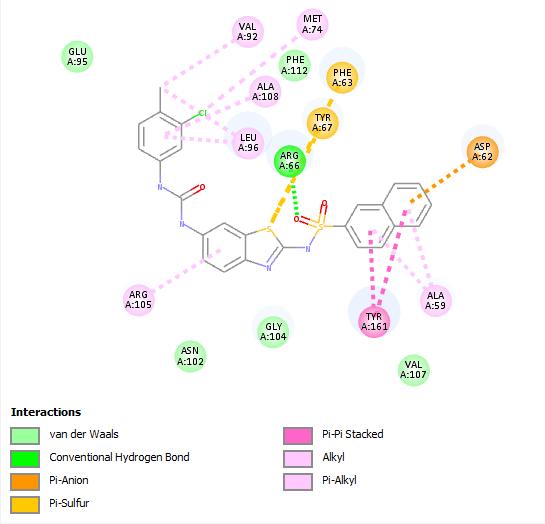 | **-40.51**  **HBA** Arg 66  **Pi-Pi** Tyr 161  **Pi- Alkyl** Ala 59  **Pi- Alkyl** Ala 108 | | 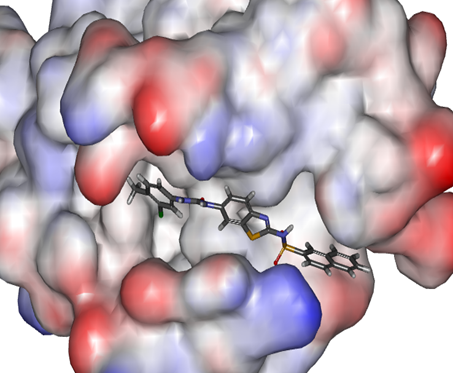 |

# Spectral Data of final compounds

***N*-(6-(4-Phenylpiperazine-1-carbonyl)benzo[*d*]thiazol-2-yl)naphthalene-2-sulfonamide (7a)**:

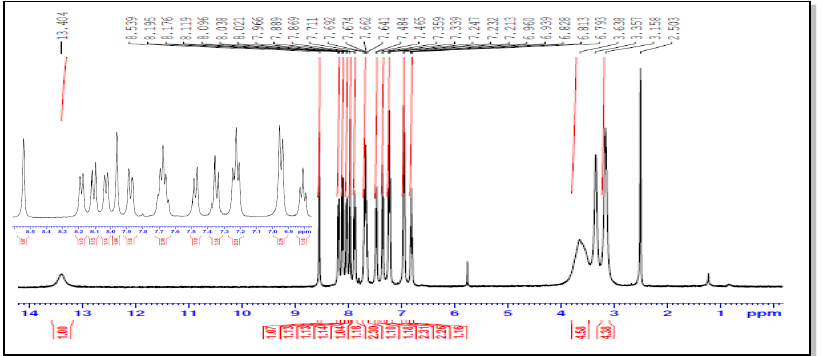


Figure S4: ^1^H-NMR spectrum for compound **7a**

***N*-(6-(4-(2-Fluorophenyl)piperazine-1-carbonyl)benzo[*d*]thiazol-2-yl)naphthalene-2-sulfonamide (7b):**

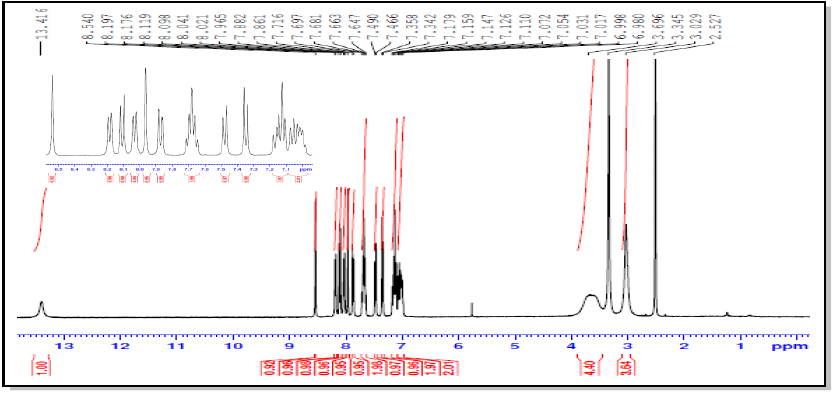


**Figure S5**: ^1^H-NMR spectrum for compound **7b**

***N*-(6-(4-(2-Methoxyphenyl)piperazine-1-carbonyl)benzo[*d*]thiazol-2-yl)naphthalene-2-sulfonamide (7c):**

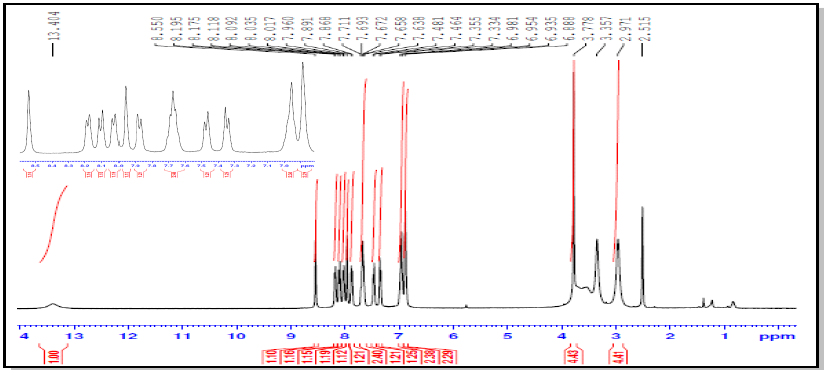


**Figure S6**: ^1^H-NMR spectrum for compound **7c**

***N*-(6-(4-(4-Chlorophenyl)piperazine-1-carbonyl)benzo[*d*]thiazol-2-yl)naphthalene-2-sulfonamide (7d):**

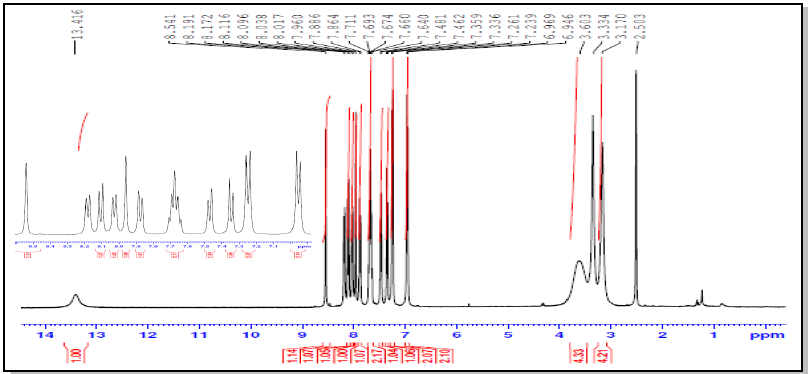


**Figure S7**: ^1^H-NMR spectrum for compound **7d**

***N*-(6-(4-(3,4-Dichlorophenyl)piperazine-1-carbonyl)benzo[*d*]thiazol-2-yl)naphthalene-2-sulfonamide (7e):**

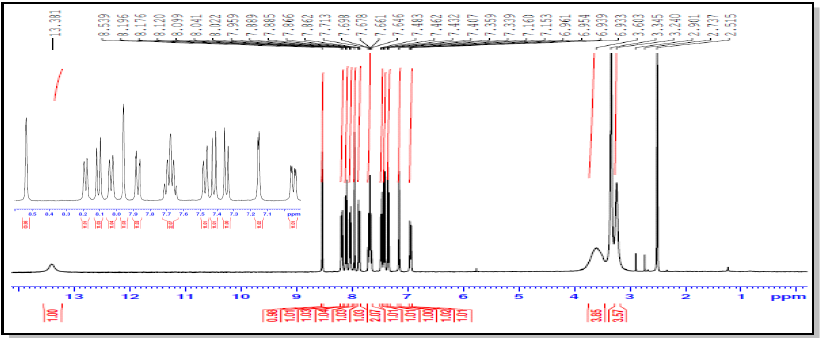


**Figure S8**: ^1^H-NMR spectrum for compound **7e**

***N*-(6-(4-Benzhydrylpiperazine-1-carbonyl)benzo[*d*]thiazol-2-yl)naphthalene-2-sulfonamide (7f):**

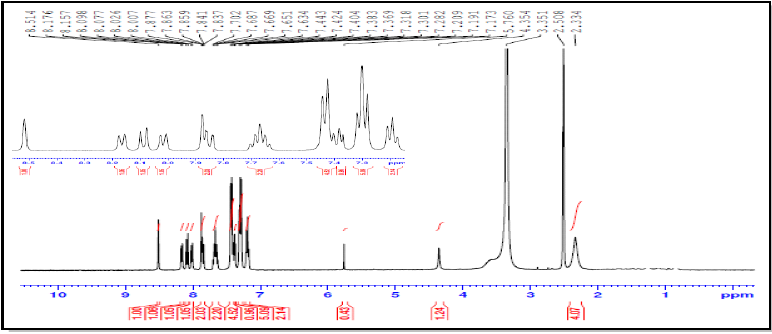


**Figure S9**: ^1^H-NMR spectrum for compound **7f**

**(*E*)-*N*-(6-(4-Cinnamylpiperazine-1-carbonyl)benzo[*d*]thiazol-2-yl)naphthalene-2-sulfonamide( 7g):**

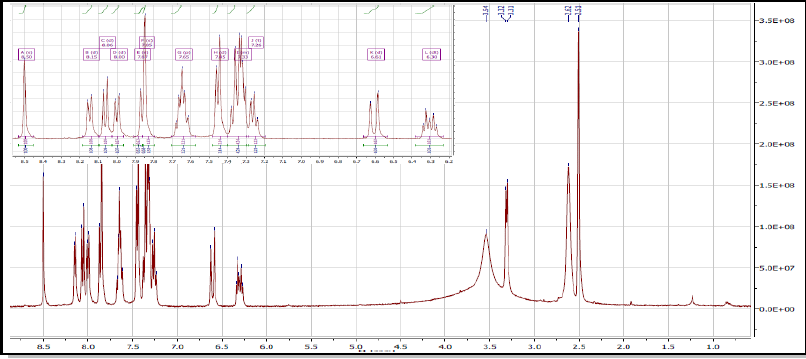


**Figure S10**: ^1^H-NMR spectrum for compound **7g**

***N*-(6-(4-(Benzo[*d*]dioxol-5-ylmethyl)piperazine-1-carbonyl)benzo[*d*]thiazol-2-yl)naphthalene-2-sulfonamide (7h):**

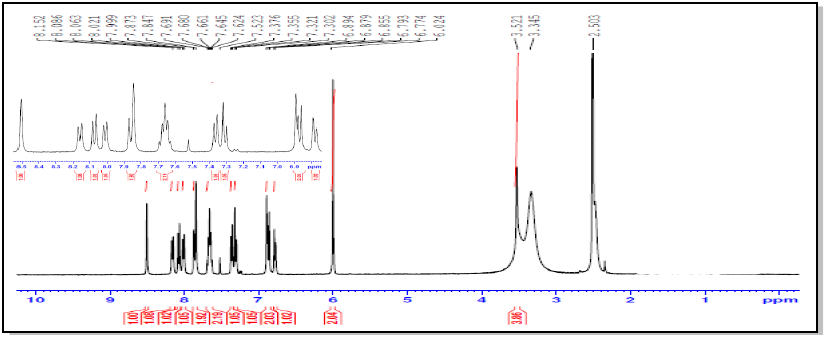


**Figure S11**: ^1^H-NMR spectrum for compound **7h**

***N*-(6-(4-(Tetrahydrofuran-2-carbonyl)piperazine-1-carbonyl)benzo[*d*]thiazol-2-yl)naphthalene-2-sulfonamide (7i) :**

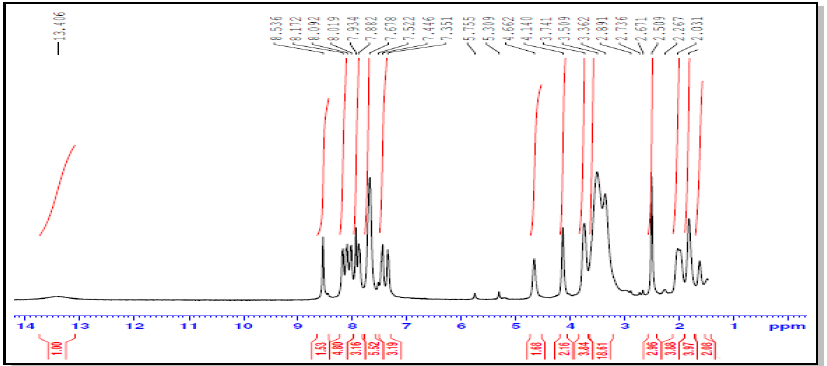


**Figure S12**: ^1^H-NMR spectrum for compound **7i**

***N*-(4-((3-Fluorobenzyl)oxy)phenyl)-2-(naphthalene-2-sulfonamido)benzo[*d*]thiazole-6-carboxamide (8a):**

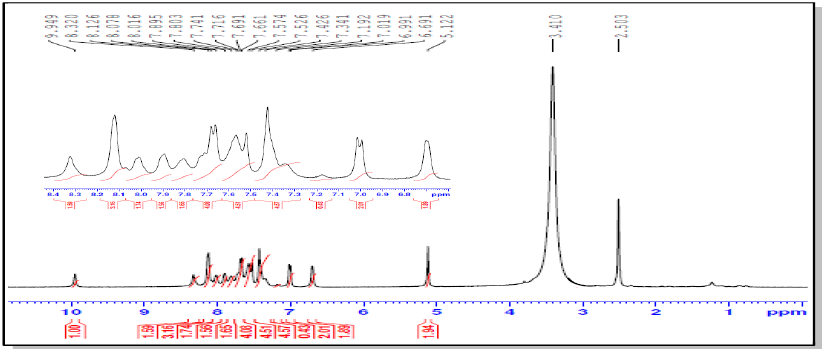


**Figure S13**: ^1^H-NMR spectrum for compound **8a**

***N*-(4-((3-Chlorobenzyl)oxy)phenyl)-2-(naphthalene-2-sulfonamido)benzo[*d*] thiazole-6-carboxamide (8b):**

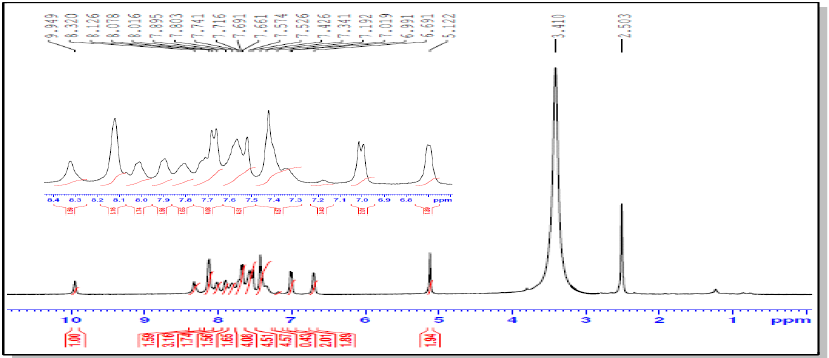


**Figure S14**: ^1^H-NMR spectrum for compound **8b**

***N*-(4-((4-Chlorobenzyl)oxy)phenyl)-2-(naphthalene-2-sulfonamido)benzo[*d*] thiazole-6-carboxamide (8c)**:

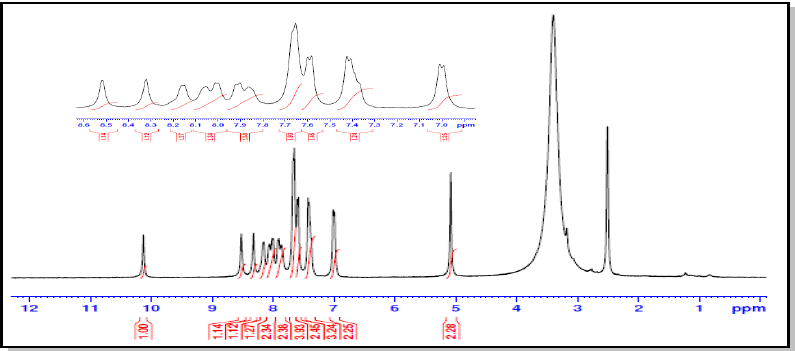


**Figure S15**: ^1^H-NMR spectrum for compound **8c**

***N*-(4-((4-Bromobenzyl)oxy)phenyl)-2-(naphthalene-2-sulfonamido)benzo[*d*] thiazole-6-carboxamide (8d)**:

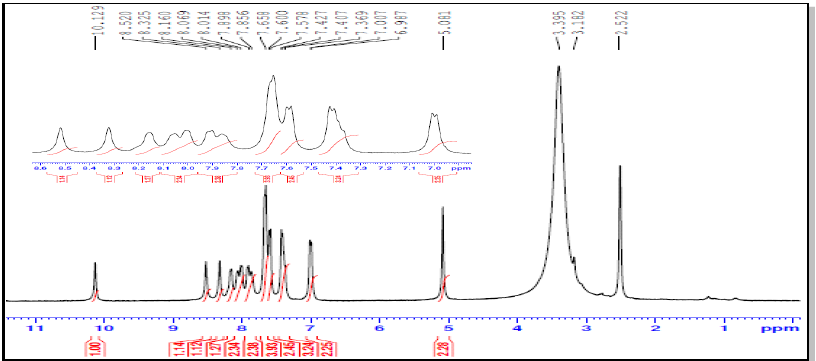


**Figure S16**: ^1^H-NMR spectrum for compound **8d**

***N*-(4-((4-Methylbenzyl)oxy)phenyl)-2-(naphthalene-2-sulfonamido)benzo[*d*] thiazole-6-carboxamide (8e):**

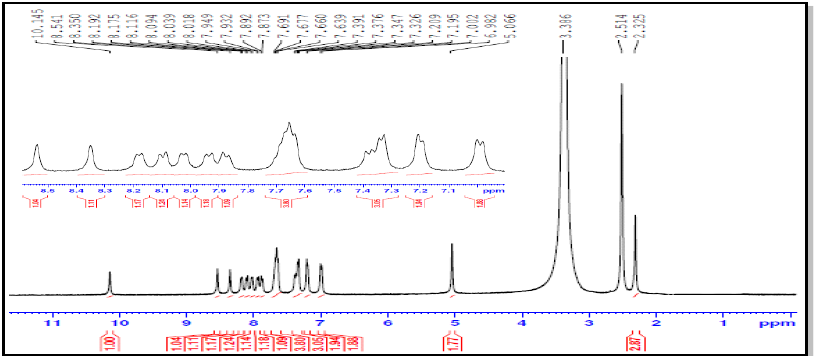


**Figure S17**: ^1^H-NMR spectrum for compound **8e**

***N*-(4-((4-Trifluormethylbenzyl)oxy)phenyl)-2-(naphthalene-2-sulfonamido)benzo [*d*]thiazole-6-carboxamide *)*8f)**:

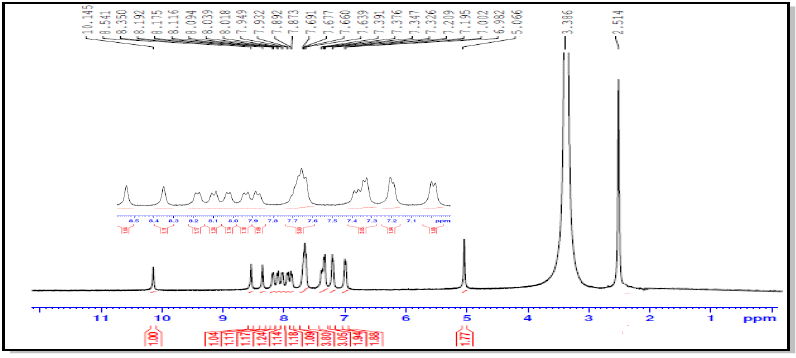


**Figure S18**: ^1^H-NMR spectrum for compound **8f**

***N*-(4-(4-Chloro-3-fluorophenoxy)phenyl)-2-(naphthalene-2-sulfonamido)benzo[*d*] thiazole-6-carboxamide (9a):**

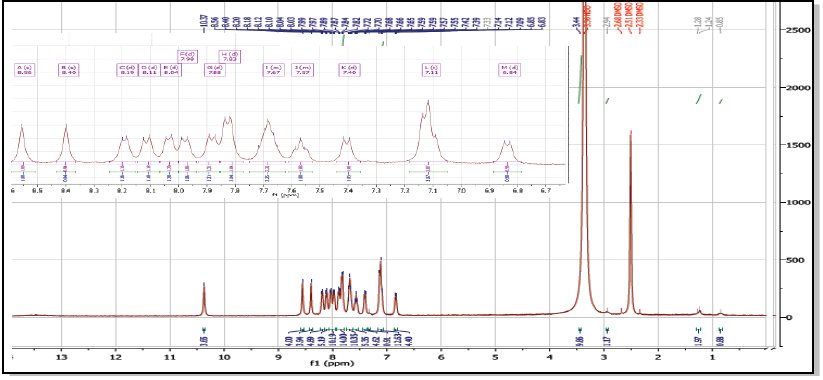


**Figure S19**: ^1^H-NMR spectrum for compound **9a**

***N*-(4-(4-Bromophenoxy)phenyl)-2-(naphthalene-2-sulfonamido)benzo[*d*]thiazole-6-carboxamide (9b):**

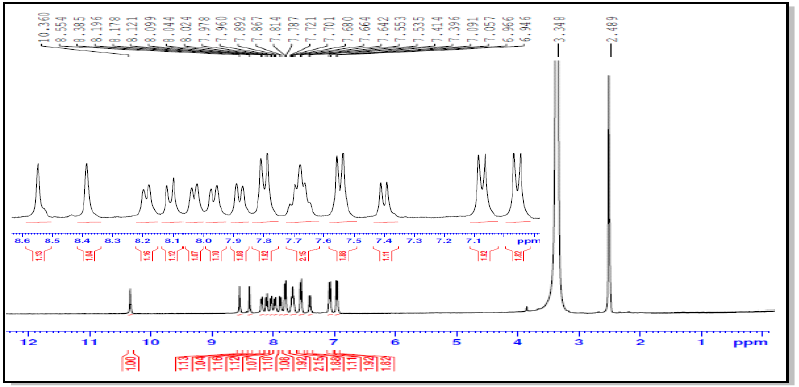


**Figure S20**: ^1^H-NMR spectrum for compound **9b**

***N*-(6-(4-(2-Methoxyphenyl) piperazine-1-carbonyl) benzo[*d*]thiazol-2-yl)-3-nitro-4-(phenethylamino)benzamide (12a):**

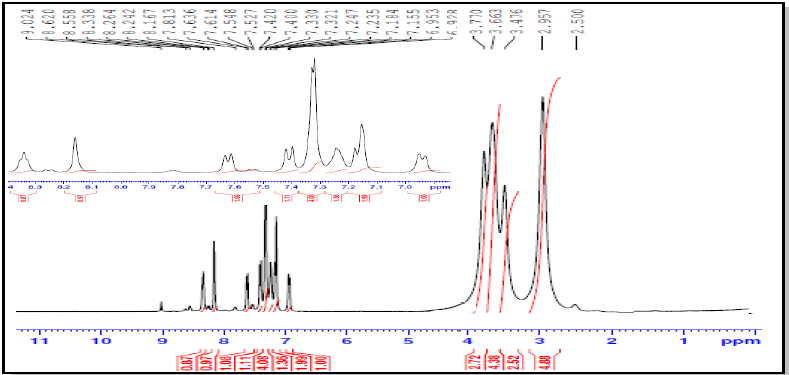


**Figure S21**: ^1^H-NMR spectrum for compound **12a**

***N*-(6-(4-(3,4-Dichlorophenyl)piperazine-1-carbonyl)benzo[*d*]thiazol-2-yl)-3-nitro-4-(phenethylamino)benzamide (12b):**

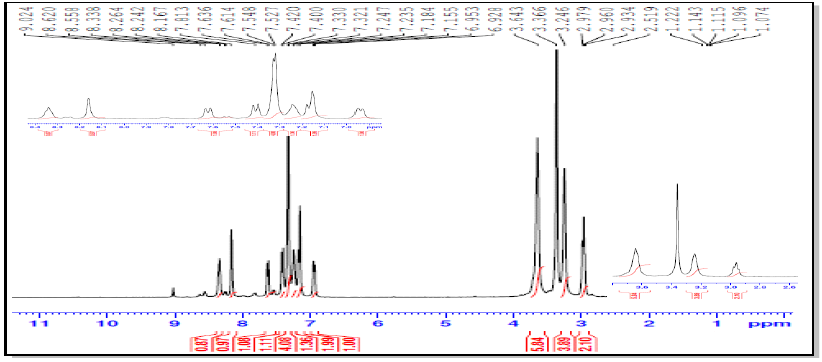


**Figure S22**: ^1^H-NMR spectrum for compound **12b**

***N*-(6-(4-Benzhydrylpiperazine-1-carbonyl)benzo[*d*]thiazol-2-yl)-3-nitro-(phenethylamino)benzamide (12c):**

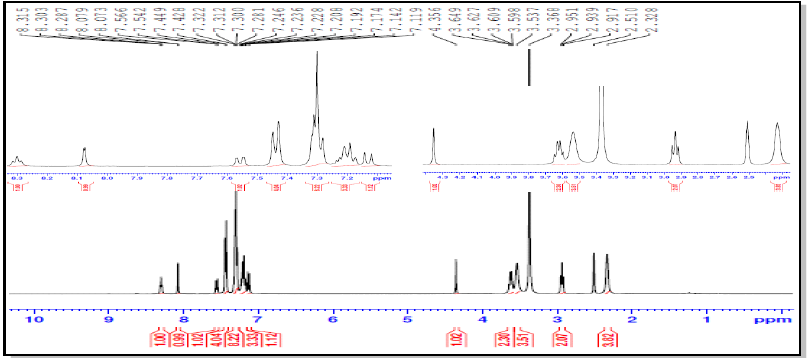


**Figure S23**: ^1^H-NMR spectrum for compound **12c**

**(*E*)-*N*-(6-(4-Cinnamylpiperazine-1-carbonyl)benzo*[d*]thiazol-2-yl)-3-nitro-4-(phenethylamino)benzamide (12d)**:

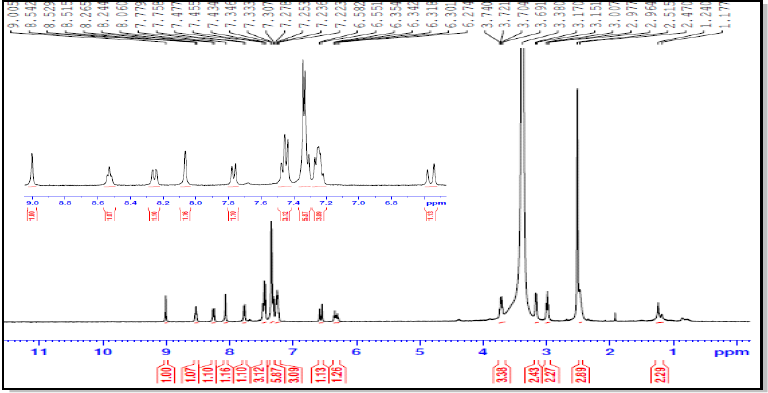


**Figure S24**: ^1^H-NMR spectrum for compound **12d**

***N*-(4-((3-Chlorobenzyl)oxy)phenyl)-2-(3-nitro-4-(phenethylamino)benzamido) benzo[*d*]thiazole-6-carboxamide (13a):**

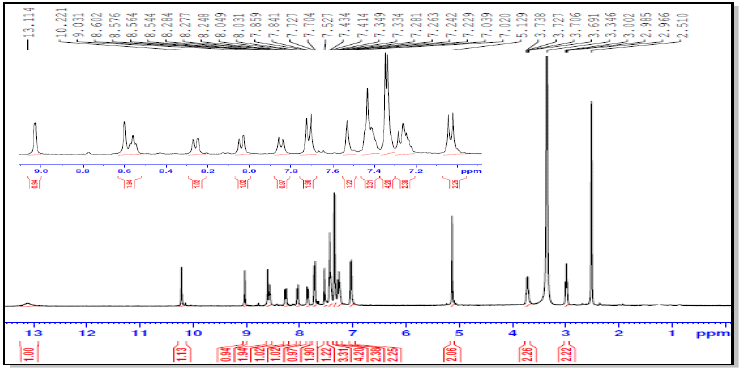


**Figure S25**: ^1^H-NMR spectrum for compound **13a**

***N*-(4-((4-Bromobenzyl)oxy)phenyl)-2-(3-nitro-4-(phenethylamino)benzamido) benzo[*d*]thiazole-6-carboxamide (13b):**

**Figure S26**: ^1^H-NMR spectrum for compound **13b**

***N*-(4-((4-Methylbenzyl)oxy)phenyl)-2-(3-nitro-4-(phenethylamino)benzamido)benzo[*d*]thiazole-6-carboxamide (13c):**

**Figure S27**: ^1^H-NMR spectrum for compound **13c**

***N*-(4-((4-Trifluoromethylbenzyl)oxy)phenyl)-2-(3-nitro-4-(phenethylamino)benzamido)benzo[*d*]thiazole-6-carboxamide (13d)**:

**Figure S28**: ^1^H-NMR spectrum for compound **13d**

***N*-(4-(4-Chloro-3-fluorophenoxy)phenyl)-2-(3-nitro-4-(phenethylamino) benzamido)benzo[*d*]thiazole-6-carboxamide (14a):**

**Figure S29**: ^1^H-NMR spectrum for compound **14a**

***N*-(4-(4-Bromophenoxy)phenyl)-2-(3-nitro-4-(phenethylamino)benzamido benzo[*d*]thiazole-6-carboxamide (14b):**

**Figure S30**: ^1^H-NMR spectrum for compound **14b**

***N*-(6-(3-Phenylureido)benzo[*d*]thiazol-2-yl)naphthalene-2-sulfonamide(17a):**

**Figure S31**: ^1^H-NMR spectrum for compound **17a**

***N*-(6-(3-(3-Chlorophenyl)ureido)benzo[*d*]thiazol-2-yl)naphthalene-2-sulfonamide (17b):**

**Figure S32**: ^1^H-NMR spectrum for compound **17b**

***N*-(6-(3-(3-Bromophenyl)ureido)benzo[*d*]thiazol-2-yl)naphthalene-2-sulfonamide (17c):**

**Figure S33**: ^1^H-NMR spectrum for compound **17c**

***N*-(6-(3-(3-Methoxyphenyl)ureido)benzo[*d*]thiazol-2-yl)naphthalene-2-sulfonamide (17d):**

**Figure S34**: ^1^H-NMR spectrum for compound **17d**

***N*-(6-(3-(*m*-Tolyl)ureido)benzo[*d*]thiazol-2-yl)naphthalene-2-sulfonamide (17e):**

**Figure S35**: ^1^H-NMR spectrum for compound **17e**

***N*-(6-(3-(4-Bromophenyl)ureido)benzo[*d*]thiazol-2-yl)naphthalene-2-sulfonamide (17f) :**

**Figure S36**: ^1^H-NMR spectrum for compound **17f**

***N*-(6-(3-(4-Chloro-3-(trifluoromethyl)phenyl)ureido)benzo[*d*]thiazol-2-yl)naphthalene-2-sulfonamide (17g):**

**Figure S37**: ^1^H-NMR spectrum for compound **17g**

***N*-(6-(3-(3,4-Dichlorophenyl)ureido)benzo[*d*]thiazol-2-yl)naphthalene-2-sulfonamide (17h):**

**Figure S38**: ^1^H-NMR spectrum for compound **17h**

***N*-(6-(3-(4-Chloro-2-methylphenyl)ureido)benzo[*d*]thiazol-2-yl)naphthalene-2-sulfonamide (17i):**

**Figure S39**: ^1^H-NMR spectrum for compound **17i**

***N*-(6-(3-(3-Chloro-4-methylphenyl)ureido)benzo[*d*]thiazol-2-yl)naphthalene-2-sulfonamide (17j):**

**Figure S40**: ^1^H-NMR spectrum for compound **17j**

**5-NCI Assay protocol**

The human tumor cell lines of the cancer screening panel were grown in RPMI 1640 medium containing 5% fetal bovine serum and 2 mM L-glutamine. For a typical screening experiment, cells are inoculated into 96 well microtiter plates in 100 µL at plating densities ranging from 5,000 to 40,000 cells/well depending on the doubling time of individual cell lines. After cell inoculation, the microtiter plates are incubated at 37° C, 5 % CO_2_, 95 % air and 100 % relative humidity for 24 hrs prior to addition of experimental drugs. After 24 hrs, two plates of each cell line are fixed *in situ* with trichloroacetic acid (TCA), to represent a measurement of the cell population for each cell line at the time of drug addition (Tz). Experimental drugs are solubilized in dimethyl sulfoxide at 400-fold the desired final maximum test concentration and stored frozen prior to use. At the time of drug addition, an aliquot of frozen concentrate is thawed and diluted to twice the desired final maximum test concentration with complete medium containing 50 µg/mL gentamicin. Additional four, 10-fold or ½ log serial dilutions are made to provide a total of five drug concentrations plus control. Aliquots of 100 µl of these different drug dilutions are added to the appropriate microtiter wells already containing 100 µl of medium, resulting in the required final drug concentrations.

Following drug addition, the plates are incubated for an additional 48 hrs at 37°C, 5 % CO_2_, 95 % air, and 100 % relative humidity. For adherent cells, the assay is terminated by the addition of cold TCA. Cells are fixed *in situ* by the gentle addition of 50 µl of cold 50 % (w/v) TCA (final concentration, 10 % TCA) and incubated for 60 minutes at 4°C. The supernatant is discarded, and the plates are washed five times with tap water and air dried. Sulforhodamine B (SRB) solution (100 µl) at 0.4 % (w/v) in 1 % acetic acid is added to each well, and plates are incubated for 10 minutes at room temperature. After staining, unbound dye is removed by washing five times with 1 % acetic acid and the plates are air dried. Bound stain is subsequently solubilized with 10 mMtrizma base, and the absorbance is read on an automated plate reader at a wavelength of 515 nm. For suspension cells, the methodology is the same except that the assay is terminated by fixing settled cells at the bottom of the wells by gently adding 50 µl of 80 % TCA (final concentration, 16 % TCA).

***Data analysis***:

Using the seven absorbance measurements [time zero, (Tz), control growth, (C), and test growth in the presence of drug at the five concentration levels (Ti)], the percentage growth is calculated at each of the drug concentrations levels. Percentage growth inhibition is calculated as:

**[(Ti-Tz)/(C-Tz)] x 100 for concentrations for which Ti > / = Tz**

**[(Ti - Tz) / Tz] x 100 for concentrations for which Ti < Tz**. ^11^

# 6-References

1. Sydnes, M.O. and M. Isobe, *Synthesis of the second generation photoaffinity probes of tautomycin.* Tetrahedron, 2007. **63**(12): p. 2593-2603.

2. Gates, K.S. and R.B. Silverman, *5-(Aminomethyl)-3-aryl-2-oxazolidinones. A novel class of mechanism-based inactivators of monoamine oxidase B.* Journal of the American Chemical Society, 1990. **112**(25): p. 9364-9372.

3. Defrenza, I., et al., *1,3‐Benzothiazoles as Antimicrobial Agents.* Journal of Heterocyclic Chemistry, 2015. **52**(6): p. 1705-1712.

4. Catalano, A., et al., *2-Aminobenzothiazole derivatives: search for new antifungal agents.* Eur J Med Chem, 2013. **64**: p. 357-64.

5. Tran, D.N., et al., *Flow chemistry as a discovery tool to access sp(2)-sp(3) cross-coupling reactions via diazo compounds.* Chem Sci, 2015. **6**(2): p. 1120-1125.

6. Hossam, M., et al., *Discovery of anilino-furo[2,3-d]pyrimidine derivatives as dual inhibitors of EGFR/HER2 tyrosine kinase and their anticancer activity.* Eur J Med Chem, 2018. **144**: p. 330-348.

7. Kawakita, Y., et al., *Design and synthesis of pyrrolo[3,2-d]pyrimidine human epidermal growth factor receptor 2 (HER2)/epidermal growth factor receptor (EGFR) dual inhibitors: exploration of novel back-pocket binders.* J Med Chem, 2012. **55**(8): p. 3975-91.

8. Cortese, N.A. and R.F. Heck, *Palladium catalyzed reductions of halo- and nitroaromatic compounds with triethylammonium formate.* The Journal of Organic Chemistry, 1977. **42**(22): p. 3491-3494.

9. Cai, J., et al., *Design and synthesis of novel 4-benzothiazole amino quinazolines Dasatinib derivatives as potential anti-tumor agents.* European Journal of Medicinal Chemistry, 2013. **63**: p. 702-712.

10. González-Alvarez, M., et al., *Development of novel copper(II) complexes of benzothiazole- N-sulfonamides as protective agents against superoxide anion. Crystal structures of [Cu( N-2-(4-methylbenzothiazole)benzenesulfonamidate)(2)(py)(2)] and [Cu( N-2-(6-nitrobenzothiazole)naphthalenesulfonamidate)(2)(py)(2)].* J Biol Inorg Chem, 2003. **8**(1-2): p. 112-20.

11. Scott, R. B. Cancer Chemotherapy—The First Twenty-five Years. *Br. Med. J.* **4,** 259–265 (1970).
